# Interfering Transposable Elements: IS *Xoo* 15 Transposase as a First‐in‐Class Antibacterial Target Against *Xanthomonas oryzae* pv. *oryzae*

**DOI:** 10.1111/mpp.70169

**Published:** 2025-11-16

**Authors:** Funeng Lu, Ting Liu, Tangbing Yang, Ziming Wang, Jianzhuan Li, Chunni Zhao, Huan Wu, Deyu Hu, Baoan Song

**Affiliations:** ^1^ State Key Laboratory of Green Pesticide, Center for R&D of Fine Chemicals Guizhou University Guiyang China

**Keywords:** activity‐based protein profiling, agrochemical, bacterial leaf blight, transposable element, transposase

## Abstract

Current challenges in controlling phytopathogenic bacteria lie in widespread chemical resistance, biosafety concerns, and the scarcity of novel biomacromolecule targets. While transposable elements have emerged as critical drivers of genetic variability and virulence in plant pathogens, their potential as druggable targets remains unexplored. Here, we report the first discovery of IS*Xoo*15 transposase in *Xanthomonas oryzae* pv. *oryzae* (Xoo) as the bactericidal receptor for J9, a pyrimidine‐substituted pleuromutilin derivative. In vitro assays demonstrate J9's superior anti‐Xoo activity, with an EC_50_ of 0.12 mg/L—significantly lower than commercial agents thiodiazole copper (86.39 mg/L) and zinc thiazole (26.15 mg/L). In vivo pot trials reveal enhanced curative and protective efficacy of J9 against rice bacterial leaf blight compared to these metal‐based controls. A photoaffinity probe, P‐J9, is synthesised and coupled with activity‐based protein profiling to unequivocally identify IS*Xoo*15 transposase (encoded by *PXO_03433*) as J9's specific target. Reverse transcription‐quantitative PCR confirmed significant downregulation of *PXO_03433* expression in J9‐treated Xoo. Physiological and virulence‐related functional analyses of a homologous recombination‐mediated *PXO_03433*‐knockout strain (ΔPXO_03433) showed markedly attenuated virulence and impaired pathogenicity. Conversely, *PXO_03433*‐complemented strain CΔPXO_03433 possessed substantial restoration of pathogenicity‐related traits. Proteomic profiling revealed significant downregulation of pathways associated with DNA repair, recombination and binding proteins in both J9‐treated and mutant strains. IS*Xoo*15 transposase may serve as a key regulator in enabling the homeostasis of the DNA metabolic network in the bacteria. This study provides pioneering evidence for targeting bacterial transposases as a novel antibacterial strategy, establishing a foundation for effective management of phytopathogenic bacteria.

## Graphical abstract

The study identifies the previously unknown transposase IS*Xoo*15 as an antiphytobacterial target. By constructing mutant strains, this enzyme's essential role in regulating bacterial pathogenicity and virulence is revealed.

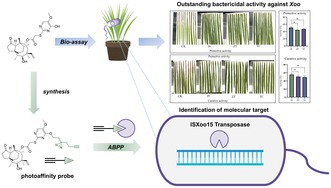

## 1 Introduction

The genus *Xanthomonas* comprises a group of gram‐negative bacterial pathogens responsible for devastating diseases in economically critical crops including rice ( *Oryza sativa* ), crucifers (*Brassica* spp.), citrus (*Citrus* spp.) and tomato ( *Solanum lycopersicum* ) (Mansfield et al. 2012). These pathogens exhibit remarkable host specificity and adaptability, resulting in crop failures (Ryan et al. 2011). Of these, *Xanthomonas oryzae* pv. *oryzae* (Xoo), the causal agent of bacterial leaf blight (BLB) in rice, disrupts vascular tissues, leading to wilting, leaf necrosis and premature plant death, threatening global food security for over half the world's population (Niño‐Liu et al. 2006; González et al. 2012). The pathogenicity of *Xanthomonas* spp. relies on virulence determinants such as type III secretion systems, effector proteins and extracellular polysaccharides, which collectively suppress host immunity and promote tissue colonisation (Rossier et al. 1999; Kay and Bonas 2009; Rudolph et al. 1994). Despite advances in resistant cultivar breeding and chemical control, management remains challenging due to the rapid evolution of pathogen strains, widespread antibiotic resistance, and the limited durability of host resistance genes (Schandry et al. 2018; Vera Cruz et al. 2000; Xu et al. 2013). Traditional bactericides, involving inorganic chemicals (e.g., copper hydroxide, copper sulphate), organic–Cu complexes and antibiotics like streptomycin, face declining efficacy and environmental concerns (Scherer et al. 2013; Griffin et al. 2017; Kiaune and Sing 2011). While zinc thiazole (ZT) shows enhanced environmental compatibility relative to copper‐based alternatives, its structural core (the thiazole moiety) shares a similarity with *N*,*N*'‐methylene‐bis(2‐amino‐1,3,4‐thiadiazole) (Bis‐A‐TDA), a compound banned due to its association with biosafety hazards. Furthermore, studies have suggested that zinc thiazole may possess thyroid‐disrupting side effects (Honglian et al. 2020; Yang et al. 2013). These underscore the urgent need for novel antimicrobial strategies targeting conserved bacterial vulnerabilities.

A critical scientific gap in the chemical control of phytopathogenic bacteria stems from the scarcity of well‐defined receptors or mechanistically characterised modes of action (MOAs). To date, only limited MOAs of commercial bactericides have been partially elucidated. For example, copper pesticides primarily exert antimicrobial activity through the release of cuprous and cupric ions, which compromise membrane integrity, facilitate cellular penetration and trigger oxidative stress via endogenous reactive oxygen species (ROS) generation (Salah et al. 2021). Bismerthiazol suppresses virulence in Xoo by dual inhibition of the histidine utilisation pathway and quorum‐sensing networks (Liang et al. 2018). However, the systematic identification of specific, druggable bacterial targets, particularly those essential for pathogen survival and adaptation yet exhibiting minimal structural homology to non‐target organisms, remains a huge barrier (Zhang, He, et al. 2024; Zhang, Zhang, et al. 2024). This knowledge deficit fundamentally constrains the development of next‐generation bactericides with enhanced target specificity, minimised ecological disruption and reduced propensity for resistance evolution.

Emerging research highlights the role of insertion sequences (ISs) and other transposable elements (TEs) in driving genetic plasticity and virulence in *Xanthomonas* spp. (Xu et al. 2013; Oliveira et al. 2018; Lipszyc et al. 2022; Fernandes et al. 2024; Razavi et al. 2020). For instance, *Xanthomonas arboricola* pv. *juglandis* may acquire and disseminate virulence factors through ISs, with ISs implicated in the acquisition and diversification of type III effector proteins in certain strains (Assis et al. 2021). Additionally, Ferreira et al. (2015) confirmed that transposition events mediated by Tn*3*‐like elements are critical determinants in the evolution and emergence of pathogenicity in *Xanthomonas* species (Xu et al. 2013). These pioneering studies established a pivotal basis for novel antibacterial strategies targeting TE functionality. Transposases, the enzymes catalysing TE excision and integration, are central to this adaptive machinery. By disrupting transposase activity, small molecules could be developed to suppress TE mobility, thereby attenuating bacterial virulence, stabilising genomic architecture and impairing adaptive traits. However, to our knowledge, no small molecules have been reported to date that specifically target transposase in *Xanthomonas* to achieve these outcomes.

The present study reports the first application of activity‐based protein profiling (ABPP) to identify IS*Xoo*15 transposase in Xoo as the receptor for J9, a small molecule exhibiting exceptional antibacterial activity. We constructed a *PXO_03433* gene knockout strain and a complemented strain via homologous recombination and systematically investigated their impact on Xoo pathogenicity and virulence through comprehensive physiological, biochemical and virulence‐related functional analyses. We expect the research to establish a basis for developing transposase‐targeted small molecules to inhibit bacterial virulence effects mediated by insertion elements or suppress genetic variation mechanisms in pathogenic bacteria.

## 2 Results and Discussion

### 2.1 Design and Synthesis of Target Small‐Molecules

To develop compounds with potent antibacterial activity, we initiated a rational design strategy focusing on lead optimisation and functional group incorporation. Pleuromutilin, a natural antibacterial diterpene first isolated from *Pleurotus mutilus* and *Pleurotus passeckerianus* by Kavanagh et al. (1951) was selected as the lead scaffold with its established structure–activity relationship favouring C‐14 modifications for enhanced bioactivity (Hunt 2000; Novak and Shlaes 2010). Inspired by recent evidence demonstrating pyrimidine derivatives' broad antibacterial potential (Zhuang and Ma 2020; Ahmed et al. 2023), we introduced diverse substituted pyrimidine moieties to the C‐14 ester position of pleuromutilin, generating the J‐series compounds (Figure S1A). The synthetic route (Figure S1B) commenced with commercially available pleuromutilin, yielding intermediates B and C via reported method (Ai et al. 2016). Subsequent nucleophilic substitution of intermediate C with 2‐mercapto‐5‐methoxypyrimidin‐4‐ol afforded J9, whose hydroxyl group underwent further deprotonation and substitution to synthesise the remaining derivatives. This four‐step protocol achieved overall yields of 48.7%–55.3% under mild conditions. All target compounds were fully characterised by physicochemical properties, ^1^H NMR, ^13^C NMR and HRMS (Figures S3–S77).

### 2.2 Compound J9 Displays Excellent In Vitro and In Vivo Antibacterial Activity

**FIGURE 1:**
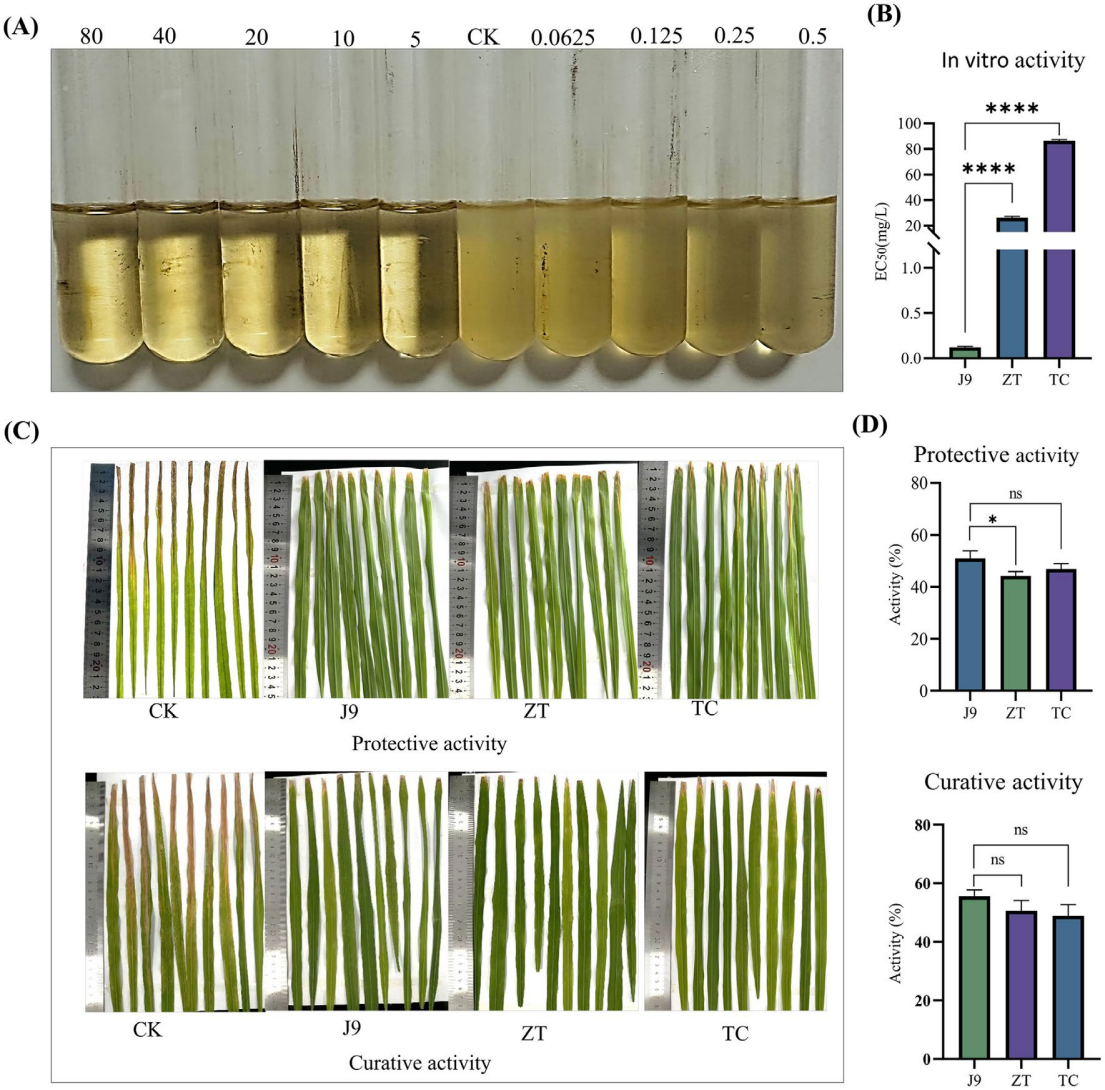
In vitro and in vivo antibacterial activity tests of J9 small molecule. (A,B) EC_50_ values of compound J9 and control compound. Five corresponding concentration gradients of J9 small molecules (1, 0.5, 0.25, 0.125, 0.0625 mg/L) were set to determine the EC_50_ value of the bacteria. (C,D) Protective and curative activity of compound J9 against rice bacterial leaf blight at 200 mg/L. The protective and curative activities of the J9 small molecule were determined under the control of a drug concentration of 200 mg/L and water with only dimethyl sulphoxide (DMSO) added. Protective activity: the chemical was sprayed on the leaves for 1 day first, and then the bacterial solution was inoculated. For therapeutic activity, the bacterial solution was inoculated for 1 day first, and then chemical was sprayed. The length of the disease spots on the rice leaves was counted 14 days after inoculation. Error bars indicate ± SD of the mean of three independent experiments (*n* = 3 biological replicates). Statistical *p*‐values were calculated using the one‐tailed paired samples *t* tests (*p < 0.05; ****p < 0.0001; ^ns^ *p* > 0.05 not significant).

With the synthesised compounds in hand, we next evaluated their in vitro antibacterial activity against Xoo using turbidimetric assays. Remarkably, all derivatives except J23 exhibited potent anti‐Xoo activity with EC_50_ values < 1 mg/L (Table S1). Compound J9 demonstrated exceptional efficacy (EC_50_ = 0.12 ± 0.02 mg/L), outperforming commercial controls ZT (EC_50_ = 26.15 mg/L) and thiodiazole‐copper (TC, EC_50_ = 86.39 mg/L) by > 200‐fold (Figure 1A,B). Building on this success, J9 was further measured in rice pot trials (200 mg/L) against bacterial leaf blight (BLB), with ZT and TC serving as positive controls. We were delighted to see that J9 displayed both curative (55.55%) and protective (50.96%) properties (Figure 1C,D; Table S2), which stand in contrast to ZT (50.66% and 44.23%) and TC (48.89% and 46.16%). These results suggest that J9 is an optimal candidate for subsequent study.

### 2.3 ABPP Technology Identifies Potential Molecular Targets of J9

**FIGURE 2:**
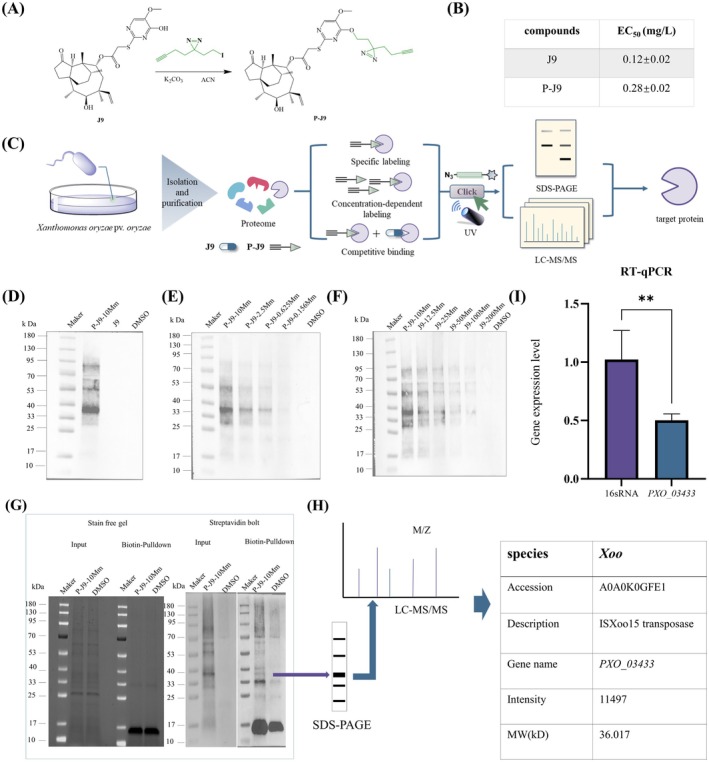
Analysis of the interaction between compound J9 and IS*Xoo*15 transposase. (A) The synthesis route of probe P‐J9. (B) Comparison of EC_50_ between probe P‐J9 and compound J9. Five corresponding concentration gradients (1, 0.5, 0.25, 0.125, 0.0625 mg/L) were set to determine the EC_50_ value of bacteria for the probe small molecule. (C) Capture route of the target protein. (D) Western blotting image. When the probe concentration was set at 2.5 mM, a band at 36.01 kDa could be labelled. Neither the negative control J9 small molecule nor the blank control dimethyl sulphoxide (DMSO) was labelled with this band. (E) Concentration‐dependent western blotting image. When the probe concentrations were set at 2.5, 0.625, 0.312 and 0.156 mM, a protein band of approximately 36.01 kDa could be labelled. As the concentration decreased, the protein band weakened significantly. The blank control DMSO was not labelled with bands. (F) Competitive western blotting image. The concentration of the probe was set at 12.5 mM. As the concentration of the J9 small molecule increased (0, 12.5, 25, 50, 100, 200 mM), the protein band at 36.01 kDa gradually weakened. No protein bands were labelled in the blank control DMSO. (G) Pull‐down experiment western blotting images. When the concentration of the probe small molecule was set at 10 mM, the magnetic beads could be enriched to the specific protein band at 36.01 kDa labelled in the ABPP experiment (H) LC–MS/MS analysis. (I) Expression level analysis of the *PXO_03433* gene. Values are means and standard errors of three independent experiments. Using the gene of 16S rRNA as the reference control, cDNA was synthesised from 1 μg RNA using PrimeScript reverse transcriptase (Takara), and quantitative PCR was performed using CFX real‐time PCR (Bio‐Rad) and TBGreen Fast qPCR mixture (Takara). The data were calculated using the $2^{-∆∆C_{T}}$ method. Error bars indicate ± SD of the mean of three independent experiments (*n* = 3 biological and technical replicates). Statistical *p*‐values were calculated using the one‐tailed paired samples *t* tests (**p < 0.01).

ABPP is a chemical proteomics strategy that uses functionalised probes to selectively label and characterise enzymatically active proteins in complex biological systems (Assis et al. 2021; Zhang, He, et al. 2024; Zhang, Zhang, et al. 2024). To elucidate the molecular target of J9, we employed the ABPP technique. A photoaffinity probe (P‐J9) was synthesised via a one‐step reaction of J9 with 3‐(but‐3‐yn‐1‐yl)‐3‐(2‐iodoethyl)‐3*H*‐diazirine (Figure 2A and Figure S2). Because structural modifications may influence compound activity and potentially lead to off‐target effects, we further evaluated the anti‐Xoo activity of P‐J9. Fortunately, P‐J9 showed comparable potency to J9 with an EC_50_ value of 0.28 ± 0.02 mg/L versus 0.12 ± 0.02 mg/L for J9 (Figure 2B), indicating preserved bioactivity and suitability for following ABPP experiments. We then performed the ABPP workflow on the Xoo proteome including specific labelling, concentration dependence, competitive inhibition and pull‐down assays (Figure 2C). At a probe concentration of 10 mM, P‐J9 specifically labelled a distinct protein band at approximately 36.5 kDa, while neither the negative control (J9) nor the blank control (DMSO) exhibited labelling at this molecular weight, confirming target specificity (Figure 2D). In concentration‐dependent labelling experiments, P‐J9 demonstrated dose‐responsive binding to a 36.5 kDa protein across tested concentrations (2.5, 0.625, 0.312 and 0.156 mM), with signal intensity diminishing inversely proportional to probe concentration (Figure 2E). Competitive inhibition assays revealed that increasing J9 concentrations (12.5–200 mM) progressively attenuated P‐J9 labelling at 36.01 kDa, indicating competitive binding between the probe and compound J9 for the same target (Figure 2F). Subsequent streptavidin magnetic bead pull‐down of P‐J9‐interacting proteins (Figure 2G), followed by LC–MS/MS analysis (Figure 2H), identified IS*Xoo*15 transposase (predicted molecular weight: 36.1 kDa) as a potential receptor protein. To validate these results, reverse transcription‐quantitative PCR (RT‐qPCR) analysis demonstrated significant downregulation of the *PXO_03433* gene (encoding IS*Xoo*15 transposase) in J9‐treated Xoo strains compared to 16S rRNA controls (Figure 2I). Collectively, these findings establish IS*Xoo*15 transposase as the specific molecular target of J9 in Xoo.

### 2.4 J9 Exhibits Strong Binding Affinity for IS*Xoo*15 Transposase

**FIGURE 3:**
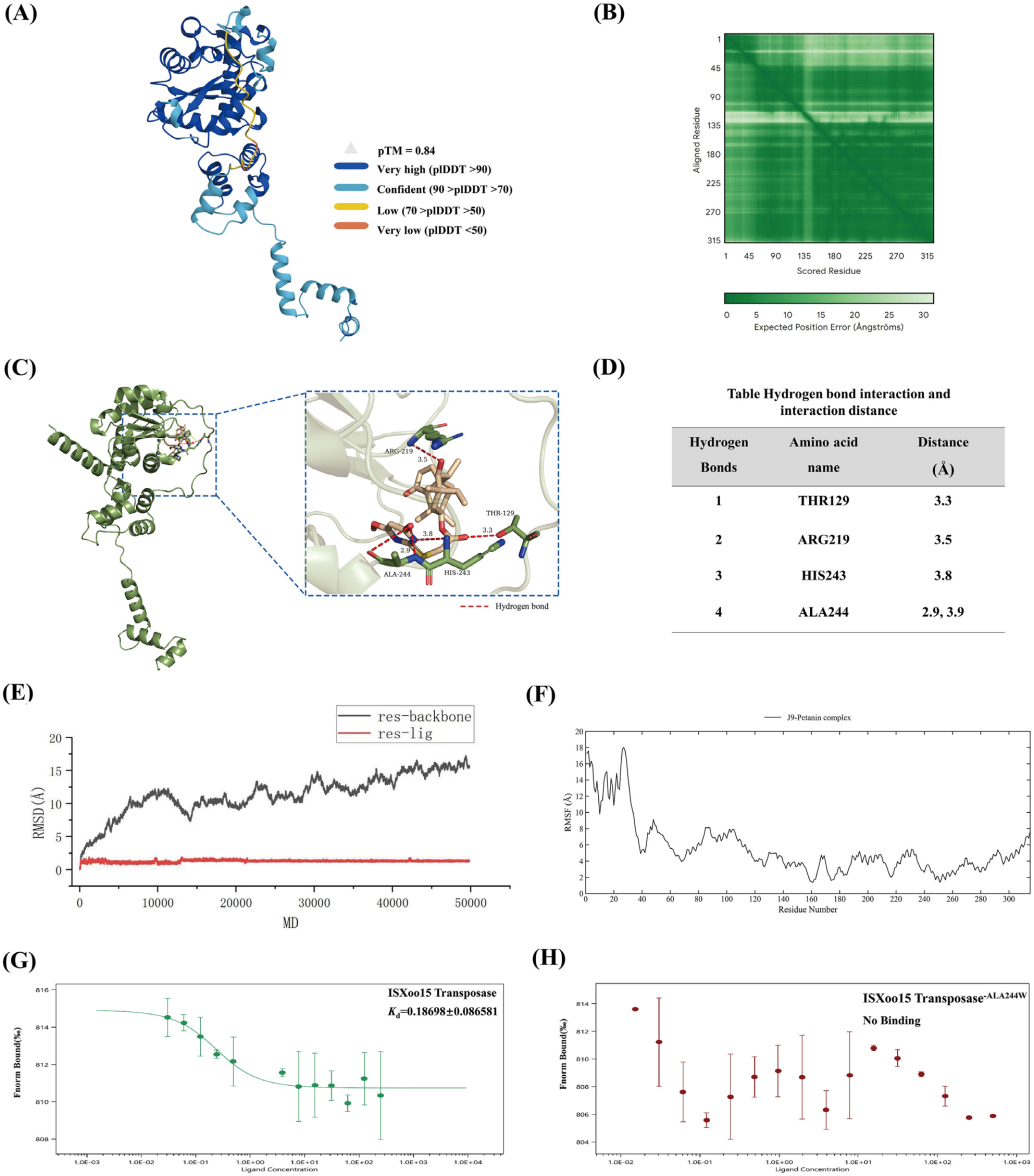
Computational and experimental validation of the interaction between J9 and IS*Xoo*15 transposase. (A) Predicted 3D structure of IS*Xoo*15 transposase generated by AlphaFold based on the amino acid sequence retrieved from UniProt. (B) Molecular docking of J9 with IS*Xoo*15 transposase using AutoDock Vina. The docking yielded a binding free energy of −6.8 kcal/mol. (C) Detailed view of hydrogen bond interactions between J9 and residues Thr129, Arg219, His243, and Ala244 of IS*Xoo*15 transposase, with Ala244 forming two distinct hydrogen bonds. (D) 2D interaction diagram illustrating hydrogen bond distances (Thr129, 3.3 Å; Arg219, 3.5 Å; Ala244, 2.9 Å). (E) Root‐mean‐square deviation (RMSD) trajectories of the J9‐IS*Xoo*15 transposase complex over a 50‐ns molecular dynamics (MD) simulation, showing structural stability within 0.44–1.64 Å for the protein backbone and 11.4–15.7 Å for the ligand. (F) Root‐mean‐square fluctuation (RMSF) analysis of IS*Xoo*15 transposase residues during MD simulation. Residues Thr129, Arg219, His243, and Ala244 in the ligand‐binding pocket displayed low fluctuations, whereas the N‐terminal region showed increased flexibility. (G) Microscale thermophoresis (MST) analysis of the binding affinity between J9 and recombinant wild‐type IS*Xoo*15 transposase, yielding a dissociation constant (*K*_d_) of 0.187 μM. (H) MST analysis of the A244W mutant showing nearly abolished binding to J9, confirming the critical role of Ala244 in ligand recognition.

To further investigate the interaction between IS*Xoo*15 transposase and J9, the amino acid sequence of IS*Xoo*15 transposase was retrieved from UniProt (https://www.uniprot.org/) (sequence data provided in Supporting Information). Homology modelling of the target protein was performed using the AlphaFold Server (https://alphafoldserver.com/) based on the obtained sequence (Figure 3A,B). The resulting modelled protein structure was subjected to molecular docking with the highly active small molecule J9 using the open‐source software AutoDock Vina. The docking analysis yielded a binding free energy of −6.8 kcal/mol, indicative of strong intermolecular interactions. The conformation with the highest binding affinity was extracted for further analysis. Key hydrogen bond interactions were identified between J9 and residues Thr129, Arg219, His243 and Ala244 (A244) of IS*Xoo*15 transposase, with A244 forming two distinct hydrogen bonds (Figure 3C,D). Among these interactions, the distances for Thr129 (3.3 Å), Arg219 (3.5 Å), and one of the A244 hydrogen bonds (2.9 Å) were all < 3.5 Å, suggesting stable binding between J9 and the target protein (Figure 3D). To validate the dynamic stability of the J9‐IS*Xoo*15 transposase complex, molecular dynamics simulations were conducted over a 50‐ns trajectory. The root‐means‐square‐deviation (RMSD) of the IS*Xoo*15 transposase backbone remained within 0.44–1.64 Å. After exceeding 30 ns, the RMSD of J9 fluctuated between 11.4 Å and 15.7 Å, with deviations not exceeding 4.3 Å (Figure 3E). To further probe the dynamic stability of the protein–small molecule complex, residue‐specific root‐mean‐square fluctuation (RMSF) analysis was performed on the system. The results indicated that RMSF values for the majority of residues fell within the 2–8 Å range, reflecting the overall structural integrity of the complex. Notably elevated fluctuations were exclusively observed in the N‐terminal region, where the first 40 residues exhibited RMSF values approaching 18 Å, suggesting substantial conformational flexibility in this segment (Figure 3F). In contrast, key ligand‐binding pocket residues Thr129, Arg219, His243 and Ala244, demonstrated significantly lower fluctuations. This pronounced rigidity signifies that ligand binding effectively constrains local conformational dynamics, thereby contributing to the stabilisation of the macromolecular complex. To experimentally verify binding, recombinant wild‐type IS*Xoo*15 transposase was expressed in *Escherichia coli* and purified (Figure S78). Microscale thermophoresis (MST) analysis confirmed a strong binding affinity between J9 and WT IS*Xoo*15 transposase, with a dissociation constant (*K*_d_) of 0.187 μM (Figure 3G). We further selected the residue Ala244, which formed the shortest hydrogen bond with J9 in the docking results, for binding contribution validation. By substituting the alanine at residue 244 with tryptophan, we constructed the mutated IS*Xoo*15 transposase^A244W^ (Figure S79). The MST results demonstrated that the mutant protein almost failed to bind to J9, indicating the critical importance of the Ala244 site in ensuring strong binding affinity between J9 and the IS*Xoo*15 transposase (Figure 3H). These computational and experimental results collectively suggest that J9 binds IS*Xoo*15 transposase with high affinity, forming a stable complex.

### 2.5 IS*Xoo*15 Transposase Is Evolutionarily Conserved in Xoo Strains

**FIGURE 4:**
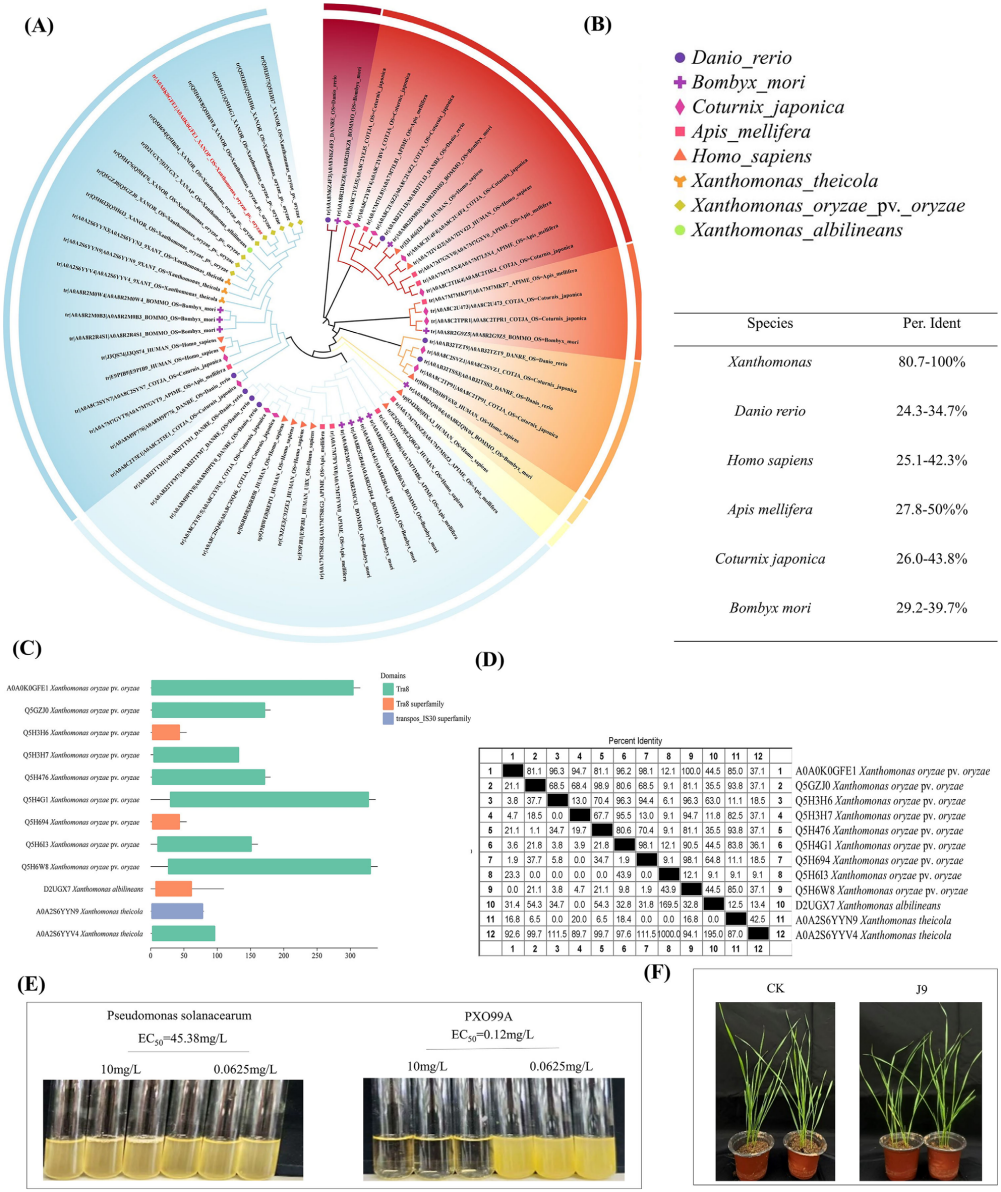
Target bioinformatics analysis and safety assessment of J9 small molecule. (A,B) Phylogenetic tree constructed based on homologous sequences of IS*Xoo*15 transposase across *Xanthomonas oryzae* pv. *oryzae* (Xoo) strains. Strong clustering of Xoo strains was observed, indicating evolutionary conservation of the IS*Xoo*15 transposase among pathogenic isolates. (C,D) Phylogenetic comparison of IS*Xoo*15 transposase homologues in non‐target organisms, including *Homo sapiens* , *Danio rerio* , and *Apis mellifera* , showing clear evolutionary divergence from Xoo. Domain‐based analysis of the *PXO_03433* gene revealed relatively conserved evolution within the *Xanthomonas* genus. (E) Evaluation of the antibacterial activity of compound J9 against *Pseudomonas solanacearum* using a turbidimetric assay. J9 exhibited markedly reduced activity against *P. solanacearum* (EC_50_ = 45.38 mg/L) compared with its potent activity toward Xoo PXO99A (EC_50_ = 0.12 mg/L). (F) Phytotoxicity assessment of J9 in wheat. No visible phytotoxic symptoms were observed even at a high application rate of 150 g a.i. ha^−1^, demonstrating good crop safety.

Sustainable agrochemicals should achieve broad‐spectrum efficacy against pathogens while ensuring minimal toxicity to non‐target organisms (Song et al. 2024). Building on the identification of IS*Xoo*15 transposase as a promising bactericidal target, we sought to investigate its evolutionary conservation across Xoo strains and sequence divergence in non‐target organisms. A phylogenetic tree based on IS*Xoo*15 transposase homologues (Figure 4A,B) was constructed. The analysis demonstrated strong clustering of Xoo strains, while non‐target organisms (e.g., humans, zebrafish, honeybees) exhibited distinct evolutionary divergence (Figure 4C,D). At the same time, the evolutionary conservation of the *PXO_03433* gene in the same genus was analysed from the perspective of structural domains, and the results showed that the *PXO_03433* gene evolved relatively conservatively in the Xoo species. Given this, to investigate whether compound J9 exhibits inhibitory activity against non‐target microorganisms and any phytotoxic effects, we evaluated its efficacy against *Pseudomonas solanacearum* using a turbidimetric assay (Figure 4E). The results revealed markedly inferior antibacterial activity of J9 against *P. solanacearum* (EC_50_ = 45.38 mg/L) stranded in contrast to its activity toward Xoo strain PXO99A (EC_50_ = 0.12 mg/L). Moreover, J9 showed favourable crop safety profiles. Even at a high concentration of 150 g a.i. ha^−1^, no significant visual phytotoxicity was observed in treated wheat plants (Figure 4F). These findings reinforce the target specificity of J9 and underscore the evolutionarily conserved role of the IS*Xoo*15 transposase in Xoo.

### 2.6 *PXO_03433* Knockout Impairs Xoo Pathogenesis‐Associated Traits and Virulence

**FIGURE 5:**
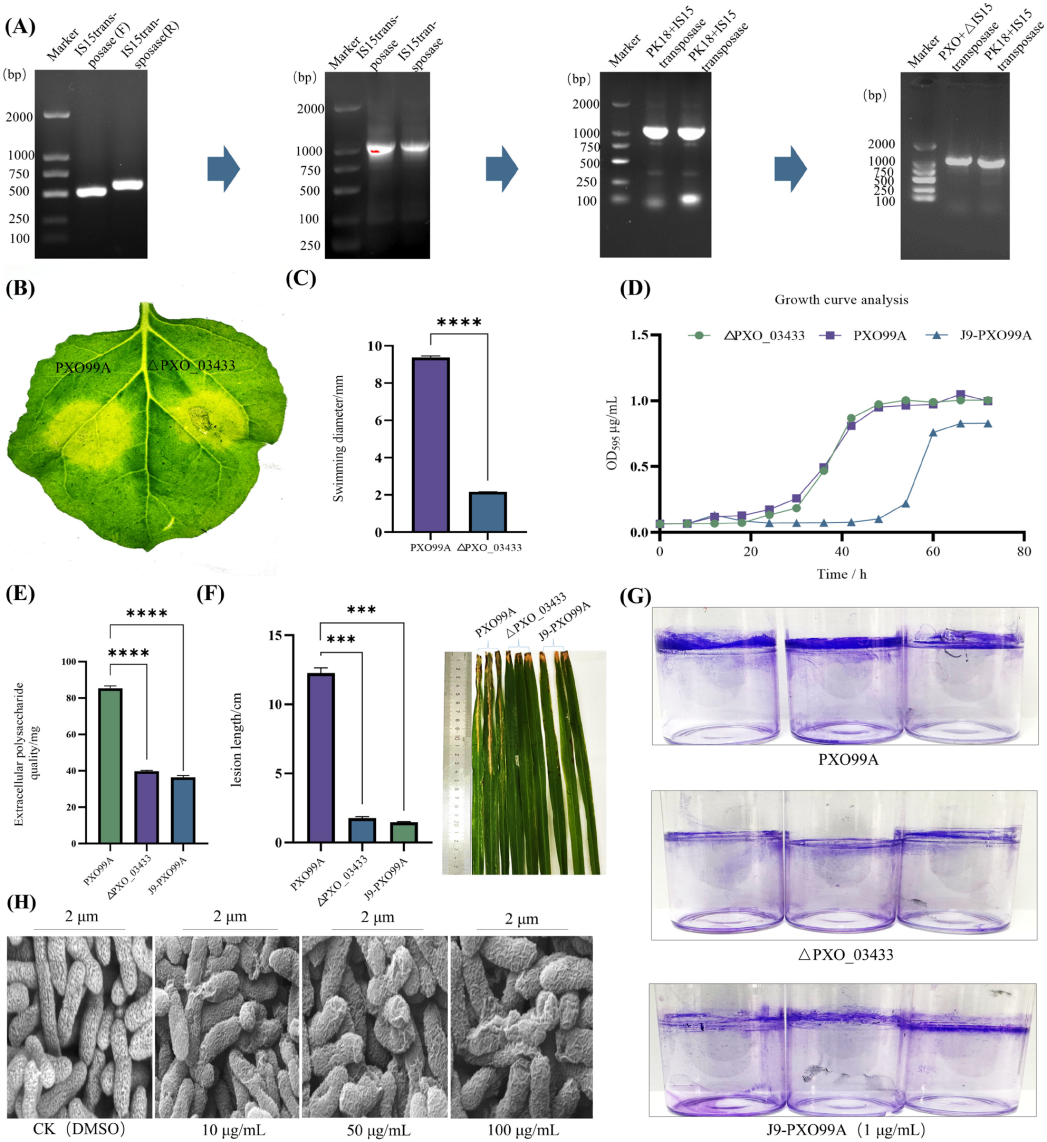
Construction of the mutant strain ΔPXO_03433, analysis of the physiological and biochemical changes of the ΔPXO_03433 mutant strain, and analysis of the influence of the J9 small molecule on the cell membrane structure. (A) Construction of mutant strains and physiological and biochemical analysis. Construction of *PXO_03433* gene deletion mutant strain. (B) Analysis of the impact of *PXO_03433* gene deletion on hypersensitivity response ability. 1 mL of each pretreated bacterial liquid was injected into the leaves of *Nicotiana benthamiana*. The appearance of spots was recorded on the leaves after 4 days. (C) Analysis of the impact of *PXO_03433* gene deletion on motility. The growth diffusion radius of the ΔPXO_03433 and PXO99A bacterial liquid (2 μL) without chemical treatment when cultured on nutrient agar medium containing 6% agar in a constant temperature incubator at 28°C for 5 days. (D) Analysis of growth curves for ΔPXO_03433, PXO99A, and J9‐PXO99A. The growth curves of PXO99A treated with 1 mg/L J9 small molecules and Δ PXO_03433 and PXO99A without chemical treatment. (E) Analysis of the effects of J9‐PXO99A and *PXO_03433* gene deletion on extracellular polysaccharides. The production of extracellular polysaccharides in the bacterial liquid of PXO99A treated with 1 mg/L J9 small molecules and ΔPXO_03433 and PXO99A without chemical treatment after 5 days of culture in a constant temperature shaker at 28°C. (F) Analysis of the effects of J9‐PXO99A and *PXO_03433* gene deletion on pathogenicity. The average lesion length 14 days after inoculation of PXO99A treated with 200 mg/L J9 small molecules and ΔPXO_03433 and PXO99A without drug treatment. (G) Analysis of the effect of *PXO_03433* gene deletion treated with J9 on the formation of biofilms. The biofilm formation of PXO99A treated with 1 mg/L J9 small molecule and Δ PXO_03433 and PXO99A without chemical treatment. (H) Effect of compound J9 on the cell morphology of *Xanthomonas oryzae* pv. *oryzae*. The morphological changes of PXO99A cells treated with 10, 50 and 100 mg/L J9 small molecule and PXO99A cells without drug treatment. Error bars indicate ± SD of the mean three independent experiments (*n* = 3 biological replicates). Statistical *p*‐values were calculated using the one‐tailed paired sample *t* tests (***p < 0.001; ****p < 0.0001).

Mounting evidence underscores the critical involvement of ISs in mediating pathogenicity and genetic plasticity within *Xanthomonas* species (Zhang, He, et al. 2024; Zhang, Zhang, et al. 2024; Oliveira et al. 2018; Xu et al. 2013; Lipszyc et al. 2022; Fernandes et al. 2024). Transposable elements (TEs) have an important role in gene regulation and are key genomic participants (Tossolini et al. 2025). To elucidate the functional contribution of IS*Xoo*15 transposase to Xoo virulence, we generated a *PXO_03433* gene knockout mutant ΔPXO_03433 (Figure 5A) through homologous recombination and conducted comparative analyses of pathophysiological parameters between ΔPXO_03433 and J9‐treated wild‐type strains. The results showed that deletion of the *PXO_03433* gene moderately attenuated the hypersensitive response (HR) capacity of Xoo PXO99A, suggesting its role in regulating effector molecules mediated by the type III secretion system (Figure 5B). *PXO_03433* knockout significantly impaired bacterial motility, with wild‐type PXO99A exhibiting colony diameters 4.47‐fold larger than those of the ΔPXO_03433 mutant under tested conditions (Figure 5C). The ΔPXO_03433 mutant exhibited a comparable growth rate to the wild‐type strain PXO99A, indicating that deletion of *PXO_03433* did not affect bacterial growth. In contrast, treatment of PXO99A with compound J9 resulted in a delayed entry into the logarithmic growth phase (Figure 5D). Additionally, the absence of the *PXO_03433* gene and J9 treatment reduced the extracellular polysaccharide (EPS) production in Xoo, with levels decreased to < 50% of wild‐type quantities (Figure 5E). We further conducted pathogenicity assays, and the results showed that wild‐type Xoo induced rice leaf lesions 6.9‐fold and 8.3‐fold longer than ΔPXO_03433 and J9‐applied rice (200 mg/L), respectively, indicating significantly reduced pathogenicity (Figure 5F). After staining the biofilm attached to the bottle wall with crystal violet, it was observed that both *PXO_03433* gene deletion and J9 treatment had inhibitory effects on the biofilm formation of PXO99A (Figure 5G). To investigate the impact of small‐molecule J9 on Xoo cell morphology, scanning electron microscopy revealed concentration‐dependent morphological aberrations in PXO99A cells. Treatment with 1–100 mg/L J9 induced progressive cell surface deformation, cytoplasmic contraction and membrane fragmentation, suggesting structural destabilisation of bacterial envelopes (Figure 5H). Taken together, these findings implicate that J9 has negative effects on EPS production, mobility, pathogenicity and biofilm formation of PXO99A. As the molecular receptor of J9, IS*Xoo*15 transposase serves as a multifunctional virulence determinant.

### 2.7 Complementation of *PXO_03433* Restores Xoo Pathological Indexes and Virulence

**FIGURE 6:**
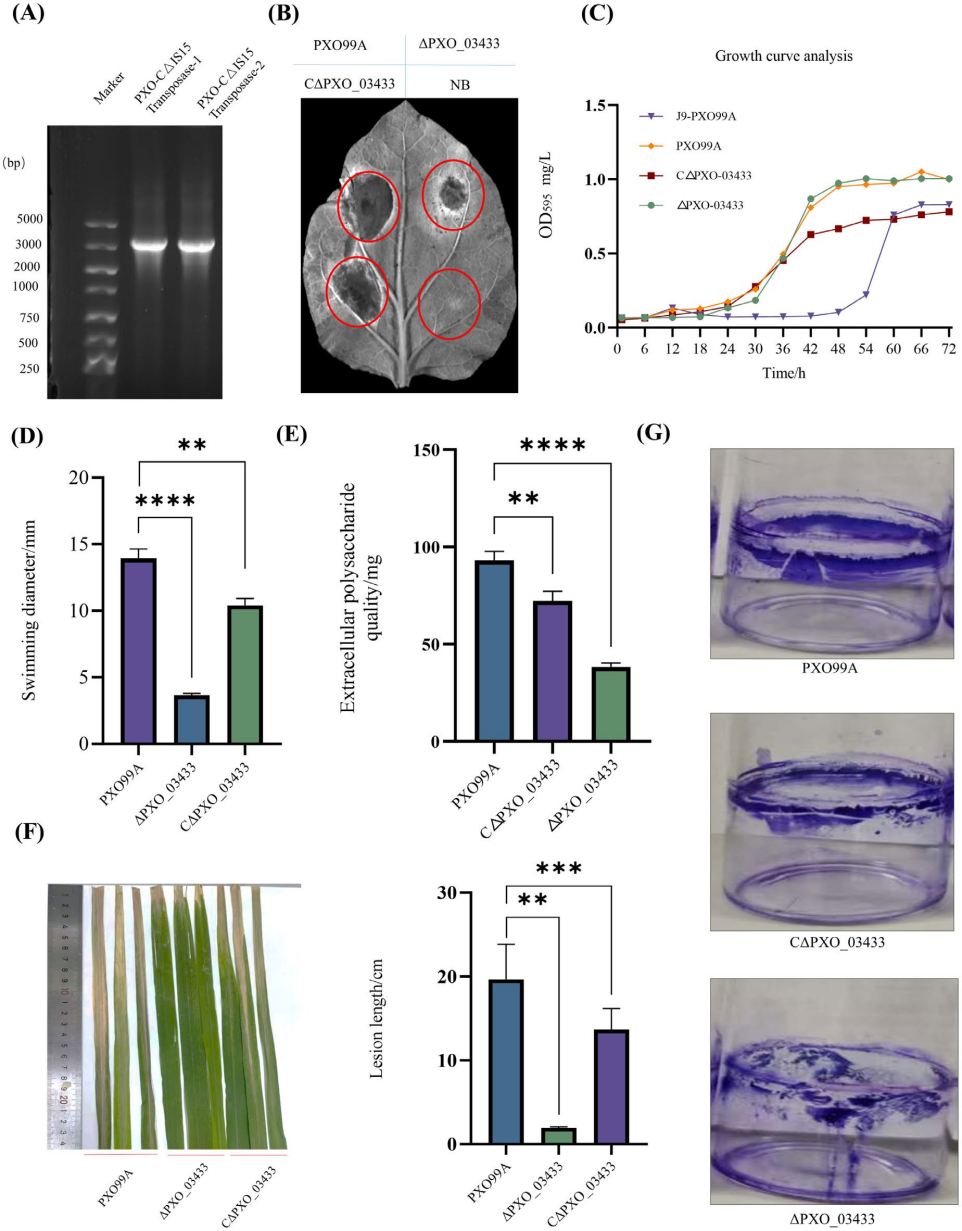
Construction of the complemented strain CΔPXO_03433 and analysis of the physiological and biochemical changes of the CΔPXO_03433 strain. (A) Confirmation of the construction of CΔPXO_03433 generated by reintroducing *PXO_03433* into the ΔPXO_03433 mutant through homologous recombination. (B) Hypersensitive response (HR) assay on *Nicotiana benthamiana* leaves showing restoration of HR and non‐host resistance by CΔPXO_03433, comparable to the wild‐type (WT) strain PXO99A. NB, nutrient broth. (C) Analysis of growth curves for ΔPXO_03433, CΔPXO_03433, PXO99A and J9‐PXO99A. The growth curves of PXO99A treated with 1 mg/L J9 small molecule and CΔPXO_03433, ΔPXO_03433 and PXO99A without chemical treatment. (D) Motility assay demonstrating partial recovery of swimming ability in CΔPXO_03433. Colony diameters of CΔPXO_03433 were 2.89‐fold larger than those of ΔPXO_03433 and 0.77‐fold of WT. The growth and diffusion radius of the strains ΔPXO_03433, CΔPXO_03433 and PXO99A (2 μL) cultured on nutrient agar medium containing 6% agar in an incubator at 28°C for 5 days without chemical treatment. (E) Analysis of the effects of J9 treatment and *PXO_03433* gene deficiency and replacement loss on extracellular polysaccharides. The production of extracellular polysaccharides in the bacterial liquid of ΔPXO_03433, CΔPXO_03433 and PXO99A after 5 days of cultivation in a shaker at 28°C without chemical treatment. (F) Pathogenicity analysis of the ΔPXO_03433 deletion mutant, the complemented strain CΔPXO_03433, and the J9‐treated group. The average lesion length 14 days after inoculation of the bacterial liquid of ΔPXO_03433, CΔPXO_03433 and PXO99A without drug treatment. (G) Effects of the ΔPXO_03433 deletion mutant and the complemented strain CΔPXO_03433 on biofilm formation. The biofilm formation of ΔPXO_03433, CΔPXO_03433 and PXO99A without chemical treatment. Error bars indicate ± SD of the mean from three independent experiments (*n* = 3 biological replicates). Statistical *p*‐values were calculated using one‐tailed paired samples *t* tests (**p < 0.01; ***p < 0.001; ****p < 0.0001).

To further confirm that the observed attenuation of Xoo pathogenicity was due specifically to the deletion of the IS*Xoo*15 transposase‐encoding gene rather than off‐target genomic variations, we reintroduced *PXO_03433* into the knockout strain to generate the complemented strain CΔPXO_03433 (Figure 6A). Physiological and biochemical characterisation of CΔPXO_03433 revealed substantial restoration of phenotypes impaired in the ΔPXO_03433 mutant. The complemented strain regained the ability to trigger a rapid HR and non‐host resistance in the non‐host plant *Nicotiana benthamiana* (Figure 6B). Furthermore, the ΔPXO_03433 mutant exhibited a growth rate comparable to that of the wild‐type strain PXO99A, indicating that deletion of *PXO_03433* did not affect bacterial growth. Treatment of PXO99A with J9 delayed entry into the logarithmic phase. The PXO_03433‐complemented strain constructed with the pBBR1MCS‐5 vector grew more slowly than the ΔPXO_03433 mutant (Figure 6C), likely due to the metabolic burden and potential overexpression associated with plasmid‐based complementation (Mi et al. 2016). Motility assays indicated significant recovery in CΔPXO_03433, with colony diameters measuring 2.89‐fold larger than the ΔPXO_03433 mutant and 0.77‐fold that of PXO99A (Figure 6D). Complementation with *PXO_03433* also enhanced EPS production; though reaching only 77% of WT levels, EPS yield in CΔPXO_03433 was 1.89‐fold that of ΔPXO_03433 (Figure 6E). Notably, CΔPXO_03433 exhibited significantly restored virulence. Rice leaf lesions caused by the complemented strain were 0.69‐fold and 7.0‐fold larger than those caused by the wild‐type and ΔPXO_03433 strains, respectively (Figure 6F). Crystal violet staining of surface‐adhered biofilms further showed that *PXO_03433* complementation positively impacted biofilm formation (Figure 6G). Overall, these findings indicate that reintroduction of the *PXO_03433* gene restored bacterial functions related to HR induction, EPS production, motility, pathogenicity and biofilm formation. This further supports the role of the IS*Xoo*15 transposase as a key multifunctional virulence factor.

### 2.8 IS*Xoo*15 Transposase as a Key Factor Governing Genomic Homeostasis in Xoo

**FIGURE 7:**
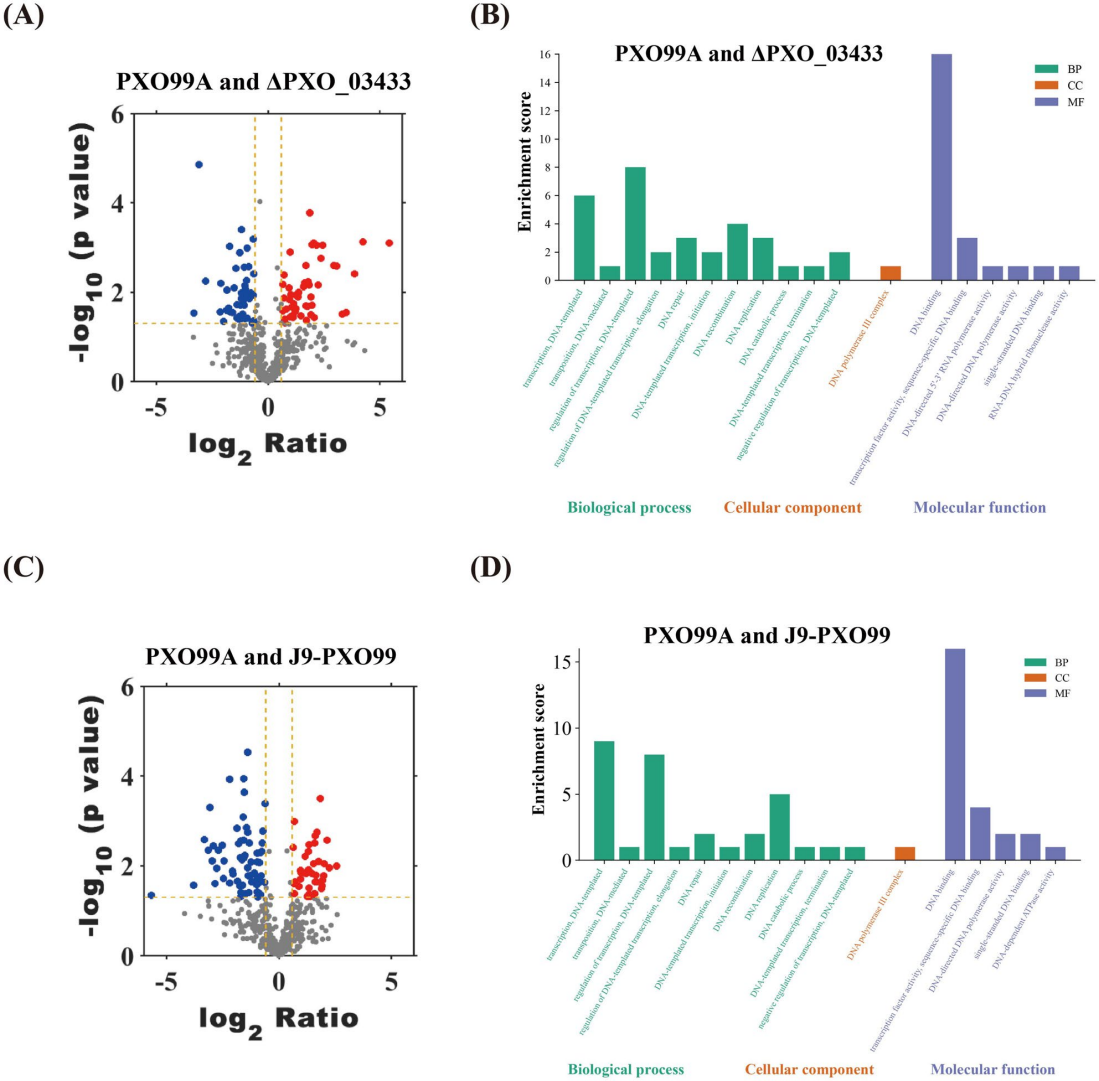
Proteomic profiling reveals DNA metabolic network perturbations in ΔPXO_03433 and J9‐treated *Xanthomonas oryzae* pv. *oryzae* strains. (A) Quantitative proteomic analysis of total proteins from wild‐type (PXO99A), J9‐treated PXO99A, and ΔPXO_03433 strains using label‐free iBAQ (intensity‐based absolute quantification) algorithm. A total of 733 proteins were identified, among which 158 were down‐regulated and 133 up‐regulated in the J9‐treated group, and 169 down‐regulated and 104 up‐regulated in the ΔPXO_03433 mutant compared with the wild type. (B, D) Gene Ontology (GO) enrichment analysis of differentially expressed proteins associated with DNA metabolic processes. Both ΔPXO_03433 (B) and J9‐treated (D) groups were significantly enriched in down‐regulated proteins related to DNA binding and DNA repair (*p* ≤ 0.05) including 10 and 12 DNA‐binding proteins, respectively, and 3 DNA repair‐related proteins. (C) Volcano plot illustrating global differential protein expression patterns in the J9‐treated and ΔPXO_03433 groups relative to the wild‐type strain.

TEs play a significant role in living organisms and can be found in all genomes. (Aziz et al. 2010) TEs are natural genomic remodelling and gene insertion vectors that can modify and transfer genetic information throughout the life tree (Michael and Querques 2024); that is, TEs are discrete DNA segments. They can be transferred from one location to another within their host genome through a process called translocation. TEs, together with other mobile genetic elements, form multiple dynamic groups and usually occupy the majority of genomic DNA in prokaryotes. (Ross et al. 2021) They have an indispensable interaction relationship with the metabolic network of DNA. To examine whether Δ*PXO_03433* has off‐target interactions or other pathways such as general stress responses being affected. The proteins in the PXO99A, J9‐PXO99A and Δ*PXO_03433* groups were quantitatively analysed. The iBAQ algorithm of protein spectrum label‐free technology was adopted to quantitatively simulate the total protein in each group. A total of 733 proteins of Xoo in rice were identified in the study. Among them, 158 were down‐regulated proteins and 133 were up‐regulated proteins in the chemical treatment group, while 169 were down‐regulated proteins and 104 were up‐regulated proteins in the mutation group (Figure 7A,C). We conducted GO (Gene Ontology) analysis on the proteins related to the DNA metabolic network and compared the differentially expressed proteins from the wild‐type group with those in the treatment group and the mutation group (*p* ≤ 0.05). Compared with the wild type, the ΔPXO_3433 group was significantly enriched with 10 down‐regulated proteins related to DNA binding and 3 down‐regulated proteins related to DNA repair at the molecular function and biological level (Figure 7B). The J9 treatment group was enriched with 12 down‐regulated proteins related to DNA binding and 3 down‐regulated proteins related to DNA repair (Figure 7D). Although the expression of IS*Xoo*15 transposase was not directly detected, the down‐regulated proteins in the DNA binding and repair‐related pathways were significantly enriched in both the transposase deletion mutant and the J9 treatment conditions, further indicating that both may affect DNA binding and repair functions. Thus, through a precise ‘knock‐out’ strategy, the profound impact of its functional loss on Xoo cells was revealed: IS*Xoo*15 transposase is an important influencing factor of genomic homeostasis in Xoo cells. Its existence constitutes a potential genotoxic pressure, forcing cells to maintain an efficient DNA monitoring and repair system. The absence of this transposase triggers a widespread stress response, specifically manifested as significant differential expression of a large number of DNA repair, recombination and binding proteins. These data indicate that the IS*Xoo*15 element is not a completely silent element, but actively interacts with host cells, and its activities shape the host's DNA metabolic network. It is worth noting that the J9‐treated wild‐type strain exhibited more extensive phenotypic impairments compared to the *PXO_03433* gene knockout mutant. This observation may suggest that J9 could additionally induce bacterial stress responses or secondary effects beyond solely inhibiting the target. Bactericides are known to elicit bacterial stress responses, such as the stringent response and oxidative stress, thereby activating protective mechanisms and potentially enhancing pathogen fitness (Poole 2012). However, excessively high levels of ROS and other damaging agents can ultimately lead to direct cellular mortality (Qi et al. 2024).

The discovery of novel targets holds significant promise for addressing the limitations of conventional bactericides including insufficient efficacy, evolving bacterial resistance and ecological risks. In summary, we determined lead compound J9 demonstrating exceptional in vitro and in vivo anti‐Xoo activity, and subsequently designed and synthesised its photoaffinity probe P‐J9. Using ABPP technology, we first identified IS*Xoo*15 transposase as the potential molecular target of J9. Molecular docking, dynamics simulations and affinity assay elucidated the binding mode between J9 and this enzyme. Physiological and biochemical analyses confirmed that knockout strains lacking the gene encoding this target exhibited significantly reduced pathogenicity and virulence, while these virulence‐associated phenotypes were substantially restored in the gene‐complemented strain. Proteomic profiling revealed consistent downregulation of pathways associated with DNA repair, recombination and DNA‐binding proteins in both J9‐treated and transposase‐deficient strains. This evidence suggested that IS*Xoo*15 transposase orchestrates critical regulatory functions in maintaining genomic metabolic homeostasis. Given the emerging recognition of TEs' critical role in pathogen genetic variation and virulence evolution, with transposases serving as essential drivers for transposon functionality, this study lays the groundwork for developing novel antimicrobial strategies targeting transposases to suppress phytobacterial diseases. Additionally, although the IS*Xoo*15 transposase has been confirmed as the primary in vivo receptor for J9 in Xoo, the potential secondary effects or off‐target consequences of J9 treatment on the strain cannot be overlooked and require further investigation in the future.

## 3 Experimental Procedures

All reagents used in the synthesis experiment were of analytical or chemical purity, purchased from commercial sources, and did not require further drying or purification when in use. The PXO99A strain used in biological experiments was from the laboratory of Guizhou University.

### 3.1 The General Procedure for Preparing the Target Compounds J1–J24

Synthesis of intermediates B and C was based on the literature (Ai et al. 2016). Detailed design and synthesis information for the title compounds J1–J24 is provided in Figure S1A,B.

### 3.2 In Vitro Antibacterial Activity

The antibacterial activities of the synthesised compounds (J1–J24) against Xoo were evaluated in vitro using the turbidity method described previously (Wang et al. 2013). TC and ZT were used as positive controls, and DMSO served as the blank control. Each compound and control was dissolved in DMSO and subsequently diluted to the desired experimental concentrations with 1 mL of water containing 0.1% Tween 80 and 4 mL of solvent medium. Approximately 50 μL of nutrient broth (NB) containing Xoo cells was added to each test tube. The bacterial suspensions were incubated at 28°C ± 1°C with shaking at 180 rpm for 24–48 h until the bacterial concentration in the blank control reached an optical density (OD_595_) value greater than 0.6. OD_595_ was measured using a Model 680 microplate reader (Bio‐Rad).

The inhibition rate (*I*) of each compound was calculated using the following formula (Li et al. 2014):

$$I\;\left(\%\right)=\left[\left(\mathrm{CK}-T\right)/\mathrm{CK}\right]\times 100$$

where CK represents the OD value of bacterial growth in the blank control, and *T* represents the OD value of bacterial growth in the treated group. All experiments were performed in triplicate. The 50% effective concentration (EC_50_) values were determined using GraphPad Prism v. 8.0.2 (GraphPad Software).

### 3.3 In Vivo Antibacterial Activities

Under greenhouse conditions, the preventive and curative activities of compound J9 against Xoo were evaluated using the leaf‐clipping method described previously (Li et al. 2018; Zhang et al. 2011). TC and ZT served as positive controls, while DMSO was used as a blank control. Compound J9 was dissolved in DMSO and diluted with 0.2% (vol/vol) Tween 80 aqueous solution to a final concentration of 200 mg/L. Rice plants at the tillering stage were grown in a greenhouse under natural light conditions at 28°C. The reagent was evenly sprayed onto the rice leaves until run‐off. After 24 h, the leaf tips (2–4 cm) were cut with scissors dipped in an Xoo suspension. A control treatment using water containing DMSO was included. More than 10 leaves were treated per replicate, and each treatment was repeated three times. Fourteen days after application, lesion lengths on rice leaves were measured, and the control efficacy was calculated based on disease severity.

For the curative assay, leaf tips were first cut with scissors dipped in the Xoo suspension. After 24 h, the 200 mg/L reagent was sprayed evenly onto the infected leaves until run‐off. Water containing DMSO was used as the control. Each treatment included more than 10 leaves and was repeated three times. Disease severity was assessed 14 days after treatment, and the control efficacy (*I*) was calculated as follows:

$$I\;\left(\%\right)=\left[\left(\mathrm{CK}-\mathrm{PT}\right)/\mathrm{CK}\right]\times 100$$

where CK and PT represent the lesion lengths of untreated and treated plants, respectively (Wang et al. 2019). All experimental plants were grown at 28°C ± 1°C with 70% relative humidity. Each experiment was performed with three biological replicates. Bioactivity data were calculated and analysed using SPSS v. 17.0 software.

### 3.4 The General Procedure for Preparing Probe P‐J9

The detailed synthesis of probe P‐J9 is provided in Supporting Information.

### 3.5 ABPP Assay

The ABPP is a powerful technique for labelling and detecting active enzyme species in cell lysates, cells or whole animals (Verhelst et al. 2020). Following the method described in the literature (Zhang et al. 2022), the reaction system for preparing the P‐J9 probe was set up, followed by dark reaction, click reaction and inactivation reaction. Subsequently, a non‐staining gel was prepared and electrophoresed, with imaging performed using a Bio‐Rad imaging system. Subsequently, membrane transfer, milk blocking and antibody incubation were performed. Finally, PVDF membranes were scanned and imaged using the ChemiDoc XRS+ system (Bio‐Rad). Concentration labelling, concentration dependence, and competitive experiments with the probe and J9 small molecule were conducted by adjusting their respective concentrations.

### 3.6 Pull‐Down Assay

Based on previous literature, the reaction system for the P‐J9 probe was prepared and the click reaction was performed. 1 mL of magnetic beads was activated separately with 1 mL of phosphate‐buffered saline (PBS) + 2 mM urea. The prepared reaction system was added to the tube containing the magnetic beads. The tube was wrapped with aluminium foil and placed on a rotating incubator for 14 h of enrichment in the dark. After enrichment, the centrifuge tube was placed on a magnetic stand and the supernatant was removed. Three washes were performed with 0.5 mM PBS (containing 2 mM urea) and mass spectrometry‐grade water. 100 μL of the final supernatant and the final wash liquid from the magnetic bead wash was collected for inactivation reactions. A non‐staining gel was prepared and electrophoresis was performed. The gel was imaged using a Bio‐Rad imaging system. Subsequently, membrane transfer, milk blocking, antibody incubation were performed, and the PVDF membrane was finally scanned using the ChemiDoc XRS+ system (Bio‐Rad) for imaging. To determine whether enrichment by magnetic beads occurred, 100 μL of iodacetamide was added to the system after removing the supernatant and washing. This was mixed thoroughly and incubated for 30 min (25°C, 900 rpm). Magnetic beads were washed three times with 0.5 mM PBS (containing 2 mM urea) and mass spectrometry water. 30 μL mass spectrometry water was added to the magnetic bead system, followed by 4 μL trypsin and 16 μL trypsin buffer for enzymatic digestion (37°C, 900 rpm) for 17 h. 10 μL of 0.1% formic acid was added to the digested mixture to terminate the reaction. The mixture was transferred to a 10 kDa ultrafiltration tube, centrifuged (13,400 *g*, 4°C, 40 min), and washed twice with 100 μL of mass spectrometry water. The filtrate was transferred to a centrifuge tube and freeze‐dried using a freeze dryer for mass spectrometry identification.

### 3.7 Protein Binding Capacity

IS *Xoo*15 transposase and the Ala244 mutant IS*Xoo*15 Transposase^A244W^ were provided by Wuhan JinKaiRui Biotechnology Co. Ltd. MST technology was employed to analyse interactions between J9 and biomolecules.

### 3.8 RT‐qPCR Assay

Total RNA was isolated from Xoo using the Bacteria Total RNA Isolation Kit (Sangon Biotech), and a cDNA synthesis kit (Vazyme Biotech) was used to conduct reverse transcription experiments. qPCR was performed using CFX real‐time PCR (Bio‐Rad) and TBGreen Fast qPCR mixture (Takara). Data were calculated using the $2^{-∆∆C_{T}}$ method (Zhuo et al. 2017). Each experiment was performed with three biological and technical replicates.

### 3.9 Bioinformatics Analysis

The amino acid sequence of the IS*Xoo*15 transposase was retrieved from UniProt (https://www.uniprot.org/) (sequence data provided in Supporting Information). Homology modelling of the target protein was performed using the AlphaFold Server (https://alphafoldserver.com/) based on the obtained sequence. Molecular docking between the simulated protein structure and the highly active small molecule J9 was conducted using the open‐source software AutoDock Vina. RMSD and RMSF experiments were performed using Amber software to validate the dynamic stability of the J9‐IS*Xoo*15 transposase complex. The corresponding protein sequences in the UniProt database were identified by BLAST searches, and then sequence alignment was performed using Mage software. Based on the sequence comparison results, phylogenetic evolutionary trees and domain visualisation data graphs were constructed.

### 3.10 Construction of the Mutant Strain ΔPXO_03433 and the Complementation Strain CΔPXO_03433

To construct ΔPXO_03433 and the complemented CΔPXO_03433 strains, previously described methods were followed (Wu et al. 2017). Wild‐type PXO99A was used as the parental strain, and allelic homologous recombination was performed using the suicide vector pK18mobsacB. Two flanking regions of *PXO_03433* were amplified using primers 1F‐1R and 2F‐2R (1F‐XbaI: 5′‐CTTGCATGCCTGCAGGTCGACATGCGCAGCGGCTGCAATTA‐3′; 1R‐SmaI: 5′‐TCAATCGCAATGCGTGCACAGGCGCTGGAAGAAGAGAT‐3′; 2F‐XbaI: 5′‐CTCTTCTTCCAGCGCCTGTGCACGCATTGCGATTGAGT‐3′; 2R‐SmaI: 5′‐AATTCGAGCTCGGTACCCGGGCGTCCAATCCATCTGGTGCT‐3′). The *PXO_03433* gene and the amplified arms were digested and ligated into pK18mobsacB using the corresponding restriction enzymes. The recombinant plasmid was introduced into PXO99A competent cells via electroporation. Primary transformants were selected on nutrient agar without sucrose (NAWS) plates containing 50 mg/L kanamycin, followed by secondary screening on nutrient agar (NA) plates with 15% sucrose. After two rounds of recombination, the *PXO_03433* knockout mutant was obtained.

To construct the complemented strain CΔPXO_03433, the ΔPXO_03433 mutant was used as the parental strain, and allelic homologous recombination was performed using the broad‐host‐range vector pBBR1MCS‐5 (Kovach et al. 1995; Obranić et al. 2013). The *PXO_03433* gene was amplified with primers 1F‐BamHI (5′‐CGCTCTAGAACTAGTGGATCCCGAAGTTTTCAACCGCTTCA‐3′) and 1R‐HindIII (5′‐GTCGACGGTATCGATAAGCTTCAAATGTCGGTGTCTGCTGT‐3′), digested, and ligated into pBBR1MCS‐5. The recombinant plasmid was introduced into ΔPXO_03433 competent cells by electroporation, and transformants were selected on NA plates containing 50 mg/L gentamicin. Cells carrying the CΔPXO_03433 construct were obtained after a single round of recombination. XbaI, SmaI, BamHI and HindIII restriction enzymes were purchased from New England Biolabs.

### 3.11 Growth of the ΔPXO_03433 and CΔPXO_03433 strains

Cultures were grown to the logarithmic phase (OD_595_ = 0.6). The culture was then diluted with sterile NB to an OD_595_ of 0.2 (J9 concentration in the J9‐PXO99A group was 1.0 mg/L). Aliquots of the diluted culture were added to 200 mL NB medium and incubated at 28°C with shaking at 180 rpm for 72 h. OD_595_ was measured every 6 h, with three independent replicates. Growth curves were plotted based on optical density and incubation time.

### 3.12 Motility, HR and Biofilm, Extracellular Polysaccharides Assays

#### 3.12.1 Motility Assay

Culture bacterial suspension was grown to OD_595_ = 0.6, adjusted to OD_595_ = 0.2 with NB medium, then 2 μL was dispensed onto NA medium containing 6% agar. Plates were incubated at 28°C for 5 days, the diffusion area of each strain's replicates was recorded.

#### 3.12.2 HR Assay

PXO099A and ΔPXO_03433 were transferred from NA medium to NB medium and incubated at 28°C, 180 rpm until the logarithmic phase (OD_595_ = 0.600). Using sterile syringes, 1 mL of logarithmic‐phase culture was injected into *N. benthamiana* leaves and incubated at room temperature for 2 days. This was performed with three replicates. Leaf spot formation was recorded after 4 days.

#### 3.12.3 Biofilm Assay

The crystal violet staining method was adopted. The bacterial suspension that had been cultivated to the logarithmic growth phase was adjusted to OD_595_ = 0.2 using sterile NB medium. 20 mL of the suspension was transferred to a 30 mL glass bottle and this process was repeated three times, where the J9 concentration of J9‐PXO99A is 1 mg/L. After culturing in an incubator at 28°C for 3–5 days, the excess suspended cells were rinsed off with sterile water, then 20 mL of 0.1% crystal violet solution was added until the biofilm stained purple. The results were recorded.

#### 3.12.4 Extracellular Polysaccharide Assays

The bacterial suspension was cultured to an OD_595_ value of 0.6, then diluted to 0.2 using the medium. 20 μL was pipetted into a conical flask containing 20 mL NB medium then incubated at 28°C in a shaking incubator for 5 days. The supernatant was centrifuged, then 3 volumes of ethanol added to the supernatant. This was allowed to stand for 12 h, then centrifuged, dried and weighed.

### 3.13 Pathogenicity Assay

Pathogenicity was evaluated as previously described (Wang et al. 2013). The tillering rice leaves were inoculated with a bacterial suspension of OD_595_ = 0.6. Each biological experiment was repeated three times. The length of the lesions was measured on the inoculated leaves 14 days later.

All experiments were performed with three biological replicates.

### 3.14 Proteomics Analysis

The wild‐type strain PXO99A and the mutant strain ΔPXO_03433 were cultured in NB to the logarithmic growth phase. The OD_595_ of each culture was adjusted to the same level, and 1% of the bacterial suspension was inoculated into fresh NB medium. Two parallel cultures of PXO99A were prepared. When the OD_595_ reached 0.2, one PXO99A culture was treated with compound J9 (1 mg/L), while the remaining PXO99A and ΔPXO_03433 cultures were treated with an equal volume of DMSO as the solvent control. Cultures were incubated with shaking until the OD_595_ of PXO99A reached 0.6. Cells were collected by centrifugation at 4700 *g* for 20 min, and the pellets were resuspended and disrupted by sonication. The supernatants were recovered and lyophilised to obtain crude protein extracts. Protein purification and quantification were performed as previously described (Yang et al. 2017).

Protein identification and quantification were conducted using a high‐resolution LC–MS/MS system. The Xoo proteome database was retrieved from UniProt (https://www.uniprot.org/). Raw MS data were analysed using MaxQuant software for peptide and protein identification, and label‐free quantification was performed based on the intensity‐based absolute quantification (iBAQ) algorithm integrated in MaxQuant. Differentially expressed proteins were subjected to Gene Ontology (GO) annotation and Kyoto Encyclopedia of Genes and Genomes (KEGG) pathway enrichment analyses to determine their biological functions and associated metabolic pathways.

## Author Contributions

**Funeng Lu:** writing – original draft, visualisation, validation, resources, methodology, investigation, formal analysis, data curation. **Ting Liu:** methodology, investigation. **Tangbing Yang:** formal analysis, software. **Ziming Wang:** formal analysis, software. **Jianzhuan Li:** formal analysis. **Chunni Zhao:** formal analysis. **Huan Wu:** software. **Deyu Hu:** writing – review and editing, supervision, funding acquisition, project administration. **Baoan Song:** writing – review and editing, supervision, project administration, conceptualization.

## Conflicts of Interest

The authors declare no conflicts of interest.

## Supporting information


**Figure S1:** Design and synthesis of compound **J**.
**Figure S2:** Synthetic routes of compound P‐J9.
**Figures S3–S77:** ^1^H NMR, ^13^C NMR and HRMS characterisation of compounds **J1–J24**.
**Figure S78:** SDS‐PAGE analysis of purified IS*Xoo*15 transposase.
**Figure S79:** SDS‐PAGE analysis of purified IS*Xoo*15 transposase^‐A244W^.
**Table S1:** EC_50_ values of title compounds against *Xoo*.
**Table S2:** Protective and curative activities of compound J9 against bacterial leaf blight 14 days after spraying.

## Data Availability

All data are contained within the manuscript, figures and Supporting Information.
