# Supplementary material for: Interfering Transposable Elements: IS Xoo 15 Transposase as a First‐in‐Class Antibacterial Target Against Xanthomonas oryzae pv. oryzae
Source: Mol Plant Pathol. 2025 Nov 16;26(11):e70169. doi: 10.1111/mpp.70169 (PMC12620416; doi:10.1111/mpp.70169)
Supplement: Supplementary file 1 — Figure S1: Design and synthesis of compound J. Figure S2: Synthetic routes of compound P‐J9. Figures S3–S77: 1H NMR, 13C NMR and HRMS characterisation of compounds J1–J24. Figure S78: SDS‐PAGE analysis of purified ISXoo15 transposase. Figure S79: SDS‐PAGE analysis of purified ISXoo15 transposase‐A244W. Table S1: EC50 values of title compounds against Xoo. Table S2: Protective and curative activities of compound J9 against bacterial leaf blight 14 days after spraying. [file MPP-26-e70169-s001.pdf]

Supporting Information

Interfering Transposable Elements: ISXoo15 Transposase as a First-in-Class Antibacterial Target Against *Xanthomonas oryzae* pv. *oryzae*

Funeng Lu[a,1], Ting Liu[a,1], Tangbing Yang[1], Ziming Wang[1], Jianzhuan Li[1], Chunni Zhao[1], Huan Wu[1], Deyu Hu[1,*], Baoan Song[1,*]

[1]State Key Laboratory of Green Pesticide, Center for R&D of Fine Chemicals of Guizhou University, Guiyang 550025, P. R. China.

[a]These authors contributed equally

*Corresponding author (Tel.: 86-851-88292148; Fax: 86-851-88292170; E-mail: dyhu@gzu.edu.cn; basong@gzu.edu.cn)

Contents

I. Design and synthesis steps of target compounds P-J9 and J1-J24 3

II. Tables S1 to S2 5

III. Characterization of Target Products (P-J9, J1-J24) 7

IV. ^{1}H, ^{13}C NMR, and HRMS of compound P-J9, J1-J24 data 26

V. Protein purification 51

I. Design and synthesis steps of target compounds P-J9 and J1-J24.

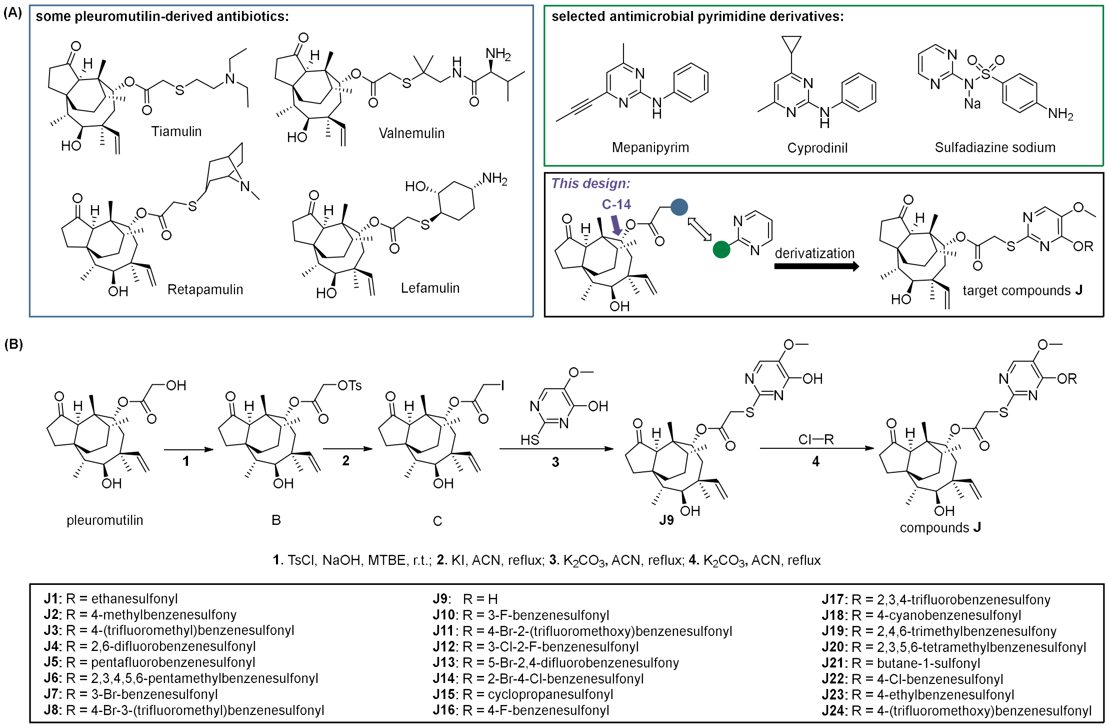

Figure S1. Design and synthesis of Compound **J**. (A) Design of Compound **J**. (B) Synthetic routes of Compounds **J1–J24**.

Synthesis of intermediate B:

Pleuromutilin (50.0 g, 129.5 mmol) and p-toluenesulfonyl chloride (27.4 g, 142.4 mmol) were mixed and stirred with 50 mL of water and 200 mL of methyl tert-butyl ether, NaOH (12.9 g, 323.6 mmol) aqueous solution was slowly added dropwise at room temperature, white turbidity was presented after dropping, and the temperature was refluxed, and the developing agent was ethyl acetate (v): petroleum ether (v) = 3:1, and the color was developed with potassium permanganate, and the reaction droplets were added to ice water after the reaction was completed, and the temperature was lowered to Filtered at 0-5 °C and washed with ice water and glacial methyl tert-butyl ether, the white solid intermediate B 61.9 g in yield 88.1%.

Synthesis of intermediate C:

Intermediate B (500 mg) is stirred and dissolved with 35 mL of ACN, and then 2.5 equivalent amounts of KI (389.5 mg) are added, after B is completely dissolved, the temperature is raised (78 °C) and refluxed for two hours, the developing agent is ethyl acetate (v): petroleum ether (v) = 3:1, after the reaction, it is extracted with dichloromethane, the organic layer is spun dry, and the intermediate C 408 mg in yield 88.9%.

Synthesis of J1-J24 of target compounds:

The intermediate C (500 mg) is stirred and dissolved with 35 mL of ACN, and after C is completely dissolved, then 1.8 equivalent K_2CO_3 (254.67 mg) is added, the temperature is raised (78 °C) and stirred for 30 min, 2 mercapto-5-methoxypyrimidine-4-ol is added, refluxed at 78 °C for two hours, the developing agent is ethyl acetate (v): petroleum ether (v) = 2:1, after the reaction, it is extracted with dichloromethane, the organic layer is spun dry, and the chromatography column is treated. The yield of the target compound J9 (446.0 mg) was obtained at 84%, J9 (300 mg) was

stirred and dissolved with 35 mL of ACN, and after J9 was completely dissolved, then 1.8 equivalent K_2CO_3 (254.67 mg) was added, the temperature was raised (78 °C) and stirred for 30 min, 1 equivalent amount of X (sulfonyl chloride with different groups) was added, and the reflux was 78 °C for 4 hours, and the developing agent was ethyl acetate (v): petroleum ether (v) = 2:1 (or 1:1), after the reaction, Extraction was carried out with methylene chloride, the organic layer was spun dry, and other target compounds were obtained after chromatography column chromatography treatment with a yield of 74-84%.

Synthesis of P-J9 probes

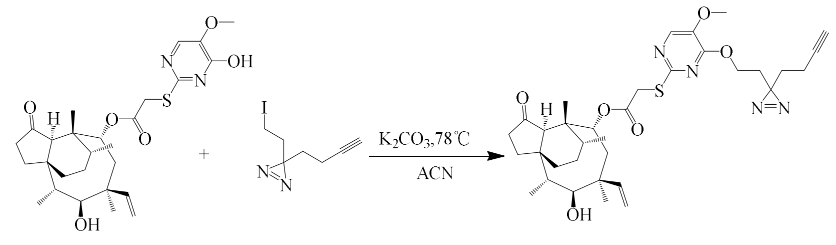

J9 G P-J9

Figure S2. Synthetic routes of Compound **P-J9**.

J9 (300 mg) is stirred and dissolved with 30 mL of ACN, and after J9 is completely dissolved, then 1.8 equivalent K_2CO_3 (254.7 mg) is added, and the temperature is raised (78 °C) and stirred for 30 min, 1 equivalent of G, refluxed at 78 °C for 4 hours, and the developing agent is ethyl acetate (v): petroleum ether (v) = 2:1 (or 1:1), after the reaction, The compound (J9) was 189.3 mg after extraction with dichloromethane, spin drying, and chromatography column chromatography treatment to obtain compound (J9) with a yield of 51.2%.

II. Tables S1 to S2

Table S1. EC_{50} Values of Title Compounds against *Xoo*.

No.	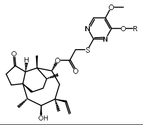 R	Toxic regression eqution	R^2	EC_{50}（mg/L）
J1	ethanesulfonyl	y=0.84x+5.20	R^2=0.90	0.41±0.15
J2	4-methylbenzenesulfonyl	y=0.66x+5.28	R^2=0.98	0.26±0.17
J3	4-(trifluoromethyl) benzenesulfonyl	y=0.63x+5.04	R^2=0.98	0.81±0.08
J4	2,6-difluorobenzenesulfonyl	y=0.81x+5.35	R^2=0.94	0.34±0.04
J5	Pentafluorobenzenesulfonyl	y=1.00x+5.38	R^2=0.99	0.77±0.56
J6	2,3,4,5,6-pentamethylbenzenesulfonyl	y=0.53x+5.30	R^2=0.90	0.33±0.09
J7	3-Br-benzenesulfonyl	y=0.59x+5.36	R^2=0.91	0.27±0.11
J8	4-Br-3-(trifluoromethyl) benzenesulfonyl	y=0.50x+5.39	R^2=0.91	0.23±0.15
J9	H	y=1.34x+6.36	R^2=0.94	0.12±0.02
J10	3-F-benzenesulfonyl chloride	y=0.49x+5.49	R^2=0.91	0.20±0.13
J11	4-Br-2-(trifluoromethoxy)benzenesulfonyl	y=0.51x+5.12	R^2=0.94	0.39±0.27
J12	3-Cl-2-F-benzenesulfonyl	y=0.78x+5.31	R^2=0.99	0.26±0.12
J13	5-Br-2,4-difluorobenzenesulfonyl	y=0.81x+5.53	R^2=0.91	0.22±0.05
J14	2-Br-4-Cl-benzenesulfonyl	y=1.40x+5.68	R^2=0.98	0.30±0.02
J15	cyclopropanesulfonyl	y=0.84x+5.29	R^2=0.92	0.37±0.09
J16	4-F-benzenesulfonyl	y=0.66x+5.36	R^2=0.93	0.26±0.03
J17	2,3,4-trifluorobenzenesulfonyl	y=0.60x+5.42	R^2=0.94	0.22±0.09
J18	4-cyanobenzenesulfonyl	y=1.01x+5.41	R^2=0.92	0.29±0.09
J19	2,4,6-trimethylbenzenesulfonyl	y=0.58x+5.44	R^2=0.94	0.16±0.06
J20	2,3,5,6-tetramethylbenzenesulfonyl	y=0.65x+5.33	R^2=0.93	0.35±0.06
J21	butane-1-sulfonyl	y= 0.39x+4.81	R^2=0.88	2.79±0.39
J22	4-Cl-benzenesulfonyl	y=0.73x+5.28	R^2=0.98	0.34±0.17
J23	4-ethylbenzenesulfonyl	y=0.81x+5.09	R^2=0.96	0.58±0.07
J24	4-(trifluoromethoxy)benzenesulfonyl	y=0.54x+5.36	R^2=0.89	0.26±0.05
ZT	/	y=3.39x+0.22	R^2=0.92	26.15±1.31
TC	/	y=2.06x+1.74	R^2=0.87	86.39±20.4

Table S2 Protective and curative activities of compound J9 against bacterial leaf blight 14 Days after spraying

Treatment (200mg/L)	Bacterial leaf blight [a]			
	Disease index [b]	protective activity (%)	Disease index [b]	curative activity (%)
J9	37.78	50.96±2.1a	37.04	55.55±2.1a
TC	41.48	46.16±2.0ab	42.59	48.89±2.0b
ZT	42.96	44.23±2.0b	38.89	50.66±2.0c
CK	77.04	/	83.33	/

[a] Values are mean ± SD; One-way analysis of variance was performed on the results, and different lowercase letters indicated signifiacnt differences between treatment groups ($P<0.05$).

[b] a composite measure of overall morbidity and severity.

III. Characterization of Target Products (P-J9, J1-J24)

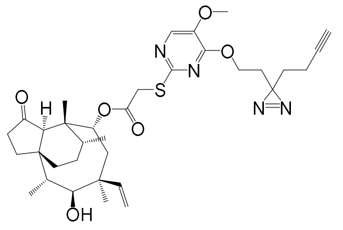

(3aR,4R,5R,7S,8S,9R,9aS,12R)-8-hydroxy-4,7,9,12-tetramethyl-3-oxo-7-vinyldecahydro-4,9a-propanocyclopenta[8]annulen-5-yl-2-((4-(2-(3-(but-3-yn-1-yl)-3H-diazirin-3-yl)ethoxy)-5-methoxypyrimidin-2-yl)thio)acetate (P-J9): Yield: 51.2%; green solid; m.p. 49.2-50.3 °C; ^{1}H NMR (400 MHz $CDCl_3$) δ 7.84 (s, 1 H), 6.48 (dd, J = 17.4, 11.0 Hz, 1 H), 5.76 (d, J = 8.4 Hz, 1 H), 5.30 (d, J = 11.0 Hz, 1 H), 5.18 (d, J = 17.4 Hz, 1 H), 4.40 – 4.20 (m, 2 H), 3.87 (s, 3 H), 3.79 (d, J = 5.3 Hz, 2 H), 3.35 (d, J = 6.4 Hz, 1 H), 2.30 (d, J = 6.5 Hz, 1 H), 2.27 – 2.15 (m, 2 H), 2.12 – 2.01 (m, 4 H), 2.00 (d, J = 2.4 Hz, 1 H), 1.90 (t, J = 6.5 Hz, 2 H), 1.76 (dd, J = 16.8, 4.8 Hz, 3 H), 1.70 – 1.58 (m, 3 H), 1.54 (d, J = 13.7 Hz, 1 H), 1.44 (s, 4 H), 1.36 (dd, J = 13.8, 3.1 Hz, 1 H), 1.28 (d, J = 16.1 Hz, 1 H), 1.14 (s, 4 H), 0.86 (d, J = 7.0 Hz, 3 H), 0.73 (d, J = 6.9 Hz, 3 H). ^{13}C NMR (126 MHz DMSO) δ 217.71, 167.95, 159.30, 158.84, 141.26, 139.86, 139.53, 115.74, 83.61, 73.08, 72.23, 70.25, 62.21, 57.73, 57.04, 36.85, 34.52, 32.17, 31.88, 30.60, 29.00, 27.3, 27.12, 24.97, 16.57, 15.01, 13.17, 12.05. HRMS (ESI): calculated for $C_{34}H_{46}O_6N_4NaS$ $[M+Na]^+$: 661.3030, found 661.3008.

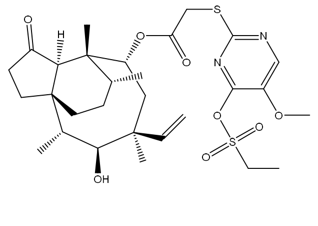

(3aR,4R,5R,7S,8S,9R,9aS,12R)-8-hydroxy-4,7,9,12-tetramethyl-3-oxo-7-vinyldecahydro-4,9a-propanocyclopenta[8]annulen-5-yl-2-((4-((ethylsulfonyl)oxy)-5-methoxypyrimidin-2-yl)thio)acetate (J1): Yield: 82.3%; Yellow solid; m.p. 112.4.-114.2 °C; ^{1}H NMR (600 MHz $CDCl_3$) δ 8.17(s, 1 H), 6.43 (dd, J = 17.3, 11.0 Hz, 1 H), 5.74 (d, J = 8.5 Hz, 1 H), 5.33 – 5.23 (m, 1 H),

5.19 – 5.13 (m, 1 H), 3.91(s, 3 H), 3.80 (d, *J* = 1.0 Hz, 2 H), 3.69 (q, *J* = 7.4 Hz, 2 H), 3.36 – 3.31 (m, 1 H), 2.30 – 2.27 (m, 1 H), 2.19 (ddt, *J* = 28.9, 19.4, 8.8 Hz, 2 H), 2.08(s, 1 H), 2.03 (dd, *J* = 16.0, 8.6 Hz, 1 H), 1.74 – 1.71 (m, 1 H), 1.63 (q, *J* = 11.2 Hz, 2 H), 1.56(S, 2 H), 1.52 (d, *J* = 3.2 Hz, 1 H), 1.50 – 1.43 (m, 2 H), 1.42(s, 4 H), 1.36 – 1.32 (m, 1 H), 1.29 (d, *J* = 16.1 Hz, 1 H), 1.14(s, 3H), 1.10 (dd, *J* = 14.1, 4.3 Hz, 1 H), 0.85 (d, *J* = 7.0 Hz, 3 H), 0.72 (d, *J* = 7.1 Hz, 3 H). ^{13}C NMR (151 MHz $CDCl_3$) δ 217.07, 167.64, 160.04, 153.57, 143.41, 141.54, 139.15, 117.14, 74.57, 69.94, 58.15, 57.16, 49.21, 45.48, 44.60, 43.95, 41.89, 36.76, 36.03, 34.65, 34.49, 30.43, 26.88, 26.44, 24.84, 16.77, 14.89, 11.51, 8.30. HRMS (ESI): calculated for $C_{29}H_{42}O_8N_2NaS_2$ $[M+Na]^+$: 633.2275, found 633.2253.

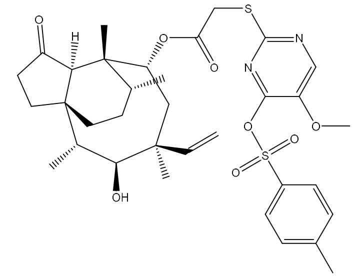

(3aR,4R,5R,7S,8S,9R,9aS,12R)-8-hydroxy-4,7,9,12-tetramethyl-3-oxo-7-vinyldecahydro-4,9a-propanocyclopenta[8]annulen-5-yl-2-((5-methoxy-4-(tosyloxy)pyrimidin-2-yl)thio)acetate (J2): Yield: 81.4%; white solid; m.p. 63.2-64.5 °C; ^{1}H NMR (600 MHz $CDCl_3$) δ 8.10(s, 1 H), 7.93 (d, *J* = 8.4 Hz, 2 H), 7.36 (d, *J* = 8.2 Hz, 2 H), 6.41 (dd, *J* = 17.4, 11.0 Hz, 1 H), 5.71 (d, *J* = 8.6 Hz, 1 H), 5.25 (dd, *J* = 11.0, 1.0 Hz, 1 H), 5.15 (dd, *J* = 17.3, 1.2 Hz, 1 H), 3.85(s, 3 H), 3.68 – 3.66 (m, 2 H), 3.32(s, 1 H), 2.45(s, 3 H), 2.30 – 2.25 (m, 1 H), 2.18 (tt, *J* = 19.4, 9.5 Hz, 2 H), 2.05(s, 1 H), 2.03 – 1.95 (m, 1 H), 1.74 – 1.70 (m, 1 H), 1.61 (td, *J* = 11.1, 9.1, 4.2 Hz, 2 H), 1.50 (td, *J* = 13.6, 2.9 Hz, 1 H), 1.47 – 1.39 (m, 2 H), 1.37(s, 3 H), 1.32 (dd, *J* = 14.2, 3.0 Hz, 1 H), 1.29 – 1.22 (m, 2 H), 1.12(s, 3 H), 0.83 (d, *J* = 7.0 Hz, 3 H), 0.67 (d, *J* = 7.1 Hz, 3 H). ^{13}C NMR (151 MHz $CDCl_3$) δ 217.11, 167.56, 159.87, 153.50, 145.96, 143.09, 141.13,

139.11, 133.73, 129.76, 129.16, 117.16, 74.60, 69.82, 58.17, 57.09, 53.50, 45.49, 44.56, 43.94, 41.88, 36.78, 36.03, 34.52, 30.45, 29.74, 26.88, 26.42, 24.86, 21.85, 16.75, 14.88, 11.51. HRMS (ESI): calculated for $C_{34}H_{44}O_8N_2NaS_2$ $[M+Na]^+$: 695.2431, found 695.2407.

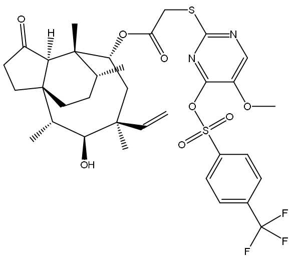

(3aR,4R,5R,7S,8S,9R,9aS,12R)-8-hydroxy-4,7,9,12-tetramethyl-3-oxo-7-vinyldecahydro-4,9a-propanocyclopenta[8]annulen-5-yl-2-((5-methoxy-4-(((4-(trifluoromethyl)phenyl)sulfonyl)oxy)pyrimidin-2-yl)thio)acetate (J3): Yield: 79.2%; white solid; m.p. 60.6-62.1°C; ^{1}H NMR (600 MHz $CDCl_3$) δ 8.21 (d, J = 8.3 Hz, 2 H), 8.14(s, 1 H), 7.86 (d, J = 8.4 Hz, 2 H), 6.42 (dd, J = 17.5, 11.0 Hz, 1 H), 5.72 (d, J = 8.5 Hz, 1 H), 5.26 (dd, J = 11.0, 1.1 Hz, 1 H), 5.15 (dd, J = 17.6, 1.3 Hz, 1 H), 3.87(s, 3 H), 3.68(s, 2 H), 3.32 (t, J = 7.8 Hz, 1 H), 2.29 – 2.25 (m, 1 H), 2.25 – 2.13 (m, 2 H), 2.06(s, 1 H), 2.00 (dd, J = 16.4, 8.9 Hz, 1 H), 1.73 (dq, J = 14.7, 3.2 Hz, 1 H), 1.64 – 1.59 (m, 2 H), 1.55 – 1.47 (m, 1 H), 1.45 – 1.39 (m, 2 H), 1.38(s, 3 H), 1.33 (dd, J = 14.4, 3.3 Hz, 1 H), 1.26 (d, J = 16.0 Hz, 1 H), 1.13(s, 3 H), 1.09 (dd, J = 14.1, 4.3 Hz, 1 H), 0.83 (d, J = 7.1 Hz, 3 H), 0.68 (d, J = 7.1 Hz, 3 H). ^{13}C NMR (151 MHz $CDCl_3$) δ 217.07, 167.48, 159.95, 153.11, 143.29, 141.05, 140.35, 139.13, 136.02, 129.73, 126.33, 123.95, 122.14, 117.18, 74.60, 69.94, 58.16, 57.08, 45.49, 44.60, 43.95, 41.89, 36.77, 36.05, 34.50, 30.45, 26.88, 26.42, 24.87, 16.75, 14.85, 11.51. HRMS (ESI): calculated for $C_{34}H_{41}O_8N_2F_3NaS_2$ $[M+Na]^+$: 749.2149, found 749.2125.

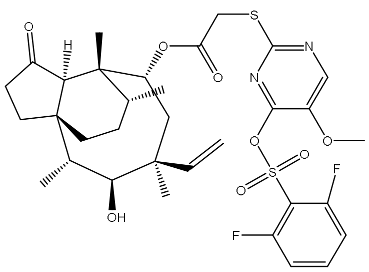

(3aR,4R,5R,7S,8S,9R,9aS,12R)-8-hydroxy-4,7,9,12-tetramethyl-3-oxo-7-vinyldecahydro-4,9a-propanocyclopenta[8]annulen-5-yl-2-((4-(((2,6-difluorophenyl)sulfonyl)oxy)-5-methoxypyrimidin-2-yl)thio)acetate (J4): Yield: 79%; white solid; m.p. 81.7-83.6 °C; ^{1}H NMR (600 MHz $CDCl_3$) δ 8.15(s, 1 H), 7.64 (ddd, J = 8.5, 5.9, 2.7 Hz, 1 H), 7.09 (t, J = 8.5 Hz, 2 H), 6.40 (dd, J = 17.4, 11.0 Hz, 1 H), 5.70 (d, J = 8.5 Hz, 1 H), 5.27 – 5.25 (m, 1 H), 5.18 – 5.11 (m, 1 H), 3.88(s, 3 H), 3.61(s, 2 H), 3.36 – 3.27 (m, 1 H), 2.28 – 2.24 (m, 1 H), 2.18 (ddt, J = 28.9, 19.4, 9.5 Hz, 2 H), 2.05(s, 1 H), 2.02 – 1.97 (m, 1 H), 1.72 (dd, J = 14.6, 2.7 Hz, 1 H), 1.64 – 1.58 (m, 2 H), 1.50 (dd, J = 13.9, 3.3 Hz, 1 H), 1.46 – 1.38 (m, 2 H), 1.3(s, 3 H), 1.32 (dd, J = 14.3, 3.0 Hz, 1 H), 1.25 (d, J = 16.1 Hz, 1 H), 1.13(s, 3 H), 1.08 (dd, J = 14.1, 4.3 Hz, 1 H), 0.83 (d, J = 7.0 Hz, 3 H), 0.66 (d, J = 7.0 Hz, 3 H). ^{13}C NMR (151 MHz $CDCl_3$) δ 217.13, 167.42, 160.73, 160.04, 158.97, 153.37, 143.70, 140.85, 139.16, 136.71, 117.19, 116.10, 113.38, 113.24, 74.62, 69.92, 58.19, 57.23, 53.55, 45.51, 44.62, 43.97, 41.91, 36.78, 36.07, 34.50, 30.47, 26.90, 26.47, 24.88, 16.76, 14.90, 11.54. HRMS (ESI): calculated for $C_{33}H_{40}O_8N_2F_2NaS_2$ $[M+Na]^+$: 717.2086, found 717.2059.

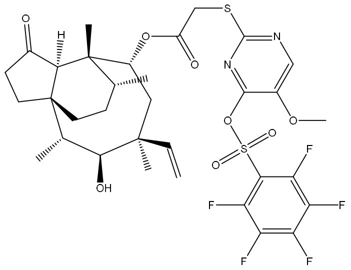

(3aR,4R,5R,7S,8S,9R,9aS,12R)-8-hydroxy-4,7,9,12-tetramethyl-3-oxo-7-vinyldecahydro-4,9a-propanocyclopenta[8]annulen-5-yl-2-((5-methoxy-4-

(((perfluorophenyl)sulfonyl)oxy)pyrimidin-2-yl)thio)acetate (J5): Yield: 81%; Orange solid; m.p. 88.2-89.7 °C; ^{1}H NMR (500 MHz CDCl$_3$) δ 8.19(s, 1 H), 6.41 (dd, *J* = 17.4, 11.0 Hz, 1 H), 5.72 (d, *J* = 8.5 Hz, 1 H), 5.29(d, 2 H), 5.16 (d, *J* = 17.4 Hz, 1 H), 3.92(s 3 H), 3.70(s, 2 H), 3.35 – 3.31 (m, 1 H), 2.27 (d, *J* = 7.2 Hz, 1 H), 2.19 (dq, *J* = 19.4, 10.4, 9.2 Hz, 1 H), 2.07(s, 1 H), 2.04 – 1.99 (m, 1 H), 1.79 – 1.71 (m, 1 H), 1.68 – 1.59 (m, 2 H), 1.54 – 1.48 (m, 1 H), 1.44 (dd, *J* = 8.9, 4.4 Hz, 2 H), 1.40(s, 3 H), 1.36 – 1.30 (m, 1 H), 1.26 (d, *J* = 16.1 Hz, 1 H), 1.14(s, 3 H), 1.10 (dd, *J* = 14.1, 4.3 Hz, 1 H), 0.85 (d, *J* = 7.0 Hz, 3 H), 0.68 (d, *J* = 7.0 Hz, 3 H). ^{13}C NMR (126 MHz DMSO) δ 217.67, 167.23, 159.23, 152.32, 146.03, 144.16, 141.25, 140.69, 115.61, 73.05, 70.59, 57.70, 55.43, 45.46, 44.45, 44.00, 41.99, 40.32, 40.21 – 39.77, 39.71, 39.66, 36.79, 34.39, 30.59, 28.94, 27.03, 24.96, 16.42, 14.75, 12.02. HRMS (ESI): calculated for $C_{33}H_{37}F_5O_8N_2NaS_2$ [M+Na]$^+$: 771.1804, found 771.1772.

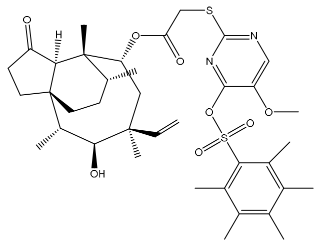

(3aR,4R,5R,7S,8S,9R,9aS,12R)-8-hydroxy-4,7,9,12-tetramethyl-3-oxo-7-vinyldecahydro-4,9a-propanocyclopenta[8]annulen-5-yl-2-((5-methoxy-4-(((2,3,4,5,6-pentamethylphenyl)sulfonyl)oxy)pyrimidin-2-yl)thio)acetate (J6): Yield: 78%; white solid; m.p. 89.6-90.6 °C; ^{1}H NMR (600 MHz CDCl$_3$) δ 8.05(s, 1 H), 6.41 (dd, *J* = 17.3, 11.0 Hz, 1 H), 5.68 (d, *J* = 8.5 Hz, 1 H), 5.29 – 5.26 (m, 1 H), 5.15 (dd, *J* = 17.3, 1.2 Hz, 1 H), 3.86(s, 3 H), 3.56 (d, *J* = 3.3 Hz, 2 H), 3.31(s, 1 H), 2.61(s, 6 H), 2.28(s, 3 H), 2.27 – 2.25 (m, 1 H), 2.24(s, 6 H), 2.17 (tt, *J* = 19.4, 9.4 Hz, 2 H), 2.05(s, 1 H), 2.02 – 1.96 (m, 1 H), 1.74 – 1.70 (m, 1 H), 1.65 – 1.57 (m, 2 H), 1.50 (dd, *J* = 13.9, 3.3 Hz, 1 H), 1.47 – 1.39 (m, 2 H), 1.34(s, 3 H), 1.33 – 1.29 (m, 1 H), 1.25

(d, J = 15.7 Hz, 1 H), 1.12(s, 3 H), 1.08 (dd, J = 14.0, 4.3 Hz,1 H), 0.83 (d, J = 7.0 Hz, 3 H), 0.64 (d, J = 7.0 Hz, 3 H). ^{13}C NMR (151 MHz $CDCl_3$) δ 217.09, 167.55, 159.70, 154.15, 142.65, 141.61, 140.56, 139.18, 135.30, 135.15, 134.60, 117.19, 74.62, 69.79, 60.48, 58.18, 57.17, 45.51, 44.66, 43.97, 41.91, 36.81, 36.05, 34.48, 30.48, 26.92, 26.48, 24.88, 18.95, 18.17, 17.23, 16.76, 14.84, 14.28, 11.53. HRMS (ESI): calculated for $C_{38}H_{52}O_8N_2NaS_2$ $[M+Na]^+$: 751.3057, found 751.3030.

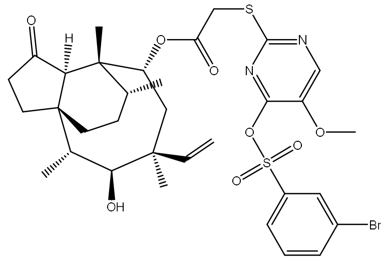

(3aR,4R,5R,7S,8S,9R,9aS,12R)-8-hydroxy-4,7,9,12-tetramethyl-3-oxo-7-vinyldecahydro-4,9a-propanocyclopenta[8]annulen-5-yl-2-((4-(((3-bromophenyl)sulfonyl)oxy)-5-methoxypyrimidin-2-yl)thio)acetate (J7): Yield: 83%; white solid; m.p. 82.9-84.6°C; ^{1}H NMR (600 MHz $CDCl_3$) δ 8.21(s, 1 H), 8.13(s, 1 H), 8.00 – 7.98 (m, 1 H), 7.82 – 7.79 (m, 1 H), 7.46 (t, J = 8.0 Hz, 1 H), 6.40 (dd, J = 17.5, 11.0 Hz, 1 H), 5.71 (d, J = 8.5 Hz, 1 H), 5.25 (dd, J = 11.0, 1.2 Hz, 1 H), 5.14 (dd, J = 17.5, 1.4 Hz, 1 H), 3.87(s, 3 H), 3.69(s, 2 H), 3.31 (dd, J = 10.6, 6.5 Hz, 1 H), 2.29 – 2.24 (m, 1 H), 2.24 – 2.12 (m, 2 H), 2.05(s, 1 H), 2.02 – 1.95 (m, 1 H), 1.72 (dd, J = 14.5, 2.8 Hz, 1 H), 1.61 (dd, J = 9.4, 2.7 Hz, 2 H), 1.53 – 1.47 (m, 1 H), 1.46 – 1.39 (m, 2 H), 1.37(s, 3 H), 1.32 (dd, J = 14.3, 3.1 Hz, 1 H), 1.25 (d, J = 16.1 Hz, 1 H), 1.12(s, 3 H), 1.08 (dd, J = 14.1, 4.3 Hz, 1 H), 0.83 (d, J = 7.0 Hz, 3 H), 0.67 (d, J = 7.0 Hz, 3 H). ^{13}C NMR (151 MHz $CDCl_3$) δ 217.14, 167.52, 160.00, 153.19, 143.27, 141.06, 139.16, 138.52, 137.72, 132.05, 130.64, 127.66, 122.94, 117.20, 74.62, 69.88, 58.20, 57.11, 53.55, 45.52, 44.61, 43.97, 41.91, 36.81, 36.07, 34.55, 30.48, 26.91, 26.47, 24.89, 16.79, 14.95, 11.55. HRMS (ESI): calculated for $C_{33}H_{41}O_8N_2BrNaS_2$ $[M+Na]^+$: 759.1380, found 759.1351.

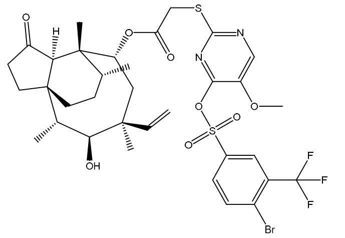

(3aR,4R,5R,7S,8S,9R,9aS,12R)-8-hydroxy-4,7,9,12-tetramethyl-3-oxo-7-vinyldecahydro-4,9a-propanocyclopenta[8]annulen-5-yl-2-((4-(((4-bromo-3-(trifluoromethyl)phenyl)sulfonyl)oxy)-5-methoxypyrimidin-2-yl)thio)acetate (J8): Yield: 79.2%; white solid; m.p. 74.8-76.2 °C; ^{1}H NMR (600 MHz $CDCl_3$) δ 8.41 (d, J = 1.9 Hz, 1 H), 8.14(s, 1 H), 8.10 (dd, J = 8.5, 2.1 Hz, 1 H)), 7.97 (d, J = 8.4 Hz, 1 H), 6.42 (dd, J = 17.4, 11.0 Hz, 1 H), 5.73 (d, J = 8.5 Hz, 1 H), 5.28 – 5.25 (m, 1H), 5.16 (dd, J = 17.5, 1.2 Hz, 1 H), 3.89(s, 3 H), 3.73(s, 2 H), 3.33(s, 1 H), 2.30 – 2.26 (m, 1 H), 2.20 (ddt, J = 28.9, 19.4, 10.1 Hz, 2 H), 2.07(s, 1 H), 2.04 – 1.99 (m, 1 H), 1.79 – 1.71 (m, 1 H), 1.65 – 1.60 (m, 2 H), 1.54 – 1.49 (m,1 H), 1.47 – 1.41 (m, 2 H), 1.39(s, 3 H), 1.36 – 1.32 (m, 1 H), 1.27 (d, J = 16.0 Hz, 1 H), 1.14(s, 3 H), 1.10 (dd, J = 14.2, 4.4 Hz, 1 H), 0.84 (d, J = 7.0 Hz, 3 H), 0.70 (d, J = 7.1 Hz, 3 H). ^{13}C NMR (151 MHz $CDCl_3$) δ 217.09, 167.49, 159.96 153.08, 143.16, 140.80, 139.18, 136.50 136.09, 133.18, 128.93, 127.88, 117.17, 74.62, 69.94, 58.19, 57.06, 53.54, 45.51, 44.63, 43.96, 41.91, 36.80, 36.08, 34.53, 30.48, 29.77, 26.91, 26.47, 24.89, 16.74, 14.88, 11.53. HRMS (ESI): calculated for $C_{34}H_{40}O_8N_2F_3NaS_2$ $[M+Na]^+$: 827.1254, found 827.1216.

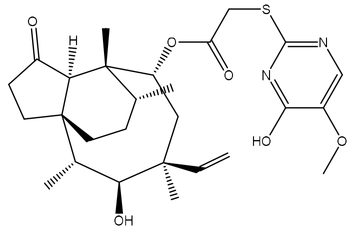

(3aR,4R,5R,7S,8S,9R,9aS,12R)-8-hydroxy-4,7,9,12-tetramethyl-3-oxo-7-vinyldecahydro-4,9a-propanocyclopenta[8]annulen-5-yl-2-((4-hydroxy-5-methoxypyrimidin-2-yl)thio)acetate

(J9): Yield: 79.3%; yellow solid; m.p. 133.6-135.2°C; ^{1}H NMR (400 MHz $CDCl_3$) δ 12.47 (s, 1 H), 7.30 (s, 1 H), 6.44 (dd, J = 17.4, 11.0 Hz, 1 H), 5.76 (d, J = 8.4 Hz, 1 H), 5.30 (d, J = 13.6 Hz, 2 H), 5.21 – 5.14 (m, 1H), 3.79 (s, 3 H), 3.41 – 3.31 (m, 1 H), 2.31 – 2.26 (m, 1H), 2.20 (dt, J = 19.6, 9.3 Hz, 2 H), 2.08 (s, 1 H), 2.04 – 2.00 (m, 1 H), 1.78 – 1.73 (m, 1 H), 1.68 – 1.61 (m, 2 H), 1.61 – 1.55 (m, 1 H), 1.49 (dd, J = 24.2, 8.3 Hz, 2 H), 1.42 (s, 3 H), 1.38 – 1.34 (m, 1 H), 1.32 – 1.25 (m, 2 H), 1.14 (s, 3 H), 1.09 (dd, J = 14.1, 4.6 Hz, 1 H), 0.85 (d, J = 6.9 Hz, 3 H), 0.72 (d, J = 6.9 Hz, 3 H). ^{13}C NMR (101 MHz $CDCl_3$) δ 217.24, 167.16, 159.62, 149.45, 144.36, 139.02, 131.01, 117.28, 74.56, 70.06, 58.14, 56.36, 45.47, 44.39, 43.87, 41.83, 36.72, 36.02, 34.49, 33.78, 30.40, 26.85, 26.46, 24.84, 16.71, 14.90, 11.57. HRMS (ESI): calculated for $C_{27}H_{38}O_6N_2NaS$ $[M+Na]^+$: 541.2343, found 541.2325.

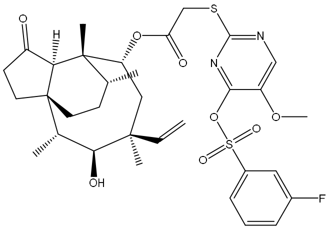

(3aR,4R,5R,7S,8S,9R,9aS,12R)-8-hydroxy-4,7,9,12-tetramethyl-3-oxo-7-vinyldecahydro-4,9a-propanocyclopenta[8]annulen-5-yl-2-((4-(((3-fluorophenyl)sulfonyl)oxy)-5-methoxypyrimidin-2-yl)thio)acetate (J10): Yield: 84%; white solid; m.p.74.8- 76.3°C; ^{1}H NMR (600 MHz $CDCl_3$) δ 8.14(s, 1 H), 7.86 (d, J = 7.8 Hz, 1 H), 7.78 (dt, J = 7.7, 1.7 Hz, 1 H), 7.58 (td, J = 8.1, 5.2 Hz, 1 H), 7.40 (td, J = 8.2, 2.1 Hz, 1 H), 6.41 (dd, J = 17.5, 11.0 Hz, 1 H), 5.71 (d, J = 8.5 Hz, 1 H), 5.30 – 5.24 (m, 2 H), 5.15 (dd, J = 17.3, 1.0 Hz, 1 H), 3.87(s, 3 H), 3.68(s, 2 H), 3.32 (dd, J = 10.5, 6.6 Hz, 1 H), 2.29 – 2.25 (m, 1 H), 2.19 (ddt, J = 28.9, 19.4, 9.8 Hz, 2 H), 2.05(s, 1 H), 2.03 – 1.95 (m, 1 H), 1.73 (dd, J = 14.5, 2.6 Hz,1 H), 1.64 – 1.58 (m, 2 H), 1.53 – 1.47 (m, 1 H), 1.46 – 1.40 (m, 2 H), 1.38(s, 3 H), 1.34 – 1.31 (m, 1 H), 1.26 (d, J = 16.1 Hz, 1 H), 1.13(s, 3 H),

0.83 (d, *J* = 7.1 Hz, 3 H), 0.68 (d, *J* = 7.1 Hz, 3 H). ^{13}CNMR (151 MHz $CDCl_3$) δ 217.11, 167.52, 163.04, 161.36, 160.04, 153.19, 143.35, 141.16, 139.15, 130.99, 124.91, 122.09, 121.95, 117.19, 116.59, 116.42, 74.63, 69.91, 58.20, 57.12, 53.53, 45.52, 44.61, 43.97, 41.91, 36.81, 36.07, 34.54, 30.48, 26.91, 26.46, 24.89, 16.76, 14.90, 11.54. HRMS (ESI): calculated for $C_{33}H_{41}O_8N_2FNaS_2$ $[M+Na]^+$: 699.2181, found 699.2154.

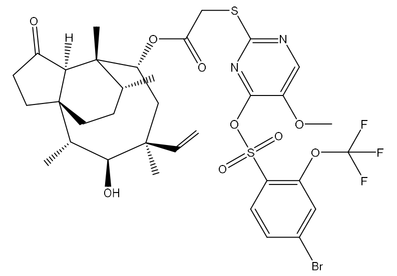

(3aR,4R,5R,7S,8S,9R,9aS,12R)-8-hydroxy-4,7,9,12-tetramethyl-3-oxo-7-vinyldecahydro-4,9a-propanocyclopenta[8]annulen-5-yl-2-((4-(((4-bromo-2-(trifluoromethoxy)phenyl)sulfonyl)oxy)-5-methoxypyrimidin-2-yl)thio)acetate (J11): Yield: 78%; white solid; m.p. 71.6-72.9 °C; ^{1}H NMR (600 MHz $CDCl_3$) δ 8.15(s, 1 H), 7.98 (d, *J* = 8.4 Hz, 1 H), 7.67 – 7.61 (m, 2 H), 6.43 (dd, *J* = 17.4, 11.0 Hz, 1 H), 5.72 (d, *J* = 8.5 Hz, 1 H), 5.28 (dd, *J* = 11.0, 1.1 Hz, 1 H), 5.16 (dd, *J* = 17.3, 1.2 Hz, 1 H), 3.88(s, 3 H), 3.65 (d, *J* = 2.1 Hz, 2 H), 3.33 (d, *J* = 6.5 Hz, 1 H), 2.28 (d, *J* = 6.9 Hz, 1 H), 2.19 (tt, *J* = 19.4, 9.6 Hz, 2 H), 2.08 – 2.00 (m, 2 H), 1.76 – 1.73 (m, 1 H), 1.63 (dd, *J* = 12.2, 2.5 Hz, 2 H), 1.53 – 1.48 (m, 1 H), 1.44 (ddd, *J* = 17.6, 12.9, 7.4 Hz, 2 H), 1.39(s, 3 H), 1.34 (dd, *J* = 14.2, 2.9 Hz, 1 H), 1.28 (d, *J* = 16.0 Hz, 1 H), 1.14(s, 3 H), 1.10 (dd, *J* = 14.1, 4.3 Hz, 1 H), 0.85 (d, *J* = 7.1 Hz, 3 H), 0.68 (d, *J* = 7.1 Hz, 3 H). ^{13}C NMR (151 MHz $CDCl_3$) δ 217.09, 167.45, 159.94, 153.17, 146.86, 143.57, 140.92, 139.15, 133.01, 130.49, 129.98, 128.85, 124.12, 117.20, 74.63, 69.96, 58.19, 57.17, 45.52, 44.64, 43.98, 41.92, 36.80, 36.07, 34.53, 30.48, 26.95, 26.44, 24.89, 16.78, 14.88, 11.54. HRMS (ESI): calculated for $C_{34}H_{40}O_9N_2BrF_3NaS_2[M+Na]^+$: 843.1203, found 843.1171.

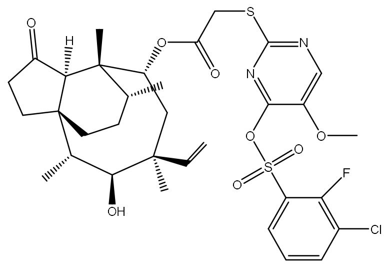

(3aR,4R,5R,7S,8S,9R,9aS,12R)-8-hydroxy-4,7,9,12-tetramethyl-3-oxo-7-vinyldecahydro-4,9a-propanocyclopenta[8]annulen-5-yl2-((4-(((3-chloro-2-fluorophenyl)sulfonyl)oxy)-5-methoxypyrimidin-2-yl)thio)acetate (J12): Yield: 79%; yellow solid; m.p. 98.3-99.8 °C; ^{1}H NMR (600 MHz $CDCl_3$) *δ* 8.17(s, 1 H), 7.89 (ddd, *J* = 7.7, 6.1, 1.5 Hz, 1 H), 7.77 – 7.73 (m, 1 H), 7.31 (t, *J* = 8.0 Hz, 1 H), 6.41 (dd, *J* = 17.5, 11.0 Hz, 1 H), 5.71 (d, *J* = 8.5 Hz, 1 H), 5.27 (dd, *J* = 11.0, 1.1 Hz, 1 H), 5.16 (dd, *J* = 17.7, 1.1 Hz, 1 H), 3.89(s, 3 H), 3.63(s, 2 H), 3.33(s, 1 H), 2.27 (d, *J* = 6.8 Hz, 1 H), 2.19 (dq, *J* = 19.6, 10.0 Hz, 2 H), 2.07(s, 1 H), 2.00 (dd, *J* = 16.0, 8.5 Hz, 1 H), 1.75 – 1.71 (m, 1 H), 1.65 – 1.60 (m, 2 H), 1.50 (d, *J* = 10.6 Hz, 1 H), 1.45 – 1.40 (m, 2 H), 1.39(s, 3 H), 1.35 – 1.32 (m, 1 H), 1.26 (d, *J* = 16.0 Hz, 1 H), 1.14(s, 3 H), 1.09 (dd, *J* = 14.1, 4.3 Hz, 1 H), 0.84 (d, *J* = 7.0 Hz, 3 H), 0.68(s, 3 H). ^{13}C NMR (151 MHz $CDCl_3$-*d*) *δ* 217.14, 167.44, 160.03, 153.07, 143.70, 141.04, 139.14, 137.23, 129.61, 127.09, 124.76, 123.49, 117.21, 74.63, 69.94, 58.19, 57.21, 45.51, 44.63, 43.97, 41.91, 36.79, 36.06, 34.54, 30.47, 26.90, 26.45, 24.89, 16.75, 14.91, 11.54. HRMS (ESI): calculated for $C_{33}H_{40}O_8N_2ClFNaS_2[M+Na]^+$: 733.1791, found 733.1763.

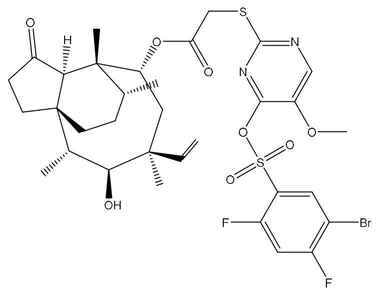

(3aR,4R,5R,7S,8S,9R,9aS,12R)-8-hydroxy-4,7,9,12-tetramethyl-3-oxo-7-vinyldecahydro-4,9a-propanocyclopenta[8]annulen-5-yl-2-((4-(((5-bromo-2,4-difluorophenyl)sulfonyl)oxy)-5-

methoxypyrimidin-2-yl)thio)acetate (J13): Yield: 74%; white solid; m.p. 79.8-81.2 °C; ^{1}H NMR (600 MHz $CDCl_3$) δ 8.24 (t, J = 7.0 Hz, 1 H), 8.15(s, 1 H), 7.10 (t, J = 8.5 Hz, 1 H), 6.40 (dd, J = 17.3, 11.0 Hz, 1 H), 5.71 (d, J = 8.5 Hz, 1 H), 5.30 – 5.23 (m, 2 H), 5.15 (dd, J = 17.3, 1.0 Hz, 1 H), 3.88(s, 3 H), 3.67(s, 2 H), 3.31 (dd, J = 10.1, 6.7 Hz, 1 H), 2.28 – 2.24 (m, 1 H), 2.18 (ddt, J = 28.9, 19.3, 10.1 Hz, 2 H), 2.05(s, 1 H), 2.02 – 1.96 (m, 1 H), 1.75 – 1.70 (m, 1 H), 1.64 – 1.58 (m, 2 H), 1.50 (dd, J = 14.8, 11.7 Hz, 1 H), 1.47 – 1.40 (m, 2 H), 1.37(s, 3 H), 1.34 – 1.30 (m, 1 H), 1.26 (d, J = 16.1 Hz, 1 H), 1.12(s, 3 H), 0.83 (d, J = 7.0 Hz, 3 H), 0.68 (d, J = 7.1 Hz, 3 H). ^{13}C NMR (151 MHz $CDCl_3$) δ 217.09, 167.41, 160.02, 153.05, 143.60, 140.85, 139.18, 136.11, 123.05, 117.18, 107.30, 107.13, 106.95, 104.86, 104.71 74.62, 69.95, 58.18, 57.18, 53.54, 45.51, 44.63, 43.97, 41.91, 36.79, 36.07, 34.50, 30.47, 26.91, 26.47, 24.89, 16.78, 14.89, 14.28, 11.54. HRMS (ESI): calculated for $C_{33}H_{39}O_8N_2BrF_2NaS_2$[M+Na]$^+$: 795.1191, found 795.1161.

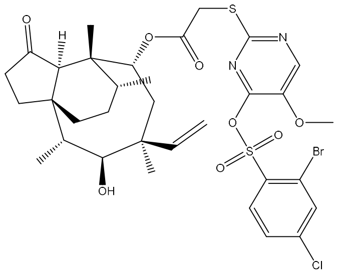

(3aR,4R,5R,7S,8S,9R,9aS,12R)-8-hydroxy-4,7,9,12-tetramethyl-3-oxo-7-vinyldecahydro-4,9a-propanocyclopenta[8]annulen-5-yl-2-((4-(((2-bromo-4-chlorophenyl)sulfonyl)oxy)-5-methoxypyrimidin-2-yl)thio)acetate (J14): Yield: 81%; yellow solid; m.p. 81.2-82.9 °C; ^{1}H NMR (600 MHz $CDCl_3$) δ 8.33 (dd, J = 6.1, 2.2 Hz, 1 H), 8.13(s, 1 H), 8.04 (ddd, J = 8.5, 4.2, 2.3 Hz, 1 H), 7.31 (t, J = 8.2 Hz, 1 H), 6.41 (dd, J = 17.3, 11.0 Hz, 1 H), 5.72 (d, J = 8.5 Hz, 1 H), 5.34 – 5.21 (m, 2 H), 5.15 (dd, J = 17.3, 1.1 Hz, 1 H), 3.88(s, 3 H), 3.72(s, 2 H), 3.32 (d, J = 6.5 Hz, 1 H), 2.28 – 2.25 (m, 1 H), 2.18 (tt, J = 19.4, 9.5 Hz, 2 H), 2.05(s, 1 H), 2.02 – 1.97 (m, 1 H), 1.74 – 1.71 (m, 1 H), 1.64 – 1.59 (m, 2 H), 1.52 – 1.47 (m, 1 H), 1.47 – 1.39 (m, 2 H), 1.38(s, 3 H), 1.34 – 1.31 (m,

1 H), 1.26 (d, J = 16.3 Hz, 1 H), 1.12(s, 3 H), 0.83 (d, J = 7.0 Hz, 3 H), 0.68 (d, J = 7.1 Hz, 3 H). ^{13}C NMR (151 MHz $CDCl_3$) δ 217.11, 167.52, 163.75, 162.04, 159.92, 153.20, 143.15, 140.90, 139.17, 135.34, 130.71, 117.38, 117.20, 110.20, 74.62, 69.93, 58.20, 57.10, 53.54, 45.52, 44.62, 43.97, 41.91, 36.80, 36.07, 34.54, 30.48, 26.91, 26.47, 24.89, 16.78, 14.91, 11.54. HRMS (ESI): calculated for $C_{33}H_{40}O_8N_2BrClNaS_2$ [M+Na]$^+$: 793.10, found 793.10.

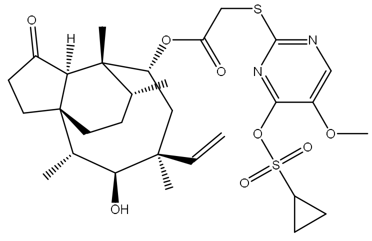

(3aR,4R,5R,7S,8S,9R,9aS,12R)-8-hydroxy-4,7,9,12-tetramethyl-3-oxo-7-vinyldecahydro-4,9a-propanocyclopenta[8]annulen-5-yl-2-((4-((cyclopropylsulfonyl)oxy)-5-methoxypyrimidin-2-yl)thio)acetate (J15): Yield: 82%; white solid; m.p. 75.2-76.9 °C, ^{1}H NMR (600 MHz $CDCl_3$) δ 8.14(s, 1 H), 6.41 (dd, J = 17.4, 11.0 Hz, 1 H), 5.72 (d, J = 8.5 Hz, 1 H), 5.28 – 5.24 (m, 1 H), 5.14 (d, J = 17.9 Hz, 1 H), 3.88(s, 3 H), 3.79(s, 2 H), 3.32 (d, J = 5.8 Hz, 1 H), 3.19 (tt, J = 8.2, 4.8 Hz, 1 H), 2.28 – 2.24 (m, 1 H), 2.17 (ddd, J = 28.9, 18.5, 9.8 Hz, 2 H), 2.06(s, 1 H), 2.00 (dt, J = 15.5, 7.7 Hz, 1 H), 1.76 – 1.69 (m, 2 H), 1.62(s, 1 H), 1.59 (d, J = 10.7 Hz, 1 H), 1.49 (d, J = 13.7 Hz, 1 H), 1.44 (dd, J = 4.7, 2.0 Hz, 3 H), 1.40(s, 3 H), 1.33(s, 1 H), 1.29 (d, J = 16.1 Hz, 1 H), 1.25 – 1.21 (m, 2 H), 1.12(s, 3 H), 1.10 – 1.05 (m, 1 H), 0.83 (d, J = 7.0 Hz, 3 H), 0.70 (d, J = 7.1 Hz, 3 H). ^{13}C NMR (151 MHz $CDCl_3$) δ 217.15, 167.76, 159.92, 153.77, 143.13, 141.12, 139.21, 117.17, 74.61, 69.98, 58.19, 57.15, 45.52, 44.64, 43.99, 41.93, 36.80, 36.07, 34.70, 34.61, 31.41, 30.47, 26.92, 26.50, 24.89, 16.82, 14.94, 11.55, 7.27. HRMS (ESI): calculated for $C_{30}H_{42}O_8N_2NaS_2$ [M+Na]$^+$: 645.2275, found 645.2250.

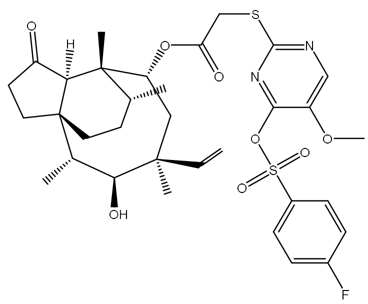

(3aR,4R,5R,7S,8S,9R,9aS,12R)-8-hydroxy-4,7,9,12-tetramethyl-3-oxo-7-vinyldecahydro-4,9a-propanocyclopenta[8]annulen-5-yl-2-((4-(((4-fluorophenyl)sulfonyl)oxy)-5-methoxypyrimidin-2-yl)thio)acetate (J16): Yield: 79%; white solid; m.p. 82.6-84.1 °C; ^{1}H NMR (600 MHz CDCl$_3$) δ 8.11(s, 1 H), 8.10 – 8.07 (m, 2 H), 7.25 (t, J = 8.5 Hz, 2 H), 6.40 (dd, J = 17.5, 11.0 Hz, 1 H), 5.71 (d, J = 8.5 Hz, 1 H), 5.28 – 5.22 (m, 2 H), 5.16 – 5.11 (m, 1 H), 3.86(s, 3 H), 3.69(s, 2 H), 3.31 (dd, J = 10.5, 6.6 Hz, 1 H), 2.28 – 2.24 (m, 1 H), 2.17 (tt, J = 19.4, 9.6 Hz, 2 H), 2.05(s, 1 H), 2.02 – 1.95 (m, 1 H), 1.72 (d, J = 13.7 Hz, 1 H), 1.61 (ddd, J = 12.5, 6.4, 4.0 Hz, 2 H), 1.50 (dd, J = 13.9, 10.8 Hz, 1 H), 1.47 – 1.40 (m, 2 H), 1.38(s, 3 H), 1.32 (dd, J = 14.3, 3.0 Hz, 1 H), 1.26 (d, J = 16.0 Hz, 1 H), 1.12(s, 3 H), 0.83 (d, J = 7.1 Hz, 3 H), 0.68 (d, J = 7.1 Hz, 3 H). ^{13}C NMR (151 MHz CDCl$_3$) δ 217.10, 167.56, 167.20, 165.49, 159.87, 153.34, 143.15, 141.06, 139.17, 132.71, 132.23, 117.18, 116.66, 116.51, 74.62, 69.92, 58.19, 57.10, 45.51, 44.61, 43.97, 41.91, 36.80, 36.07, 34.54, 30.47, 26.91, 26.47, 24.89, 16.77, 14.90, 11.54. HRMS (ESI): calculated for $C_{33}H_{41}O_8N_2FNaS_2$ [M+Na]$^+$: 699.2181, found 699.2151.

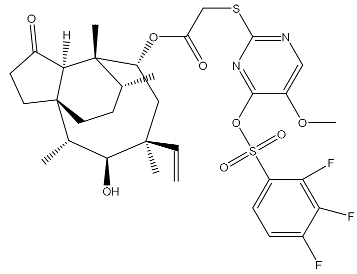

(3aR,4R,5R,7S,8S,9R,9aS,12R)-8-hydroxy-4,7,9,12-tetramethyl-3-oxo-7-vinyldecahydro-4,9a-propanocyclopenta[8]annulen-5-yl-2-((5-methoxy-4-(((2,3,4-

trifluorophenyl)sulfonyl)oxy)pyrimidin-2-yl)thio)acetate (J17): Yield: 79%; Yellow solid; m.p. 59.2-60.4 °C; ^{1}H NMR (600 MHz $CDCl_3$) *δ* 8.16(s, 1 H), 7.84 – 7.71 (m, 1 H), 7.21 – 7.15 (m, 1 H), 6.41 (dd, *J* = 17.3, 11.0 Hz, 1 H), 5.71 (d, *J* = 8.5 Hz,1 H), 5.35 – 5.09 (m, 3 H), 3.89(s, 3 H), 3.66(s, 2 H), 3.32 (d, *J* = 6.5 Hz, 1 H), 2.28 – 2.25 (m, 1 H), 2.19 (dq, *J* = 19.5, 9.9 Hz, 2 H), 2.06(s, 1 H), 2.02 – 1.98 (m, 1 H), 1.75 – 1.71 (m, 1 H), 1.64 – 1.60 (m, 2 H), 1.50 – 1.46 (m, 1 H), 1.44 – 1.40 (m, 2 H), 1.38(s, 3 H), 1.34 (d, *J* = 3.1 Hz, 1 H), 1.27 – 1.24 (m, 1 H), 1.13(s, 3 H), 0.84 (d, *J* = 7.0 Hz, 3 H), 0.68 – 0.66 (m, 3 H). ^{13}C NMR (151 MHz $CDCl_3$) *δ* 217.11, 167.42, 159.99, 153.05, 143.60, 140.97, 139.13, 125.91, 123.14, 117.46, 117.20, 112.76, 74.63, 70.00, 61.40, 58.19, 57.21, 53.53, 45.52, 44.63, 43.97, 41.91, 36.79, 36.07, 34.53, 30.47, 29.77, 26.90, 26.45, 24.89, 16.74, 14.85, 11.54. HRMS (ESI): calculated for $C_{33}H_{39}O_8N_2F_3NaS_2$ $[M+Na]^+$: 735.1992, found 735.1962.

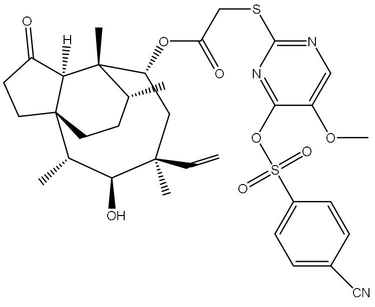

(3aR,4R,5R,7S,8S,9R,9aS,12R)-8-hydroxy-4,7,9,12-tetramethyl-3-oxo-7-vinyldecahydro-4,9a-propanocyclopenta[8]annulen-5-yl-2-((4-(((4-cyanophenyl)sulfonyl)oxy)-5-methoxypyrimidin-2-yl)thio)acetate (J18): Yield: 83.2%; yellow solid; m.p. 89.4-91.2 °C; ^{1}H NMR (600 MHz $CDCl_3$) *δ* 8.20 – 8.18 (m,2 H), 8.13(s, 1 H), 7.89 – 7.87 (m, 2 H), 6.41 (dd, *J* = 17.3, 11.0 Hz, 1 H), 5.27(s, 1 H), 5.25 (dd, *J* = 11.1, 1.1 Hz, 1 H), 5.15 (dd, *J* = 17.5, 1.4 Hz, 1 H), 3.87(s, 3 H), 3.69(s, 2 H), 3.32 (dd, *J* = 10.1, 6.6 Hz, 1 H), 2.28 – 2.24 (m, 1 H), 2.17 (tt, *J* = 19.4, 9.5 Hz, 2 H), 2.06(s, 1 H), 2.00 (dd, *J* = 16.0, 8.6 Hz, 1 H), 1.76 – 1.70 (m, 2 H), 1.64 – 1.58 (m, 2 H), 1.52 – 1.47 (m, 1 H), 1.46 – 1.39 (m, 2 H), 1.38(s, 3 H), 1.33 (dd, *J* = 14.5, 3.2 Hz, 1 H), 1.26

(d, J = 16.1 Hz, 1 H), 1.12(s, 3 H), 0.83 (d, J = 7.0 Hz, 3 H), 0.68 (d, J = 7.1 Hz, 3 H). ^{13}C NMR (151 MHz $CDCl_3$) δ 217.10, 167.47, 159.88, 153.03, 143.32, 140.95 139.18, 132.93, 129.80, 118.29, 117.20, 117.03, 74.60, 70.01, 58.16, 57.11, 53.56, 45.51, 44.64, 43.98, 41.91, 36.78, 36.07, 34.52, 30.46, 26.91 26.49, 24.88, 16.80, 14.90, 11.54. HRMS (ESI): calculated for $C_{34}H_{41}O_8N_3NaS_2$ [M+Na]$^+$: 706.2227, found 706.2189.

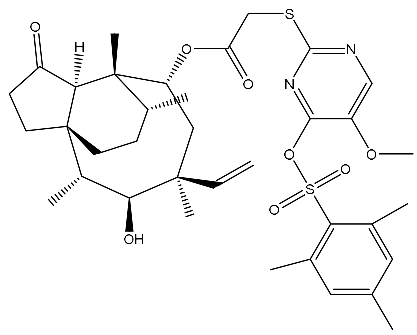

(3aR,4R,5R,7S,8S,9R,9aS,12R)-8-hydroxy-4,7,9,12-tetramethyl-3-oxo-7-vinyldecahydro-4,9a-propanocyclopenta[8]annulen-5-yl-2-((4-((mesitylsulfonyl)oxy)-5-methoxypyrimidin-2-yl)thio)acetate (J19): Yield: 79.8%; white solid; m.p. 76.3-78.2 °C; ^{1}H NMR (600 MHz $CDCl_3$) δ 8.04(s, 1 H), 6.97(s, 2 H), 6.41 (dd, J = 17.4, 11.0 Hz, 1 H), 5.69 (d, J = 8.5 Hz, 1 H), 5.27 (d, J = 11.0 Hz, 1 H), 5.17 – 5.11 (m, 1 H), 3.85(s, 3 H), 3.59 (d, J = 2.4 Hz, 2 H), 3.34 – 3.29 (m, 1 H), 2.66(s, 6 H), 2.30(s, 3 H), 2.27 – 2.24 (m, 1 H), 2.18 (td, J = 19.5, 18.3, 9.9 Hz, 2 H), 2.05(s, 1 H), 2.02 – 1.96 (m, 1 H), 1.75 – 1.70 (m, 2 H), 1.64 – 1.59 (m, 1 H), 1.47(s, 1 H), 1.42 (dd, J = 12.6, 8.9 Hz, 2 H), 1.35(s, 3 H), 1.33 – 1.29 (m, 1 H), 1.24 (d, J = 7.5 Hz, 1 H), 1.12(s, 3 H), 1.08 (dd, J = 14.0, 4.2 Hz, 1 H), 0.83 (d, J = 7.0 Hz, 3 H), 0.65 (d, J = 7.1 Hz, 3 H). ^{13}C NMR (151 MHz $CDCl_3$) δ 217.11, 167.63, 159.70, 154.07, 144.27, 142.59, 140.46, 139.18, 132.39, 131.84, 117.18, 74.61, 69.79, 58.20, 57.11, 45.51, 44.64, 43.97, 41.90, 36.81, 36.07, 34.50, 30.48, 26.91, 26.51, 24.89, 22.82, 21.25, 16.75, 14.87, 11.53. HRMS (ESI): calculated for $C_{36}H_{48}O_8N_2NaS_2$ [M+Na]$^+$: 723.2744, found 723.2717.

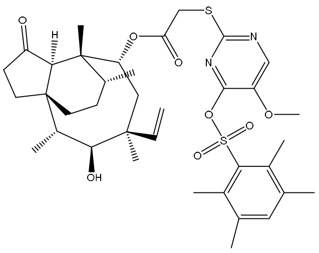

(3aR,4R,5R,7S,8S,9R,9aS,12R)-8-hydroxy-4,7,9,12-tetramethyl-3-oxo-7-vinyldecahydro-4,9a-propanocyclopenta[8]annulen-5-yl2-((5-methoxy-4-(((2,3,5,6-tetramethylphenyl)sulfonyl)oxy)pyrimidin-2-yl)thio)acetate (J20): Yield: 83.5%; Yellow solid; m.p. 98.1-99.4 °C; ^{1}H NMR (600 MHz CDCl$_3$) δ 8.05(s, 1 H), 7.20(s, 1 H), 6.41 (dd, J = 17.4, 11.0 Hz, 1 H), 5.69 (d, J = 8.5 Hz, 1 H), 5.38 – 5.05 (m, 2 H), 3.86(s, 3 H), 3.56(s, 2 H), 3.39 – 3.26 (m, 1 H), 2.57(s, 6 H), 2.27(s, 6 H), 2.24 (d, J = 6.6 Hz, 1 H), 2.18 (tt, J = 19.3, 9.7 Hz, 2 H), 2.05(s, 1 H), 1.99 (dd, J = 15.9, 8.7 Hz, 1 H), 1.74 – 1.70 (m, 1 H), 1.64 – 1.57 (m, 2 H), 1.52 – 1.47 (m, 1 H), 1.46 – 1.39 (m, 2 H), 1.35(s, 3 H), 1.33 – 1.28 (m, 1 H), 1.24 (d, J = 16.0 Hz, 1 H), 1.12(s, 3 H), 1.08 (dd, J = 14.1, 4.2 Hz, 1 H), 0.83 (d, J = 7.0 Hz, 3 H), 0.63 (d, J = 7.0 Hz, 3 H). ^{13}C NMR (126 MHz DMSO) δ 217.71, 167.34, 158.73, 153.30, 144.31, 141.26, 140.47, 137.73, 136.66, 136.28, 135.87, 115.76, 73.05, 70.46, 57.58, 45.46, 44.45, 44.01, 41.98, 40.49, 40.33, 40.08, 39.87, 39.74, 39.49, 36.83, 34.51, 34.14, 30.59, 29.02, 27.07, 24.96, 20.98, 17.95, 16.46, 14.88, 12.05. HRMS (ESI): calculated for $C_{37}H_{50}O_8N_2NaS_2$ [M+Na]$^+$: 737.2901, found 737.2869.

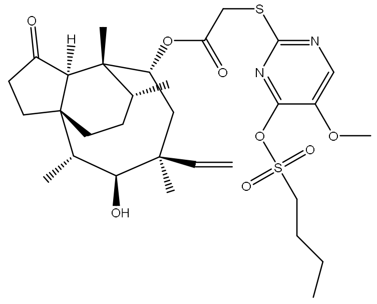

(3aR,4R,5R,7S,8S,9R,9aS,12R)-8-hydroxy-4,7,9,12-tetramethyl-3-oxo-7-vinyldecahydro-4,9a-propanocyclopenta[8]annulen-5-yl-2-((4-((butylsulfonyl)oxy)-5-methoxypyrimidin-2-

yl)thio)acetate (J21): Yield: 79.3%; Yellow solid; m.p. 71.3-72.1 °C; ^{1}H NMR (500 MHz $CDCl_3$) δ 8.17(s, 1 H), 6.45 (dd, J = 17.4, 11.0 Hz, 1 H), 5.75 (d, J = 8.5 Hz, 1 H), 5.30 – 5.27 (m, 2 H), 5.17 (dd, J = 17.3, 1.5 Hz, 1 H), 3.91(s, 3 H), 3.80 (d, J = 1.3 Hz, 2 h), 3.67(s, 1 H), 3.34 (dd, J = 9.6, 6.7 Hz, 1 H), 2.31 – 2.27 (m, 1 H), 2.25 – 2.15 (m, 2 H), 2.08(s, 1 H), 2.06 – 2.01 (m, 1 H), 2.00 – 1.94 (m, 2 H), 1.75 (dd, J = 14.4, 2.8 Hz, 1 H), 1.66 – 1.62 (m, 2 H), 1.56 (d, J = 7.4 Hz, 1 H), 1.54 – 1.50 (m, 2 H), 1.45 (dd, J = 9.7, 3.2 Hz, 2 H), 1.43(s, 3 H), 1.36 (d, J = 3.1 Hz, 1 H), 1.30 (d, J = 16.1 Hz, 1 H), 1.14(s, 3 H), 1.10 (dd, J = 14.1, 4.3 Hz, 1 H), 0.99 (t, J = 7.4 Hz, 3 H), 0.85 (d, J = 7.0 Hz, 3 H), 0.72 (d, J = 7.0 Hz, 3 H). ^{13}C NMR (126 MHz $CDCl_3$) δ 217.10, 167.66, 160.09, 153.67, 143.44, 141.58, 139.19, 117.21, 74.63, 69.97, 58.20, 57.21, 54.36, 45.53, 44.64, 44.00, 41.94, 36.81, 36.08, 34.72, 34.54, 30.48, 26.93, 26.47, 25.46, 24.89, 21.43, 16.81, 14.94, 13.57, 11.55. HRMS (ESI): calculated for $C_{31}H_{46}O_8N_2NaS_2$ $[M+Na]^+$: 661.2588, found 661.2557.

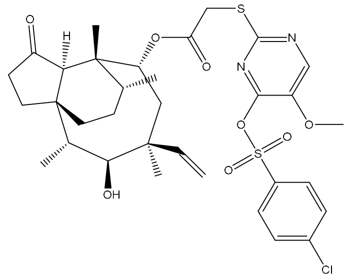

(3aR,4R,5R,7S,8S,9R,9aS,12R)-8-hydroxy-4,7,9,12-tetramethyl-3-oxo-7-vinyldecahydro-4,9a-propanocyclopenta[8]annulen-5-yl2-((4-(((4-chlorophenyl)sulfonyl)oxy)-5-methoxypyrimidin-2-yl)thio)acetate (J22): Yield: 78.4%; white solid; m.p. 79.3-81.2 °C; ^{1}H NMR (600 MHz $CDCl_3$) δ 8.13 (s,1 H), 8.05 – 7.99 (m,2 H), 7.69 – 7.47 (m,2 H), 6.42 (dd, J = 17.3, 11.0 Hz,1 H), 5.73 (d, J = 8.5 Hz,1 H), 5.32 – 5.23 (m,2 H), 5.16 (dd, J = 17.5, 1.4 Hz,1 H), 3.88 (s,3 H), 3.71 (s,2 H), 3.33 (dd, J = 10.5, 6.5 Hz,1 H), 2.30 – 2.26 (m,1 H), 2.24 – 2.14 (m,2 H), 2.07 (s,1 H), 2.04 – 1.97 (m,1 H), 1.74 (dd, J = 14.5, 2.7 Hz,1 H), 1.65 – 1.61 (m,2 H), 1.51 (dd, J = 13.2, 2.8 Hz,1 H), 1.47 – 1.42 (m,2 H), 1.39 (s,3 H), 1.36 – 1.31 (m,1 H), 1.27 (d, J = 16.0 Hz,1 H), 1.14 (s,3

H), 0.84 (d, *J* = 7.0 Hz,3 H), 0.69 (d, *J* = 7.2 Hz,3 H). ^{13}C NMR (151 MHz $CDCl_3$) δ 217.09 167.56, 159.90, 153.30, 143.18, 141.53, 141.04, 139.16, 135.20, 130.65, 129.54, 117.20, 74.63, 69.92, 58.19, 57.10, 53.54, 45.52, 44.62, 43.98, 41.91, 36.80, 36.07, 34.54, 30.48, 26.91, 26.47, 24.89, 16.79, 14.91, 11.54. HRMS (ESI): calculated for $C_{33}H_{41}O_8N_2ClNaS_2$ $[M+Na]^+$: 715.1885, found 715.1854.

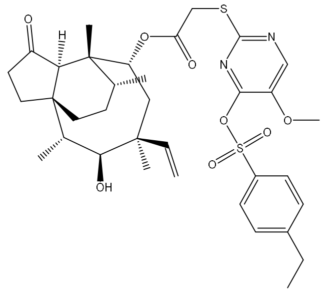

(3aR,4R,5R,7S,8S,9R,9aS,12R)-8-hydroxy-4,7,9,12-tetramethyl-3-oxo-7-vinyldecahydro-4,9a-propanocyclopenta[8]annulen-5-yl2-((4-(((4-ethylphenyl)sulfonyl)oxy)-5-methoxypyrimidin-2-yl)thio)acetate (J23): Yield: 82.1%; Yellow solid; m.p. 83.2-84.8 °C; ^{1}H NMR (600 MHz $CDCl_3$) δ 8.12(s, 1 H), 7.96 (d, *J* = 8.4 Hz, 2 H), 7.39 (d, *J* = 8.3 Hz, 2 H), 6.42 (dd, *J* = 17.3, 11.0 Hz, 1 H), 5.72 (d, *J* = 8.5 Hz, 1 H), 5.26 (dd, *J* = 11.1, 1.0 Hz, 1 H), 5.16 (dd, *J* = 17.3, 1.1 Hz, 1 H), 3.87(s, 3 H), 3.67(s, 2 H), 3.33 (dd, *J* = 9.6, 6.8 Hz, 1 H), 2.75 (q, *J* = 7.6 Hz, 2 H), 2.31 – 2.26 (m. 1 H), 2.19 (tt, *J* = 19.4, 9.5 Hz, 2 H), 2.07(s, 1 H), 1.99 (dd, *J* = 16.0, 8.5 Hz, 1 H), 1.75 – 1.72 (m, 1 H), 1.63 (dd, *J* = 12.5, 2.2 Hz, 2 H), 1.51 (dd, *J* = 15.6, 12.5 Hz, 2 H), 1.47 – 1.40 (m, 2 H), 1.38(s, 3 H), 1.33 (dd, *J* = 14.0, 3.2 Hz, 1 H), 1.29(s, 1 H), 1.27 (d, *J* = 7.6 Hz, 3 H), 1.14(s, 3 H), 0.84 (d, *J* = 7.1 Hz, 3 H)), 0.69 (d, *J* = 7.1 Hz, 3 H). ^{13}C NMR (151 MHz $CDCl_3$) δ 217.14, 167.59, 159.93, 153.51, 152.03, 143.16, 141.22, 139.15, 133.90, 129.28, 128.65, 117.19, 74.64, 69.86, 58.20, 57.12, 45.52, 44.59, 43.97, 41.91, 36.81, 36.07, 34.55, 30.48, 29.08, 26.91, 26.46, 24.89, 16.79, 15.09, 14.91, 11.54. HRMS (ESI): calculated for $C_{35}H_{46}O_8N_2NaS_2$ $[M+Na]^+$: 709.2588, found 709.2559.

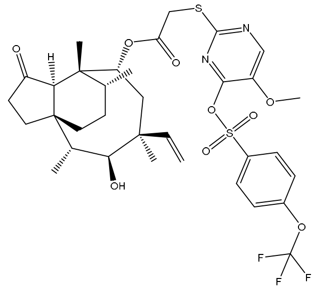

(3aR,4R,5R,7S,8S,9R,9aS,12R)-8-hydroxy-4,7,9,12-tetramethyl-3-oxo-7-vinyldecahydro-4,9a-propanocyclopenta[8]annulen-5-yl-2-((5-methoxy-4-(((4-(trifluoromethoxy)phenyl)sulfonyl)oxy)pyrimidin-2-yl)thio)acetate (J24): Yield: 78.5%; white solid; m.p. 81.3-83.0 °C; ^{1}H NMR (600 MHz $CDCl_3$) δ 8.13 (dd, J = 6.0, 2.9 Hz,3 H), 7.40 (d, J = 8.6 Hz,2 H), 6.41 (dd, J = 17.4, 11.0 Hz,1 H), 5.72 (d, J = 8.5 Hz,1 H), 5.20 (dd, J = 63.9, 14.3 Hz,2 H), 3.87 (s,3 H), 3.69 (s,2 H), 3.32 (t, J = 7.6 Hz,1 H), 2.29 – 2.25 (m,1 H), 2.23 – 2.19 (m,1 H), 2.16 (dd, J = 19.5, 9.4 Hz,1 H), 2.06 (s,1 H), 2.00 (dd, J = 16.0, 8.5 Hz,1 H), 1.73 (dd, J = 14.5, 2.5 Hz,1 H), 1.62 (dd, J = 9.6, 2.3 Hz,2 H), 1.50 (dd, J = 14.0, 2.7 Hz,1 H), 1.47 – 1.41 (m,2 H), 1.39 (s,3 H), 1.35 – 1.30 (m,1 H), 1.27 (d, J = 16.0 Hz,1 H), 1.13 (s,3 H), 1.09 (dd, J = 14.0, 4.3 Hz,1 H), 0.84 (d, J = 7.1 Hz,3 H), 0.69 (d, J = 7.1 Hz,3 H). ^{13}C NMR (126 MHz DMSO-*d6*) δ 217.70, 167.37, 159.39, 153.25, 152.41, 145.69, 141.59, 141.22, 134.95, 131.95, 122.07, 121.34, 119.28, 115.64, 73.06, 70.48, 57.63, 45.45, 44.46, 43.92, 41.95, 36.83, 34.50, 34.31, 30.58, 28.94, 27.06, 24.97, 16.51, 14.87, 12.04. HRMS (ESI): calculated for $C_{34}H_{41}O_9N_2F_3NaS_2$ $[M+Na]^+$: 765.2095, found 765.2068.

IV. ^{1}H, ^{13}C NMR, and HRMS of compound P-J9, J1-J24 data

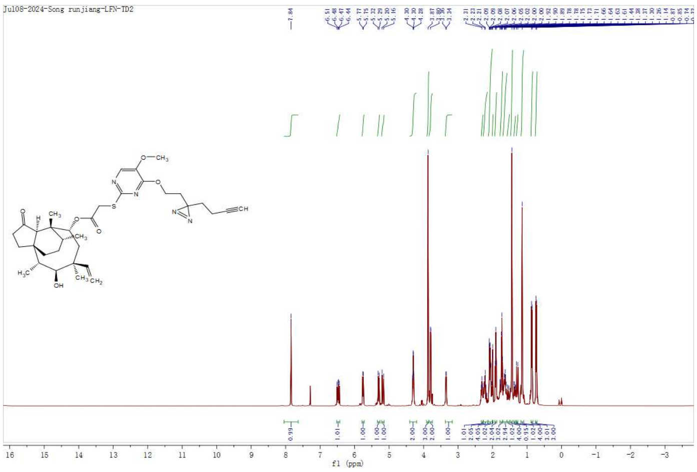

Figure S3. ^{1}H NMR (400 MHz $CDCl_3$) spectrum of compound **P-J9**.

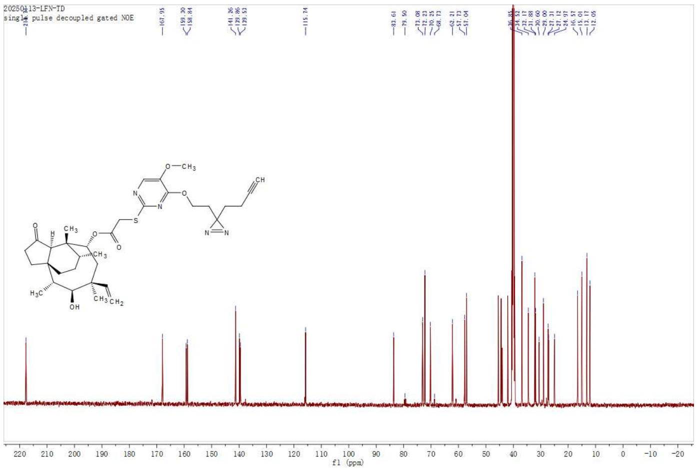

Figure S4. ^{13}C NMR (126 MHz DMSO) spectrum of compound **P-J9**.

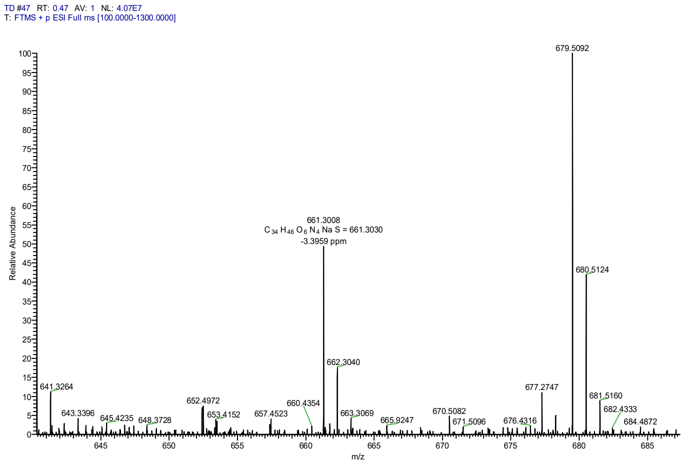

Figure S5. HRMS of compound **P-J9**.

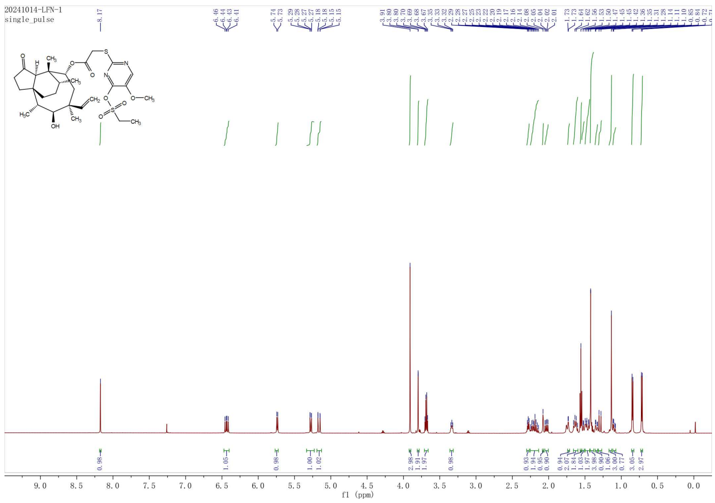

Figure S6. ^{1}H NMR (600 MHz $CDCl_3$) spectrum of compound **J1**.

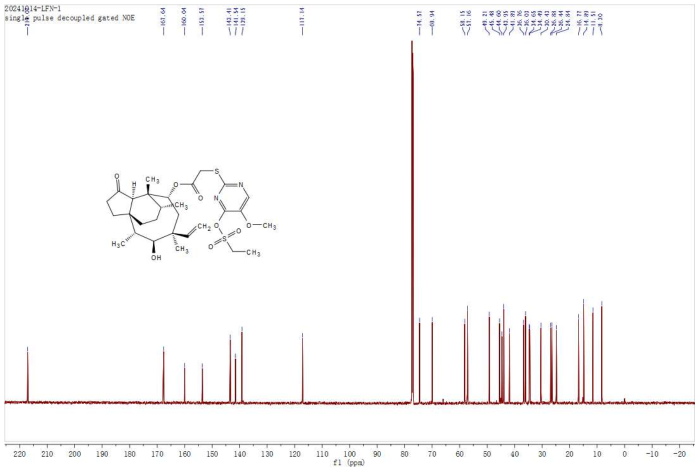

Figure S7. ^{13}C NMR (151 MHz $CDCl_3$) spectrum of compound **J1**.

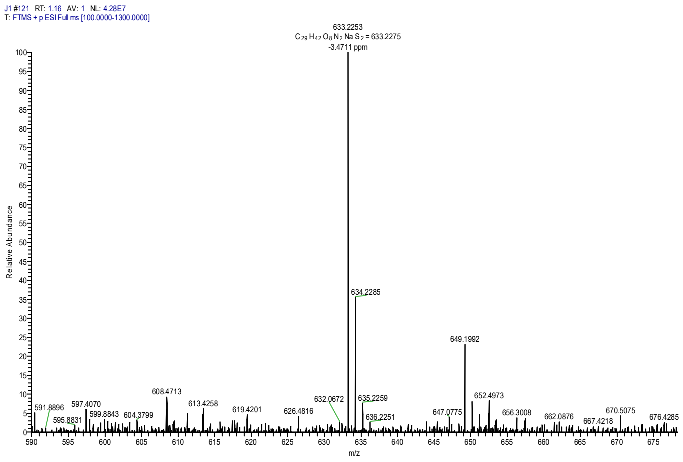

Figure S8. HRMS of compound **J1**.

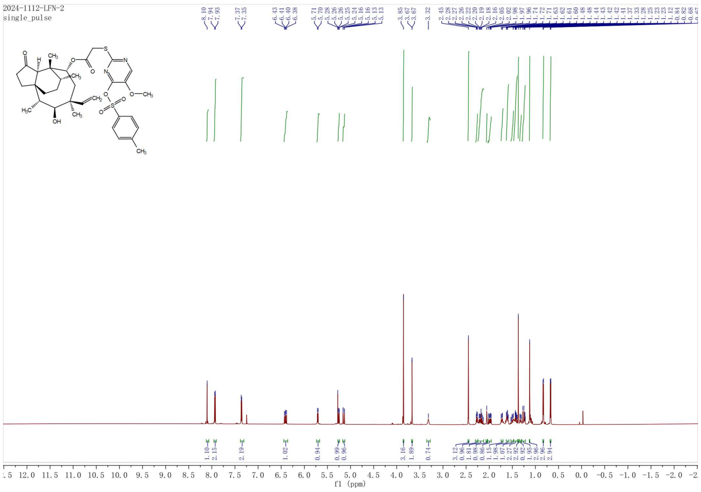

Figure S9. ^{1}H NMR (600 MHz $CDCl_3$) spectrum of compound **J2**.

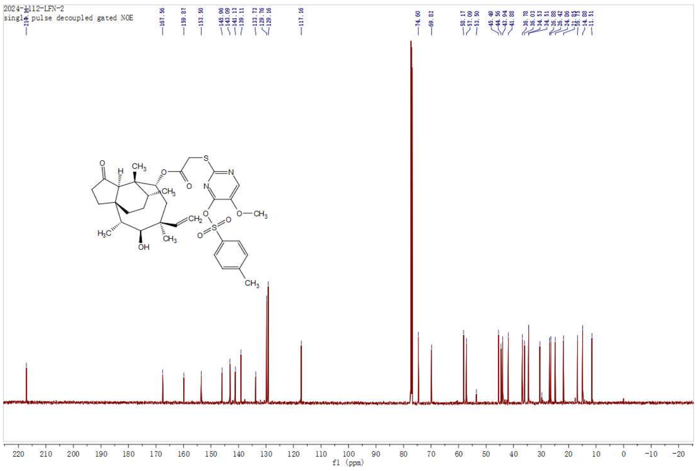

Figure S10. ^{13}C NMR (151 MHz $CDCl_3$) spectrum of compound **J2**.

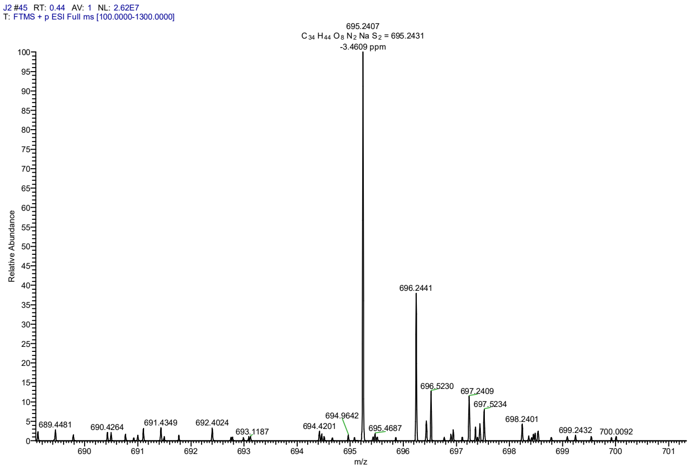

Figure S11. HRMS of compound **J2**.

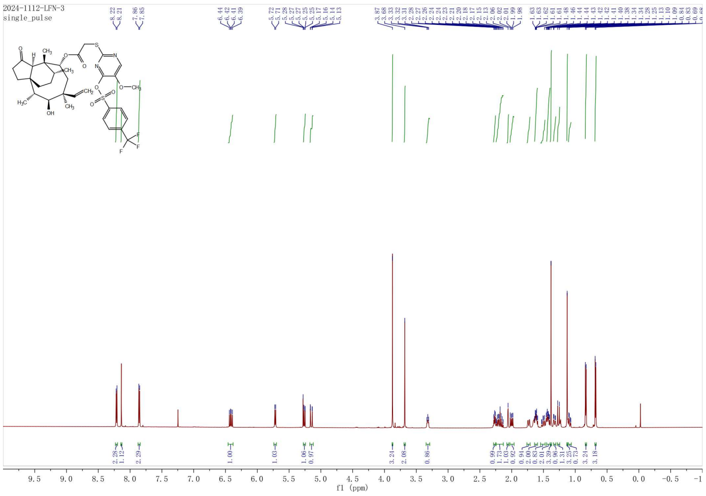

Figure S12. ^{1}H NMR (600 MHz $CDCl_3$) spectrum of compound **J3**.

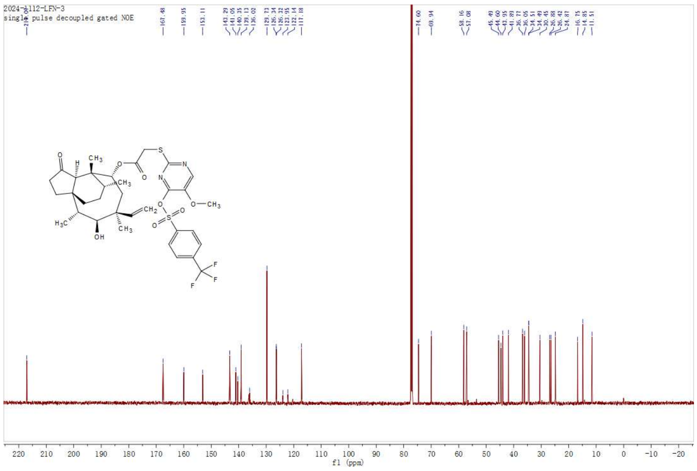

Figure S13. ^{13}C NMR (151 MHz $CDCl_3$) spectrum of compound **J3**.

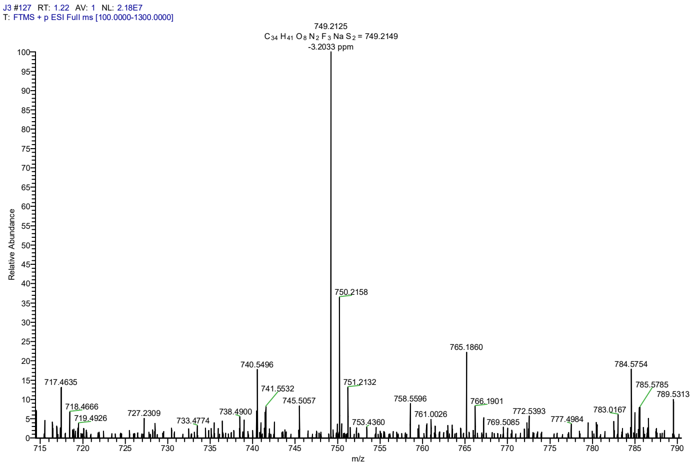

Figure S14. HRMS of compound **J3**.

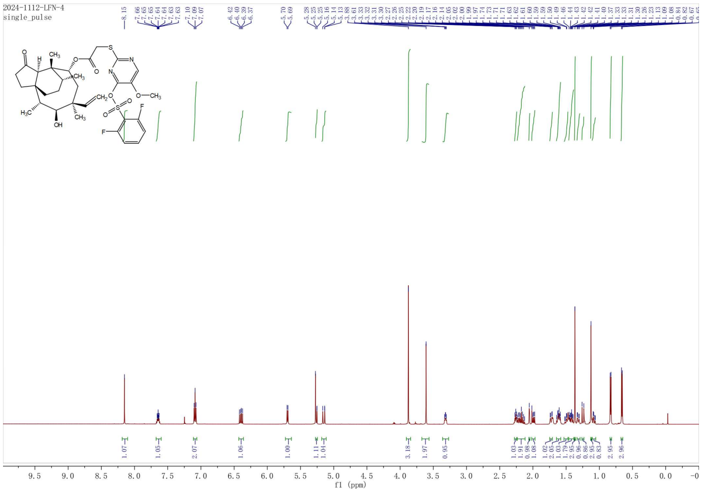

Figure S15. ^{1}H NMR (600 MHz $CDCl_3$) spectrum of compound **J4**.

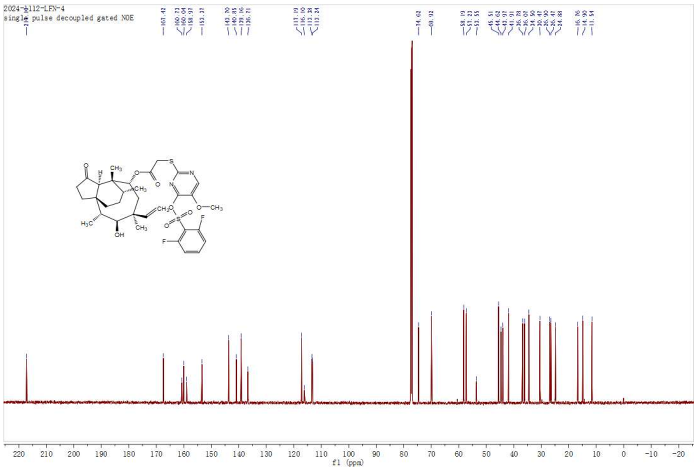

Figure S16. ^{13}C NMR (151 MHz $CDCl_3$) spectrum of compound **J4**.

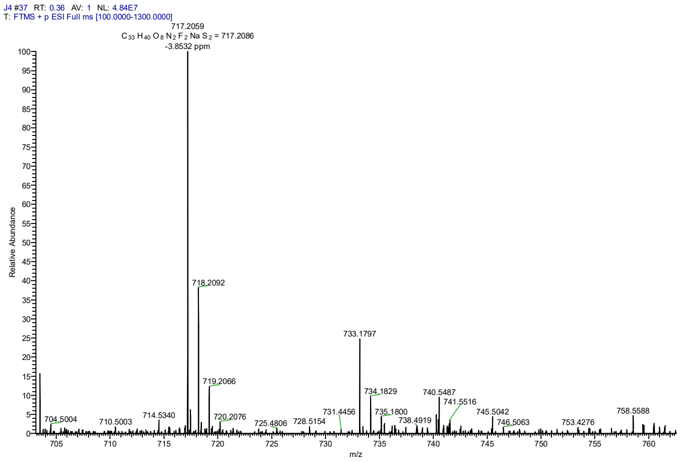

Figure S17. HRMS of compound **J4**.

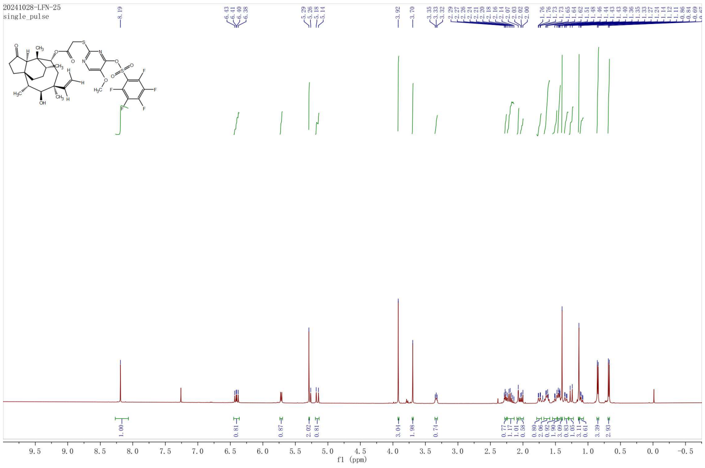

Figure S18.^{1}H NMR (500 MHz $CDCl_3$) spectrum of compound **J5**.

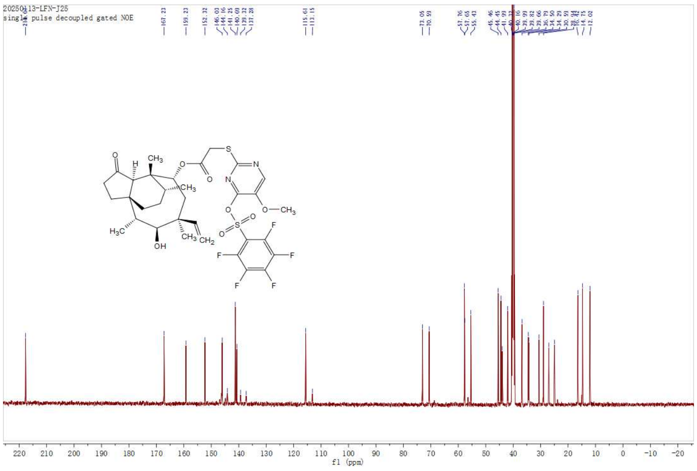

Figure S19. ^{13}C NMR (126 MHz DMSO) spectrum of compound **J5**.

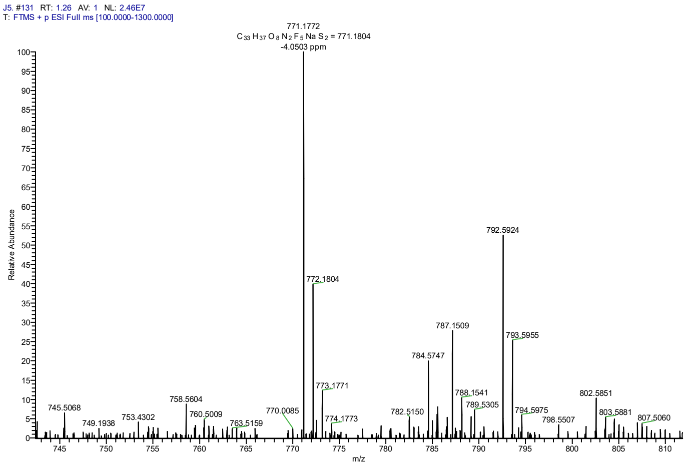

Figure S20. HRMS of compound **J5**.

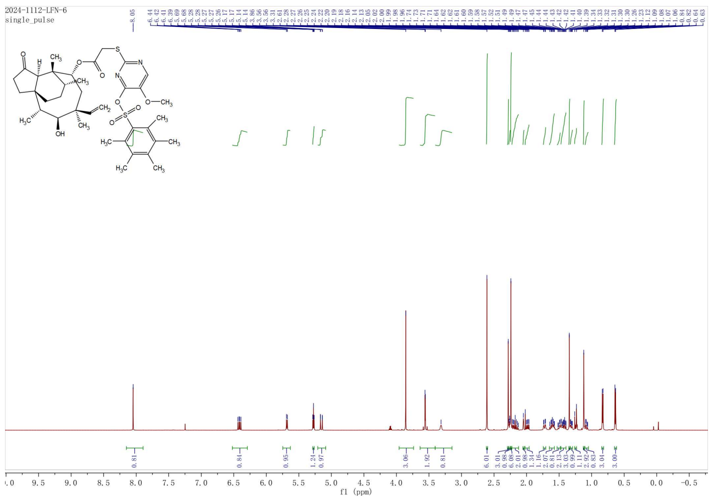

Figure S21. ^{1}H NMR (600 MHz $CDCl_3$) spectrum of compound **J6**.

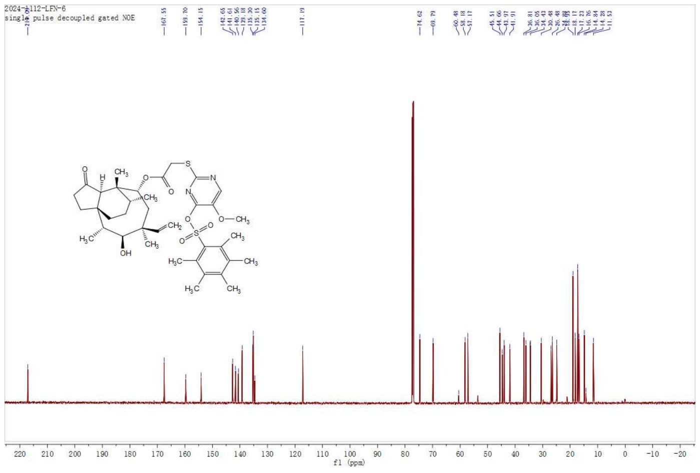

Figure S22. ^{13}C NMR (151 MHz $CDCl_3$) spectrum of compound **J6**.

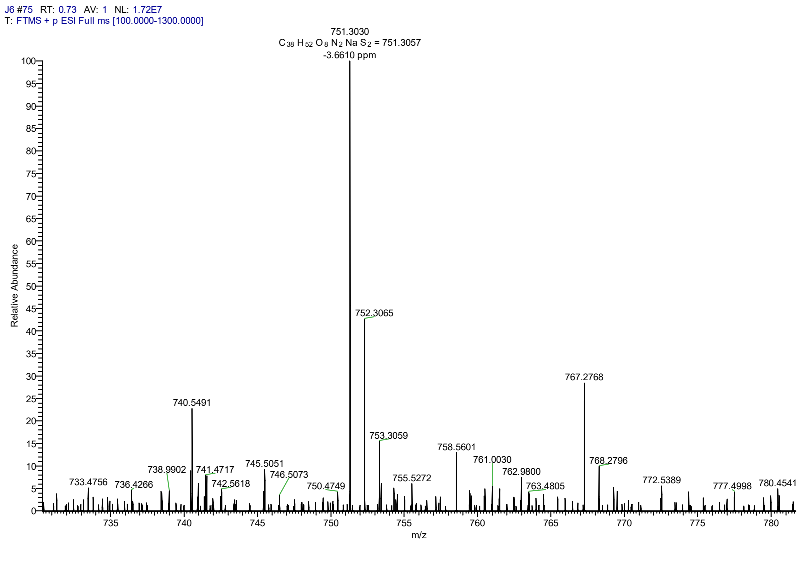

Figure S23. HRMS of compound **J6**.

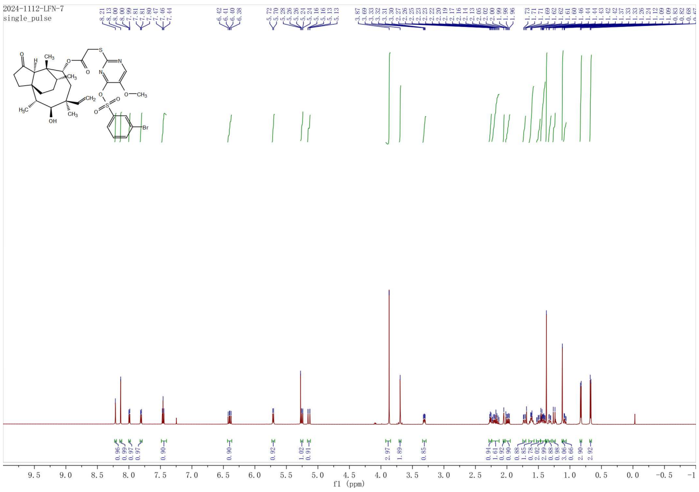

Figure S24. ^{1}H NMR (600 MHz $CDCl_3$) spectrum of compound **J7**.

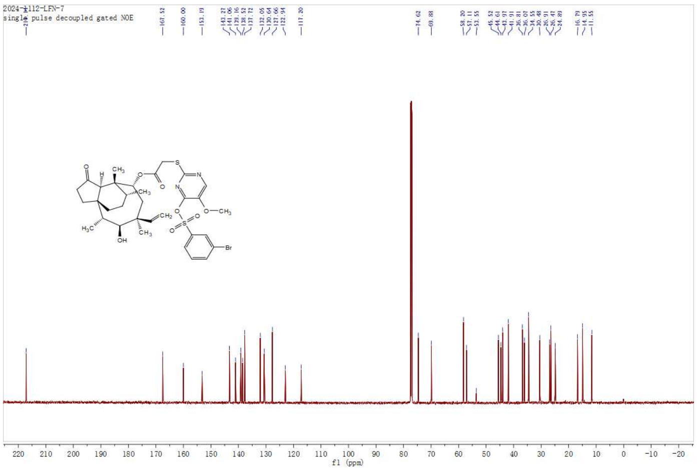

Figure S25. ^{13}C NMR (151 MHz $CDCl_3$) spectrum of compound **J7**.

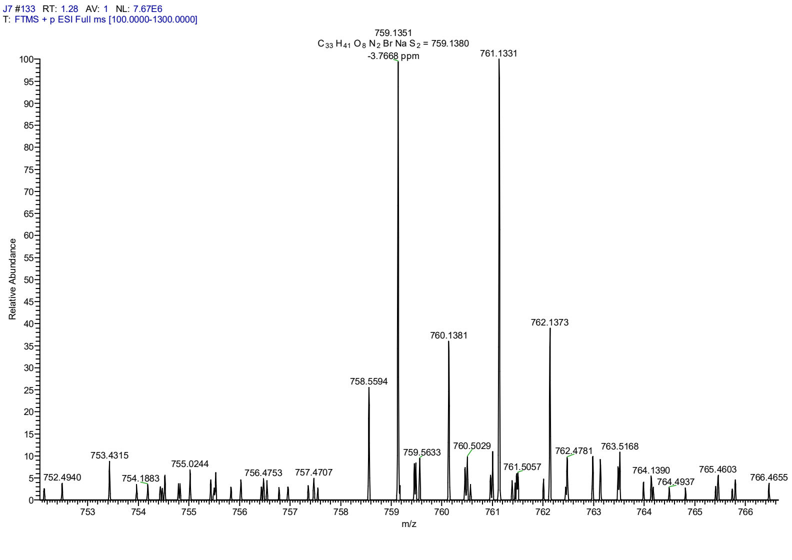

Figure S26. HRMS of compound **J7**.

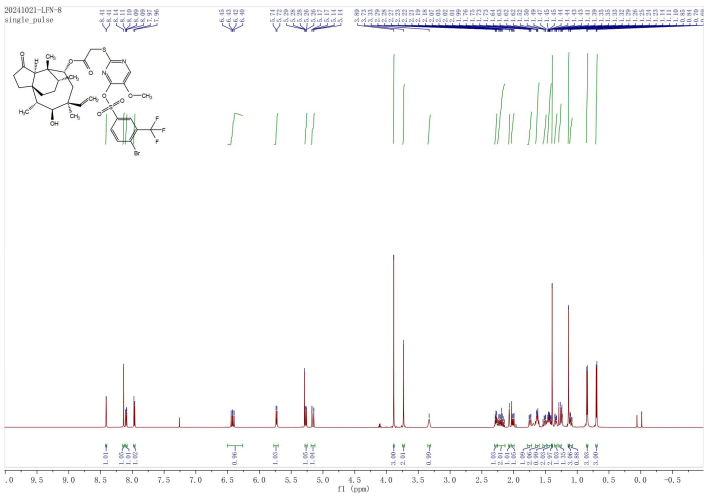

Figure S27. ^{1}H NMR (600 MHz $CDCl_3$) spectrum of compound **J8**.

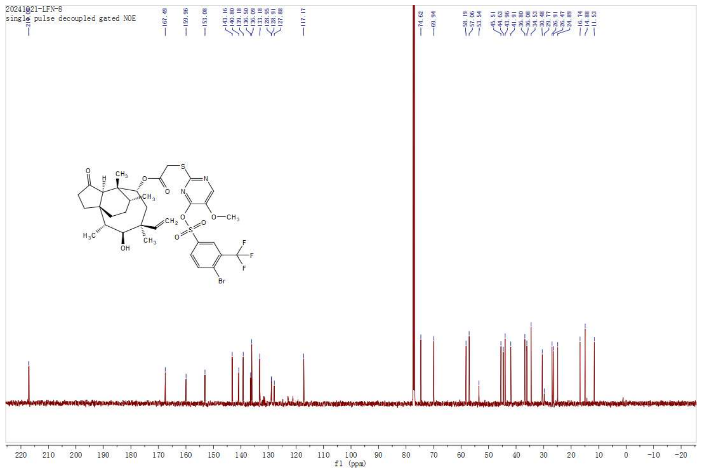

Figure S28. ^{13}C NMR (151 MHz $CDCl_3$) spectrum of compound **J8**.

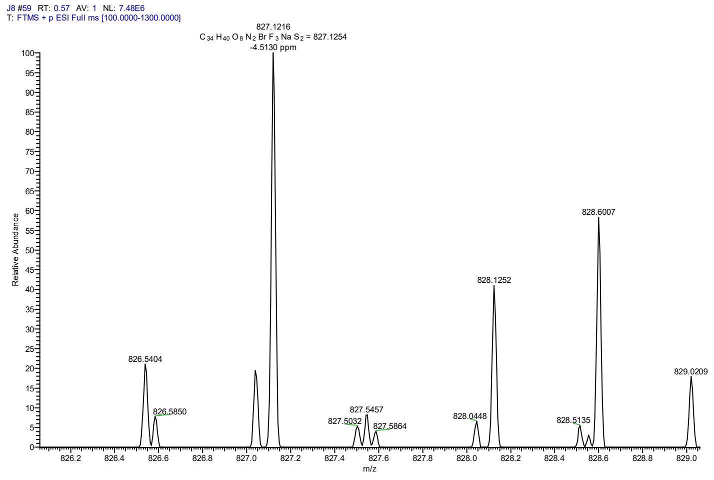

Figure S29. HRMS of compound **J8**.

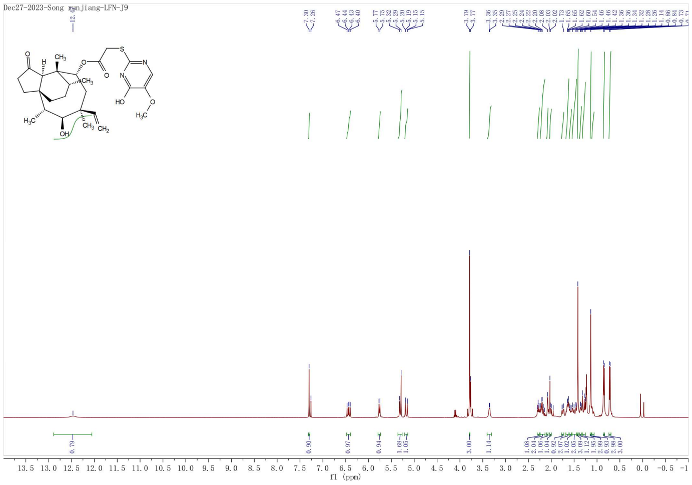

Figure S30. ^{1}H NMR (400 MHz $CDCl_3$) spectrum of compound **J9**.

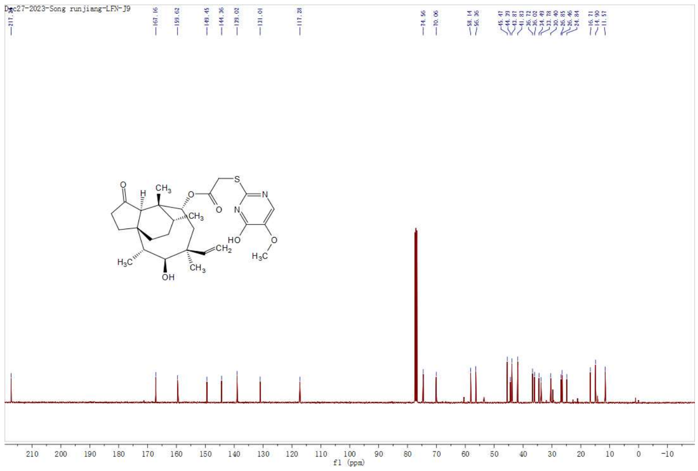

Figure S31. ^{13}C NMR (101 MHz $CDCl_3$) spectrum of compound **J9**.

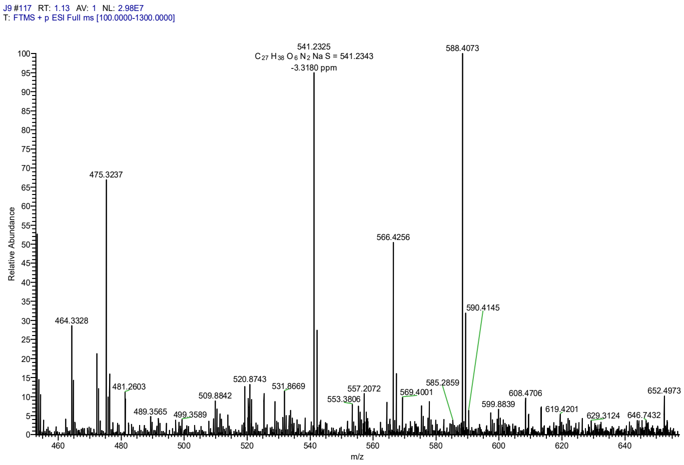

Figure S32. HRMS of compound **J9**.

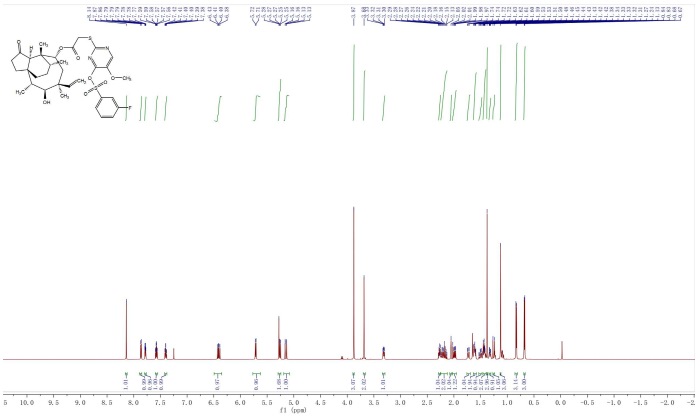

Figure S33. ^{1}H NMR (600 MHz $CDCl_3$) spectrum of compound **J10**.

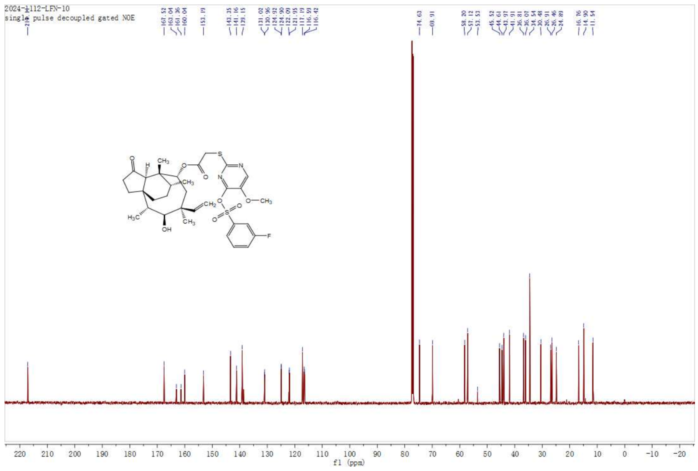

Figure S34. ^{13}C NMR (151 MHz $CDCl_3$) spectrum of compound **J10**.

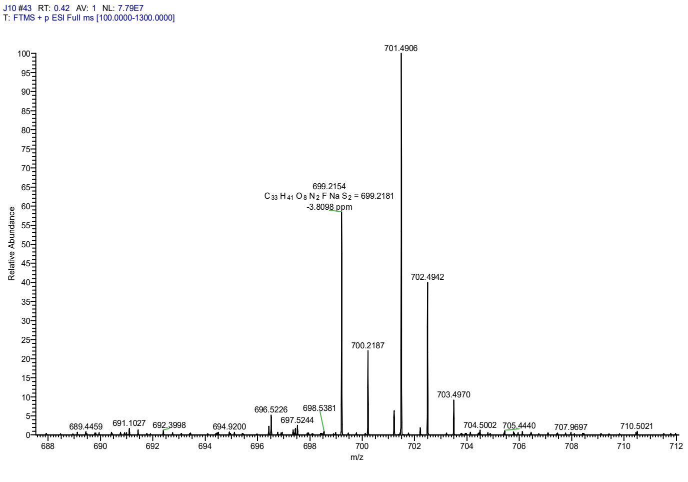

Figure S35. HRMS of compound **J10**.

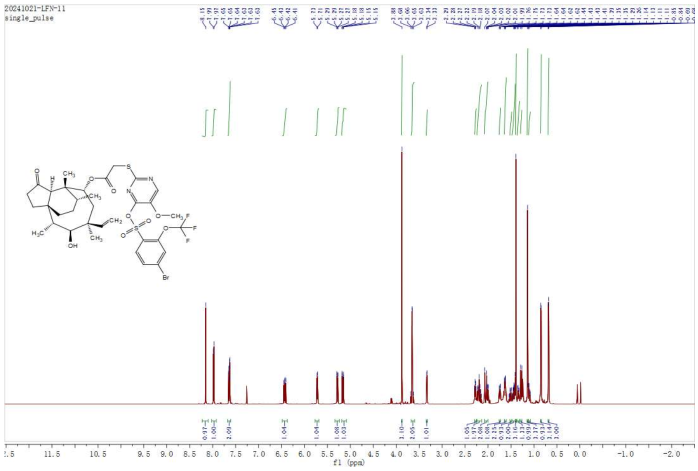

Figure S36. ^{1}H NMR (600 MHz $CDCl_3$) spectrum of compound **J11**.

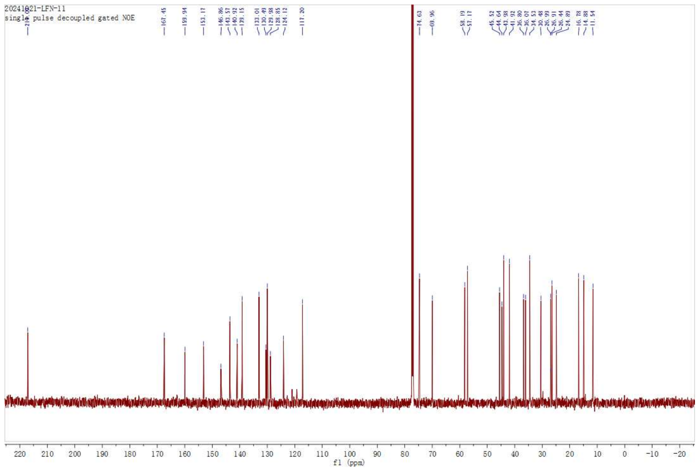

Figure S37. ^{13}C NMR (151 MHz $CDCl_3$) spectrum of compound **J11**.

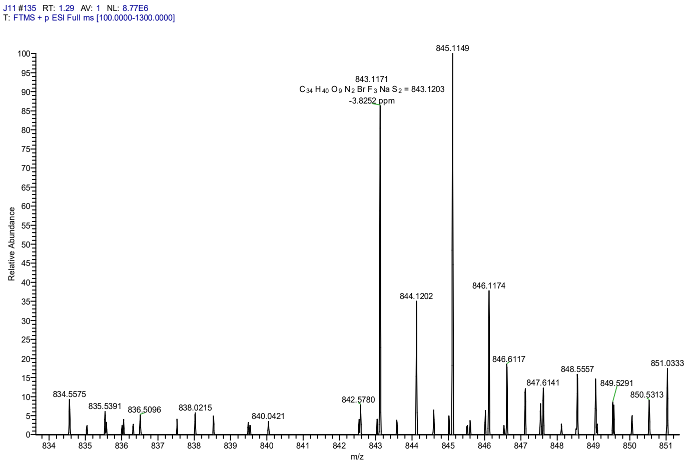

Figure S38. HRMS of compound **J11**.

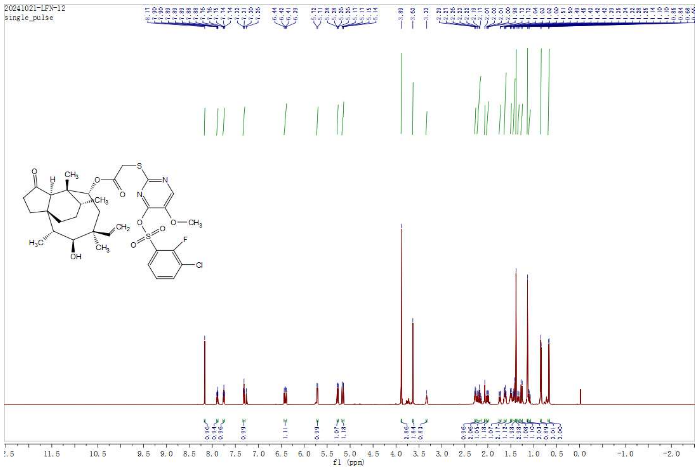

Figure S39. ^{1}H NMR (600 MHz $CDCl_3$) spectrum of compound **J12**.

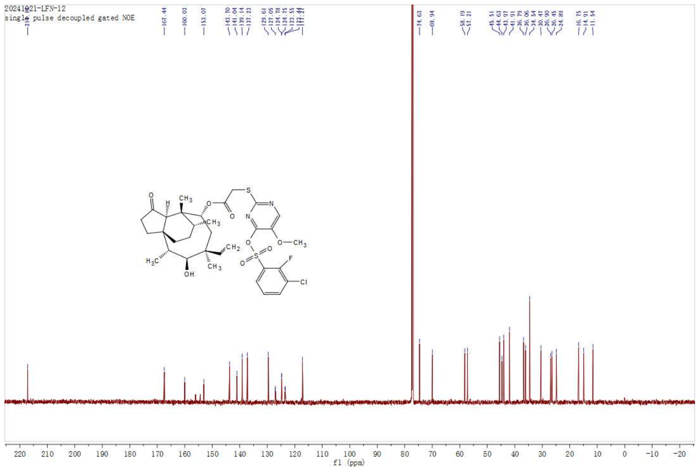

Figure S40 ^{13}C NMR (151 MHz $CDCl_3$) spectrum of compound **J12**.

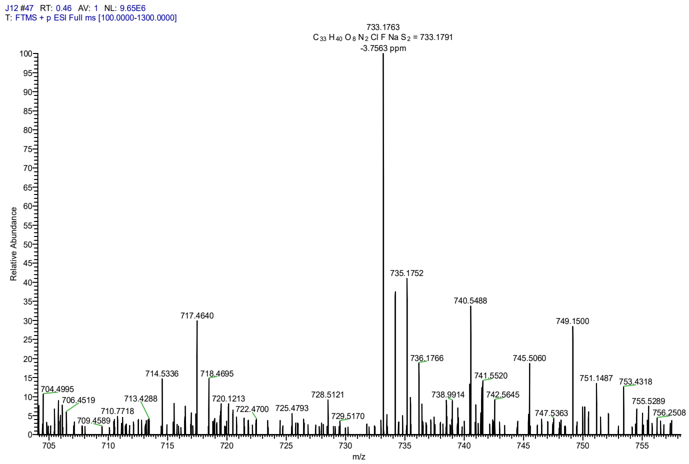

Figure S41. HRMS of compound **J12**.

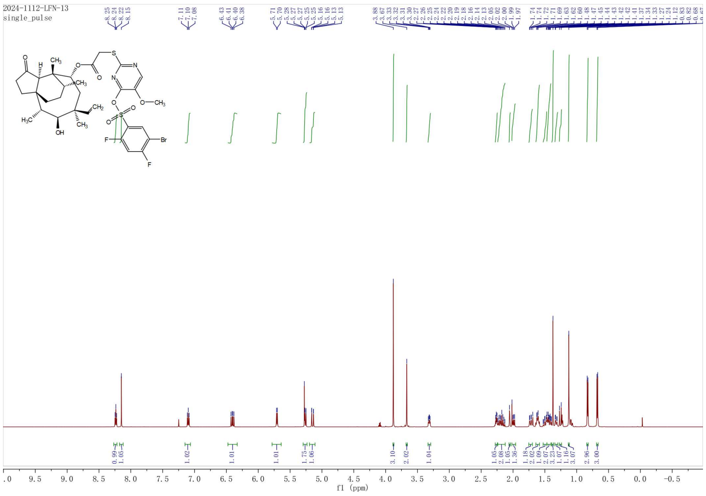

Figure S42. ^{1}H NMR (600 MHz $CDCl_3$) spectrum of compound **J13**.

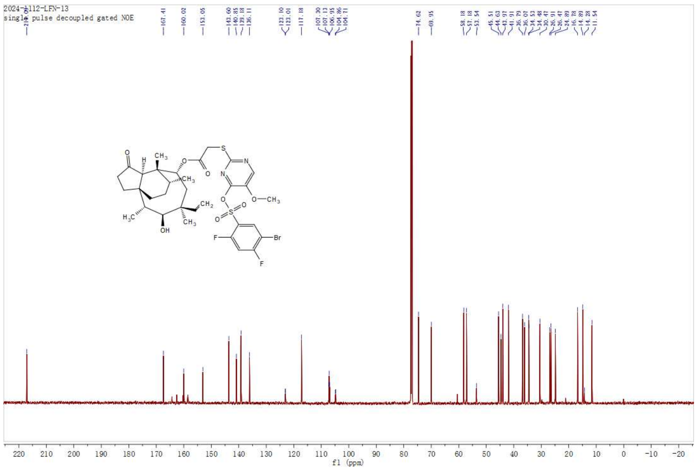

Figure S43. ^{13}C NMR (151 MHz $CDCl_3$) spectrum of compound **J13**.

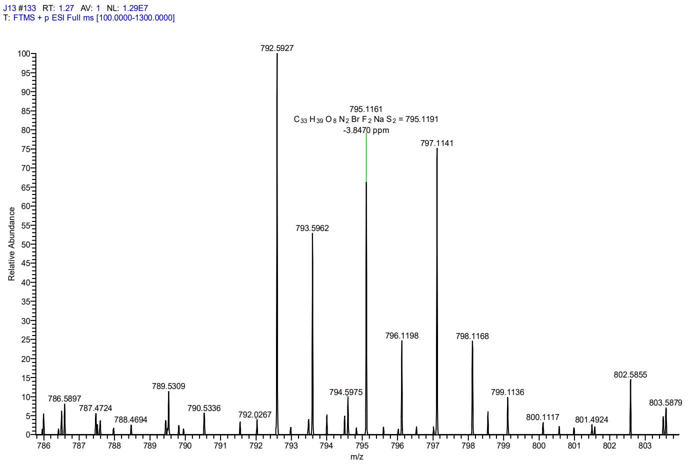

Figure S44. HRMS of compound **J13**.

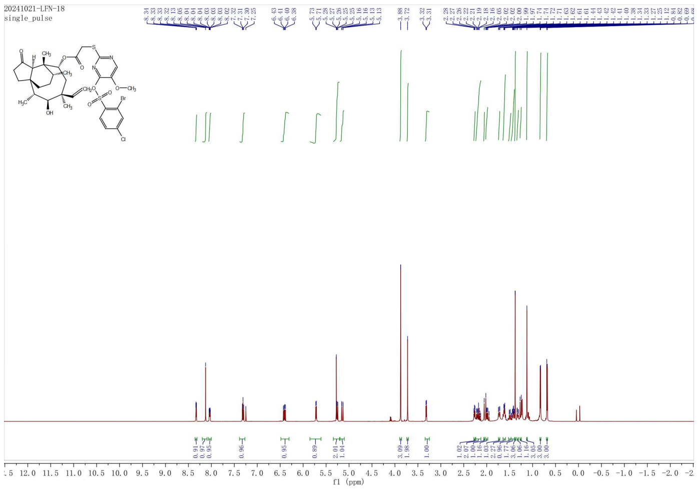

Figure S45. ^{1}H NMR (600 MHz $CDCl_3$) spectrum of compound **J14**.

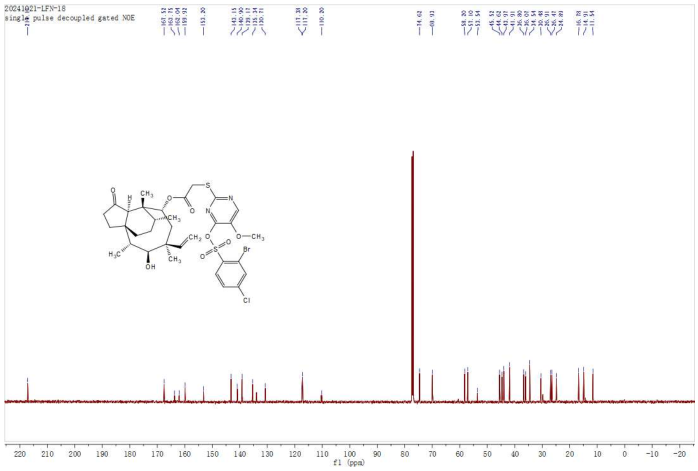

Figure S46.^{13}C NMR (151 MHz $CDCl_3$) spectrum of compound **J14**.

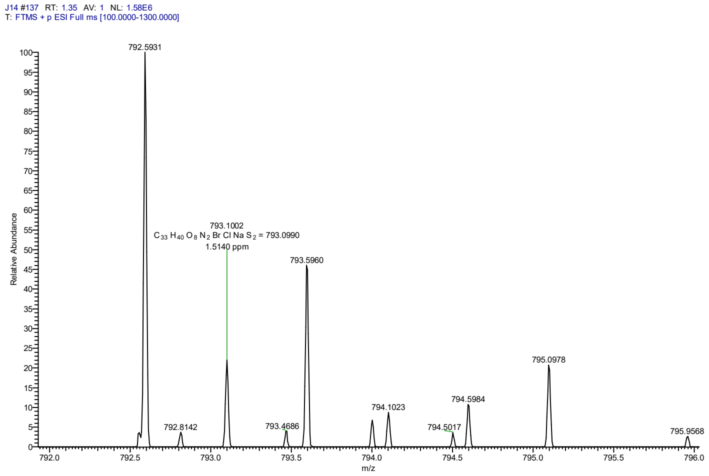

Figure S47. HRMS of compound **J14**.

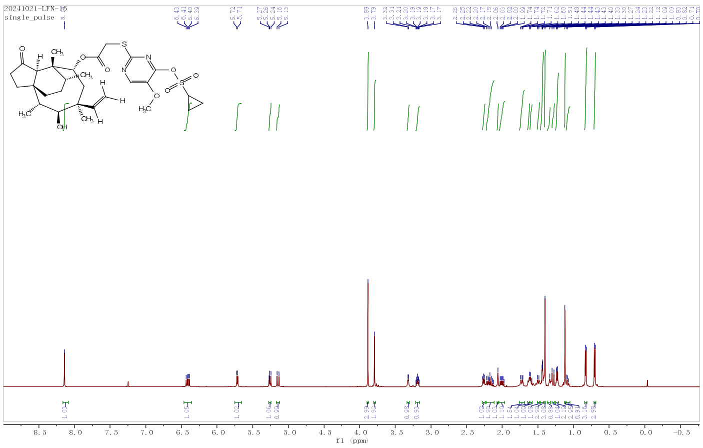

Figure S48. ^{1}H NMR (600 MHz $CDCl_3$) spectrum of compound **J15**.

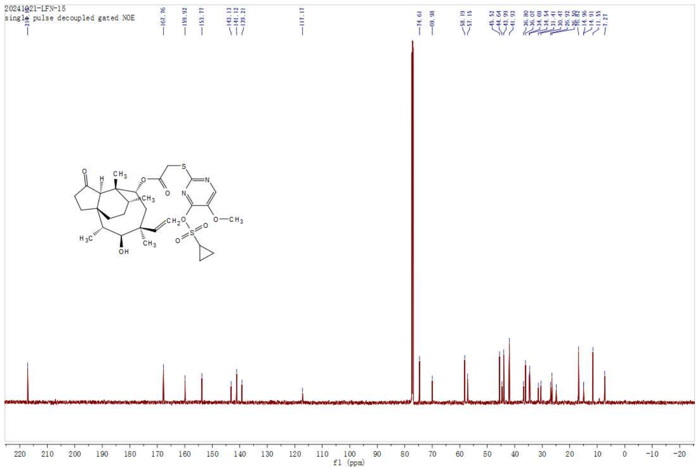

Figure S49. ^{13}C NMR (151 MHz $CDCl_3$) spectrum of compound **J15**.

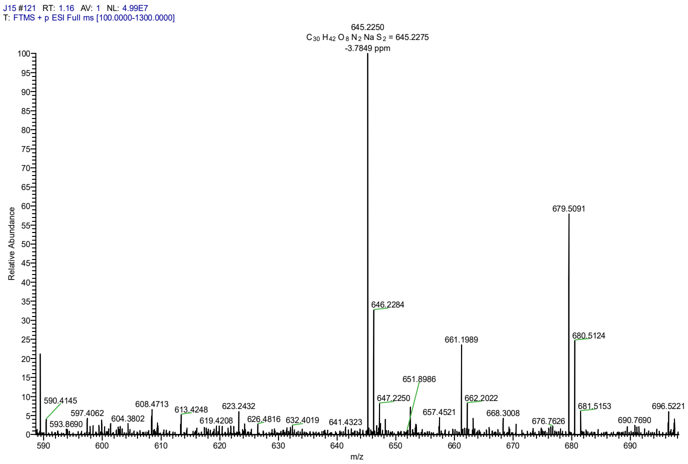

Figure S50. HRMS of compound **J15**.

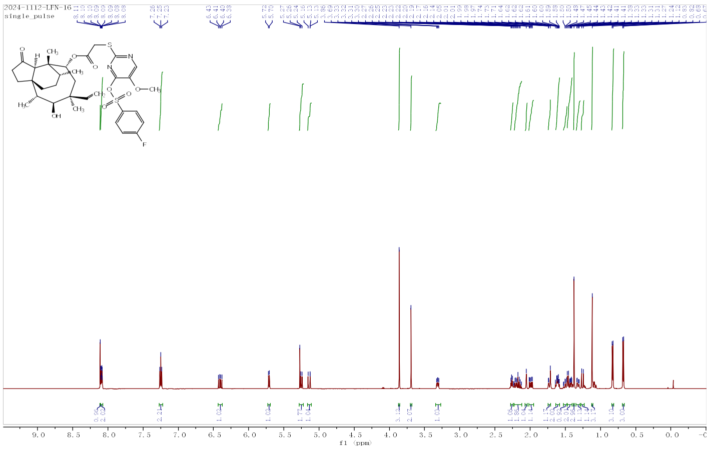

Figure S51. ^{1}H NMR (600 MHz $CDCl_3$) spectrum of compound **J16**.

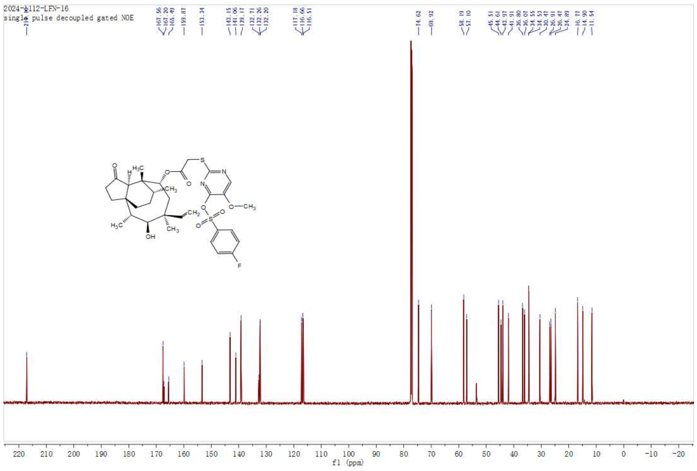

Figure S52. ^{13}C NMR (151 MHz $CDCl_3$) spectrum of compound **J16**.

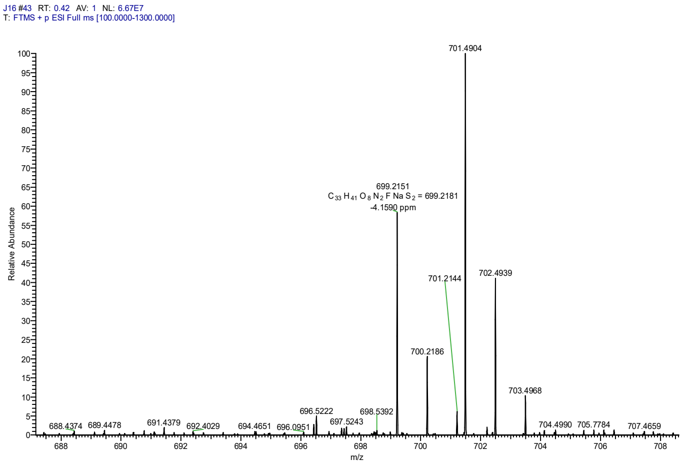

Figure S53. HRMS of compound **J16**.

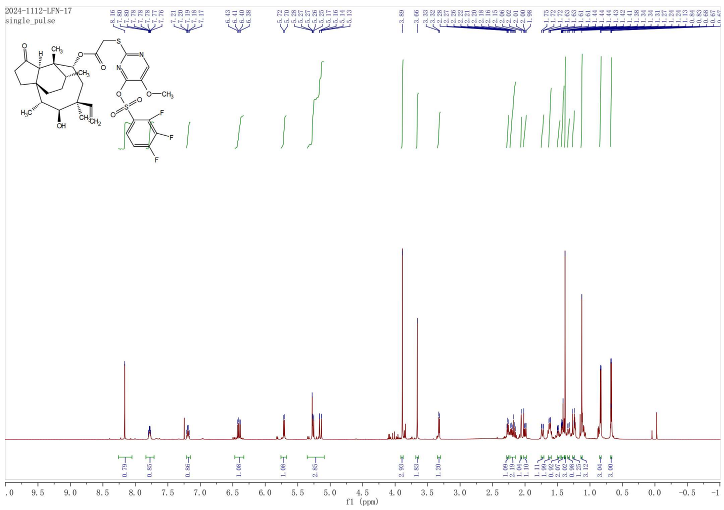

Figure S54. ^{1}H NMR (600 MHz $CDCl_3$) spectrum of compound **J17**.

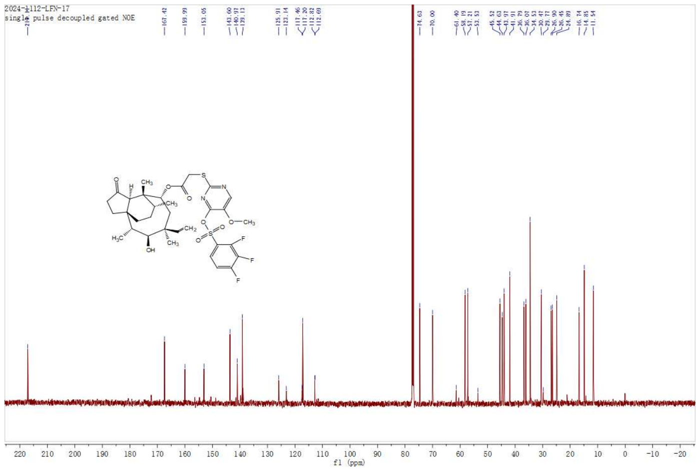

Figure S55. ^{13}C NMR (151 MHz $CDCl_3$) spectrum of compound **J17**.

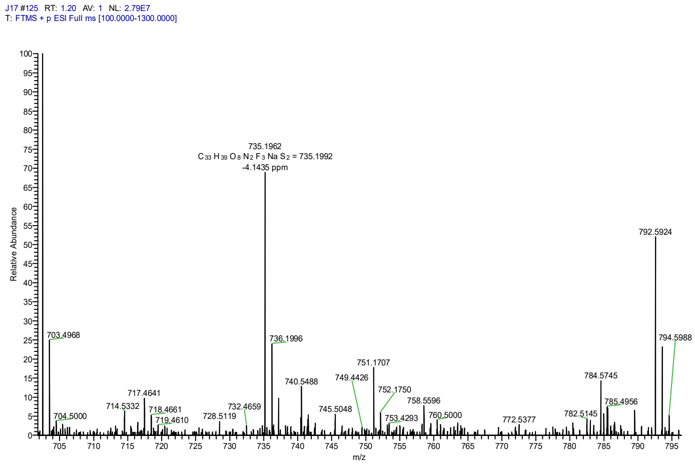

Figure S56. HRMS of compound **J17**.

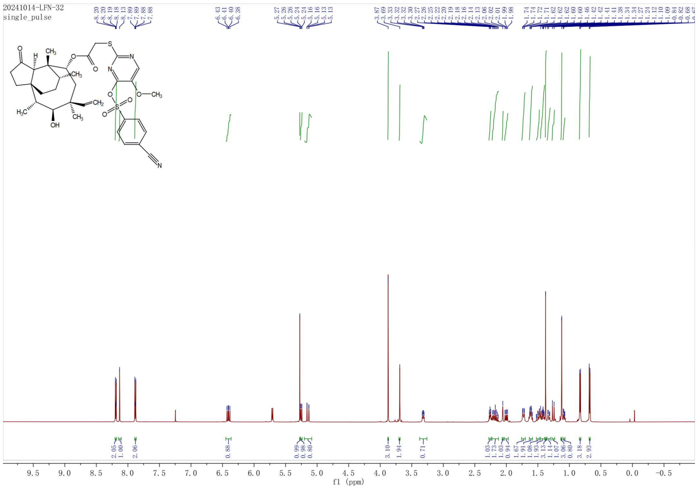

Figure S57. ^{1}H NMR (600 MHz $CDCl_3$) spectrum of compound **J18**.

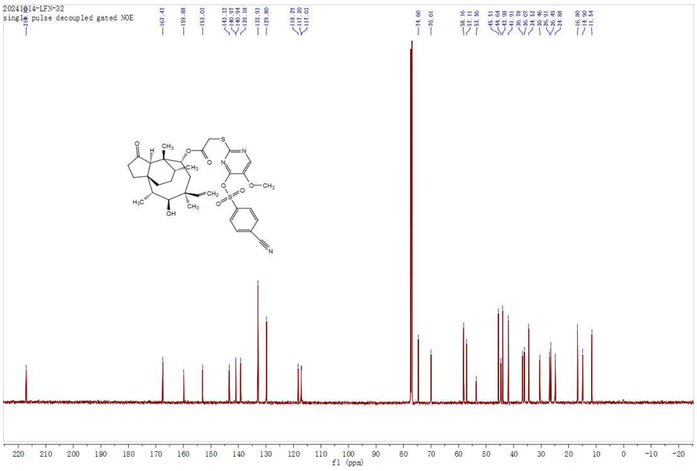

Figure S58. ^{13}C NMR (151 MHz $CDCl_3$) spectrum of compound **J18**.

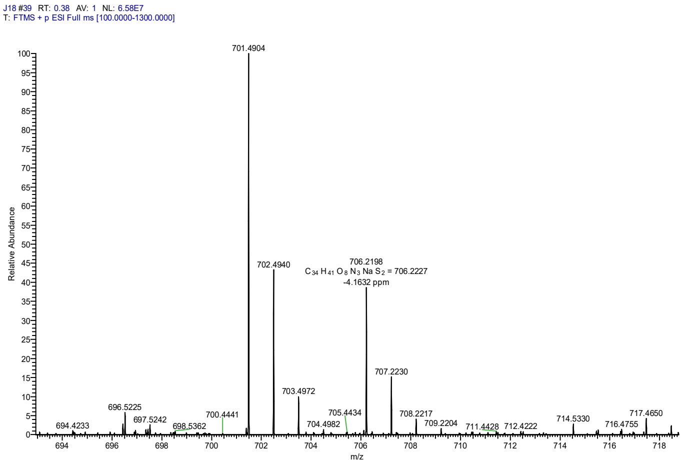

Figure S59. HRMS of compound **J18**.

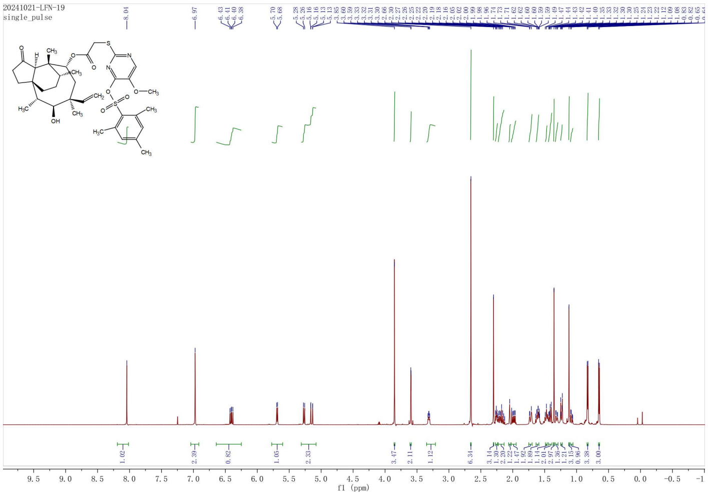

Figure S60. ^{1}H NMR (600 MHz $CDCl_3$) spectrum of compound **J19**.

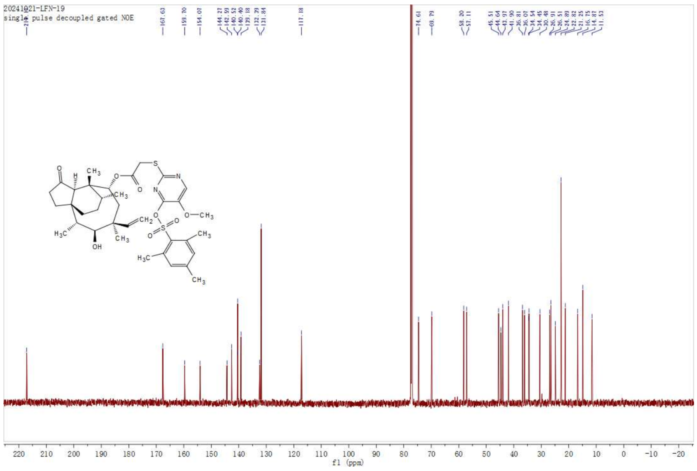

Figure S61. ^{13}C NMR (151 MHz $CDCl_3$) spectrum of compound **J19**.

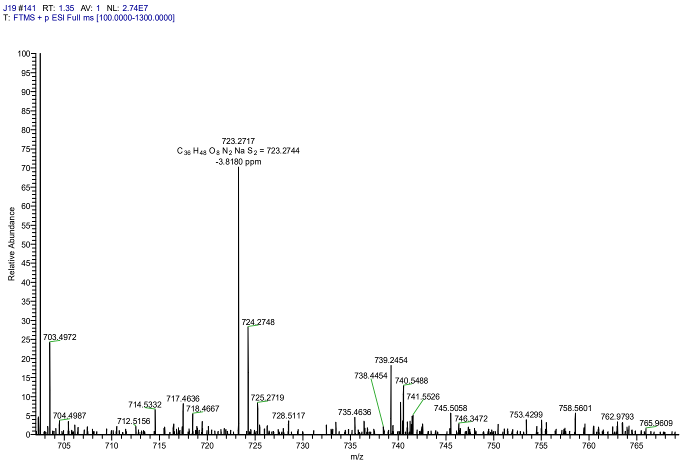

Figure S62. HRMS of compound **J19**.

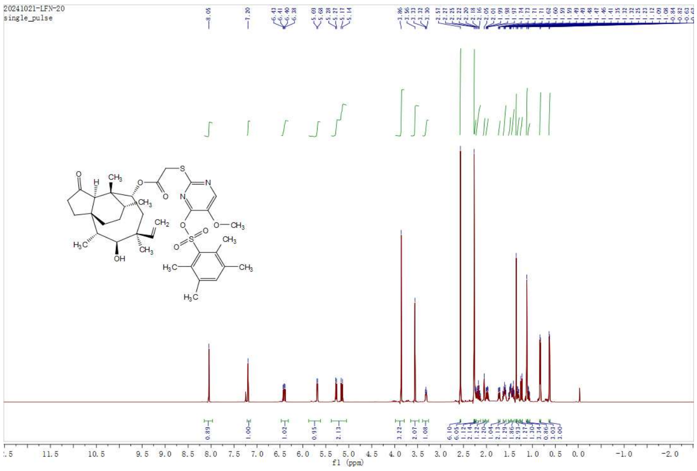

Figure S63. ^{1}H NMR (600 MHz $CDCl_3$) spectrum of compound **J20**.

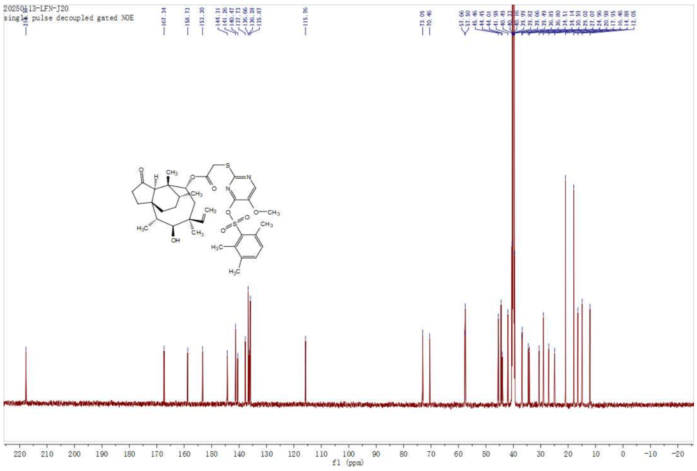

Figure S64. ^{13}C NMR (126 MHz DMSO) spectrum of compound **J20**.

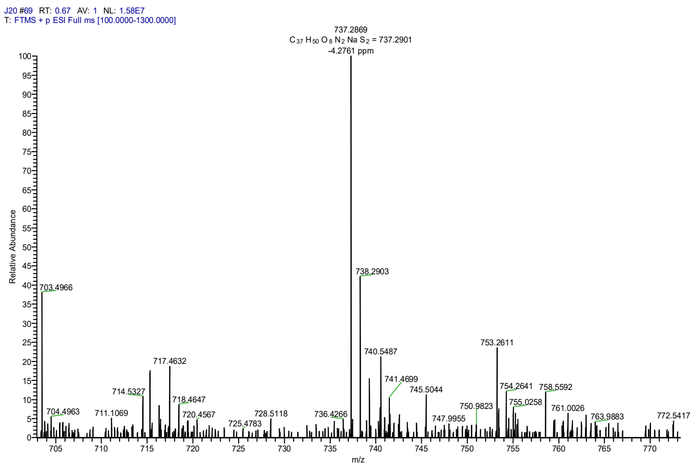

Figure S65. HRMS of compound **J20**.

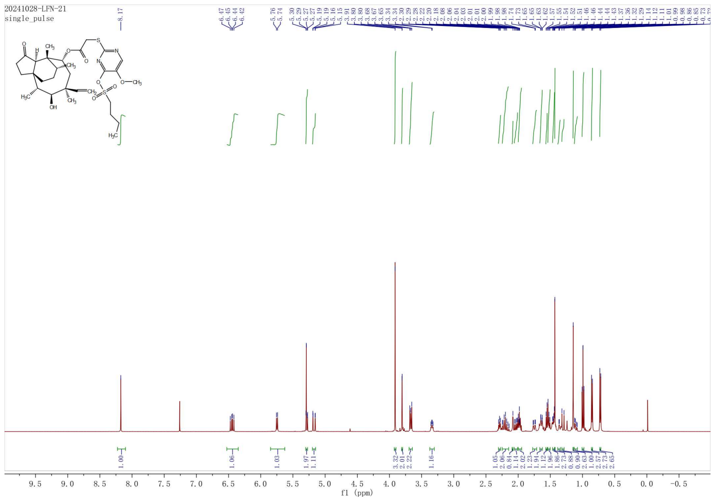

Figure S66. ^{1}H NMR (400 MHz $CDCl_3$) spectrum of compound **J21**.

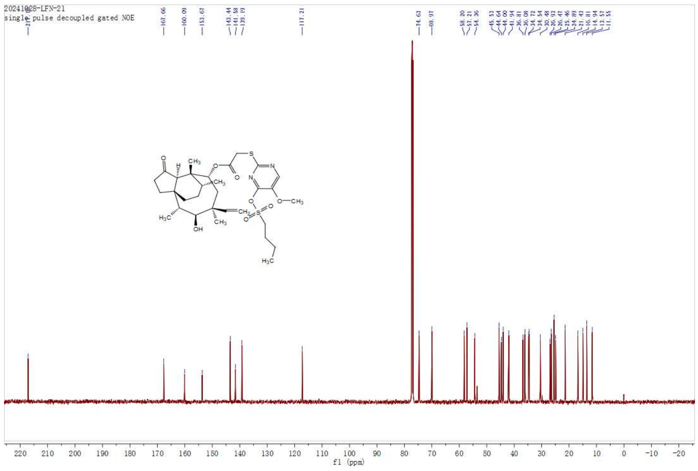

Figure S67. ^{13}C NMR (126 MHz $CDCl_3$) spectrum of compound **J21**.

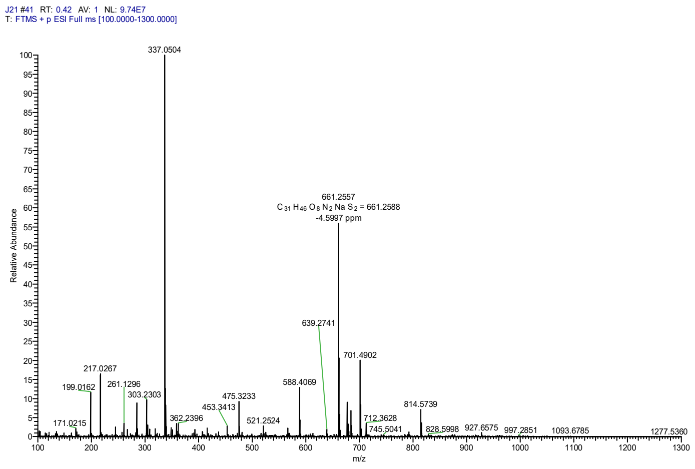

Figure S68. HRMS of compound J**21**.

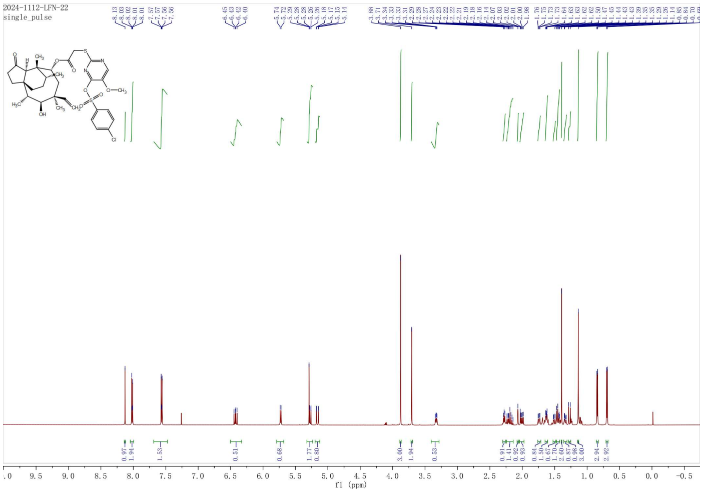

Figure S69 ^{1}H NMR (600 MHz $CDCl_3$ spectrum of compound **J22**.

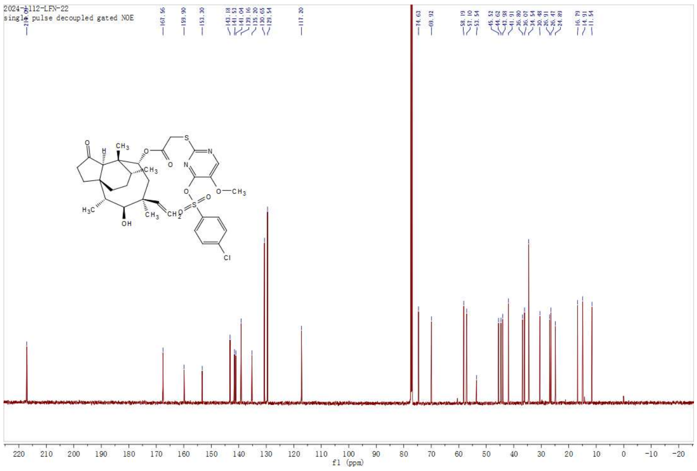

Figure S70. ^{13}C NMR (151 MHz $CDCl_3$) spectrum of compound **J22**.

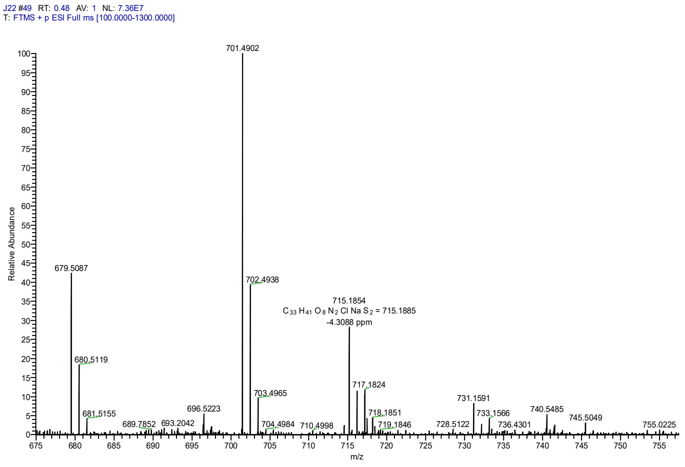

Figure S71. HRMS of compound **J22**.

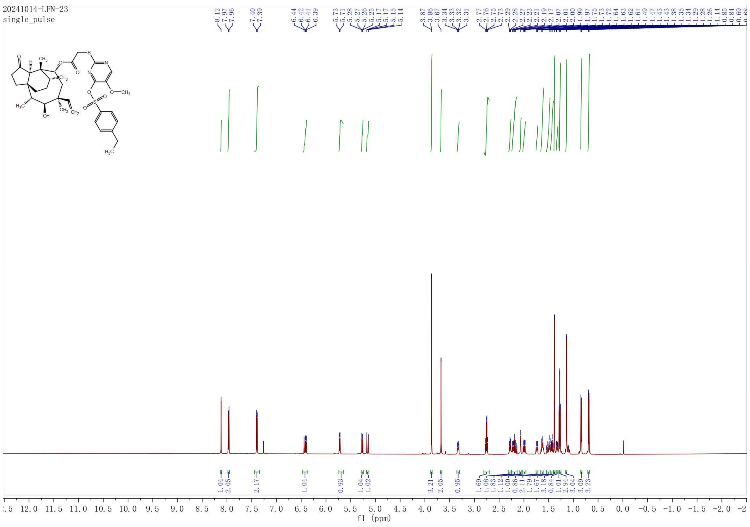

Figure S72. ^{1}H NMR (600 MHz $CDCl_3$) spectrum of compound **J23**.

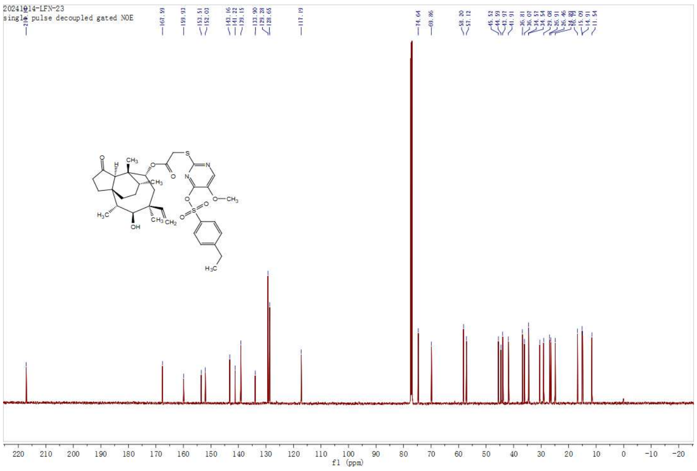

Figure S73. ^{13}C NMR (151 MHz $CDCl_3$) spectrum of compound **J23**.

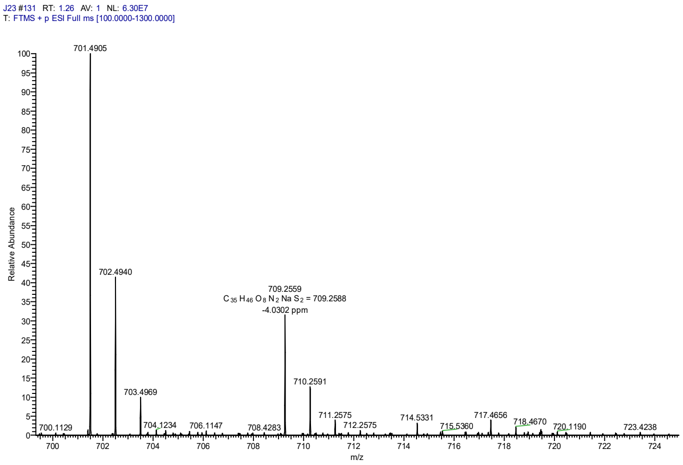

Figure S74. HRMS of compound **J23**.

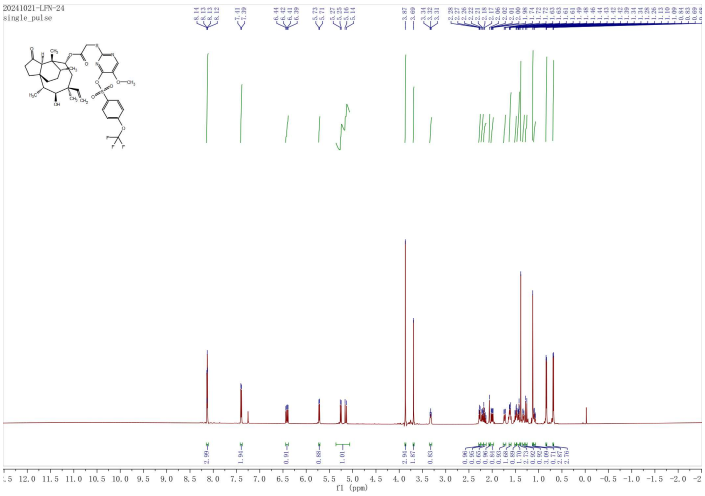

Figure S75. ^{1}H NMR (600 MHz $CDCl_3$) spectrum of compound **J24**.

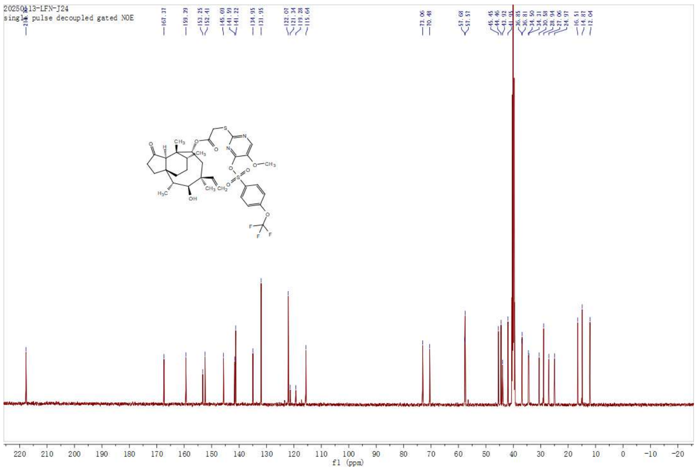

Figure S76. ^{13}C NMR (151 MHz DMSO-d_6) spectrum of compound **J24**.

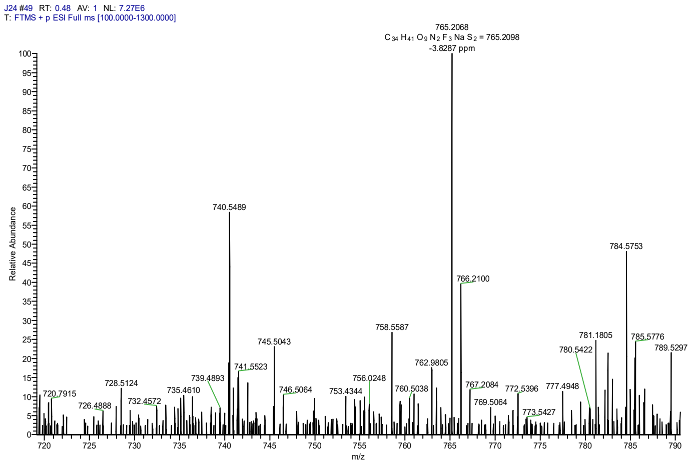

Figure S77. HRMS of compound **J24**.

V. Protein purification

The amino acid sequence of ISXoo15:

MSINSIARRLNRNACTLSREIRRQGAPGYAATSAASNYRLRRRACVRRRRLVEGSAIFQQV
RDDLVLYRWSPQQIAAKLKAMHPDDPSQRVSHETIYTAIYTHPRGGLKKELVEALRQHKP
TRGLRRTTAAKRTWVPEELRIVHRPEEVAQRLIPGHWEGDLIKGAFNRSCVGTLVERKTRF
VVLCKMDGCTPQDALEGFTRQMKKLPHCLLGSLTYDRGTEMTCYPELMKRLNIDLWFA
DPHAPWQRGSNENTNGLLCQFMPKGVDLSKASQEYLNNVADLMNARPRQTLGWKTPN
QALEEEIAQFNSRVALAS

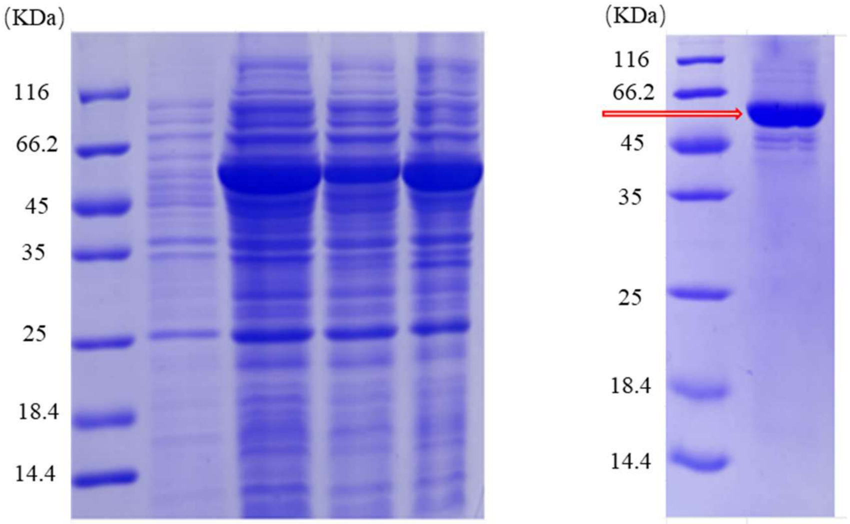

Figure S78. SDS-PAGE analysis of purified ISXoo15 transposase

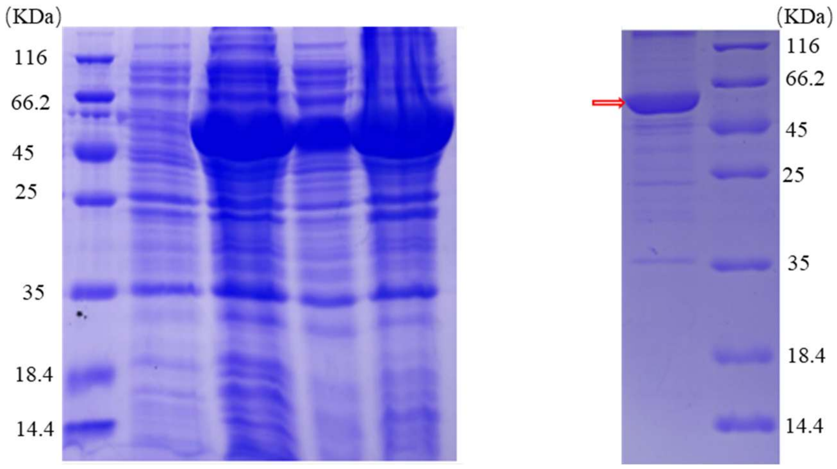

Figure S79. SDS-PAGE analysis of purified ISXoo15 transposase^{-A244W}
